# Supplementary material for: The MondoA-dependent TXNIP/GDF15 axis predicts oxaliplatin response in colorectal adenocarcinomas
Source: EMBO Mol Med. 2024 Aug 5;16(9):7. doi: 10.1038/s44321-024-00105-2 (PMC11393413; doi:10.1038/s44321-024-00105-2)
Supplement: Supplementary file 4 — Appendix [file 44321_2024_105_MOESM4_ESM.pdf]

Table of contents.

Appendix Figure S1 – Page 1
Appendix Figure S2 – Page 2
Appendix Figure S3 – Page 3
Appendix Figure S4 – Page 4
Appendix Figure S5 – Page 5
Appendix Figure S6 – Page 6
Appendix Figure S7 – Page 7
Appendix Table S1 – Page 9
Appendix Table S2 – Page 11
Appendix Table S3 – Page 12
Appendix Table S4 – Page 16
Appendix Table S5 – Page 17
Appendix Table S6 – Page 18
Appendix Table S7 – Page 19

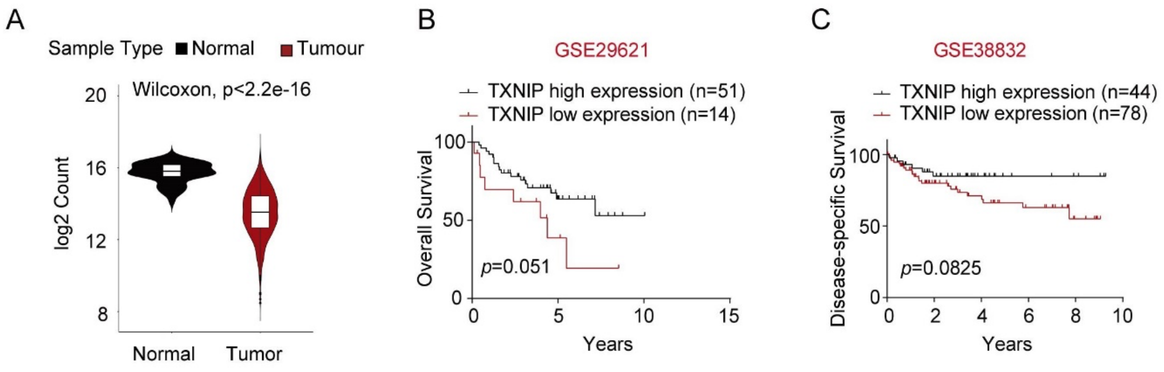

Appendix Figure S1. *TXNIP* expression is lower in colorectal cancer samples compared to normal tissues. (A) Analysis of The Cancer Genomic Atlas (TCGA) Colon Adenocarcinoma (COAD) database. Comparative analysis of *TXNIP* transcript expression between adjacent normal tissue and cancer tissues. (B-C) Kaplan-Meier analysis of overall survival (B) and distant metastasis-free survival (C) in CRC patients with different *TXNIP* mRNA expression levels. Wilcoxon rank-sum test p value indicated.

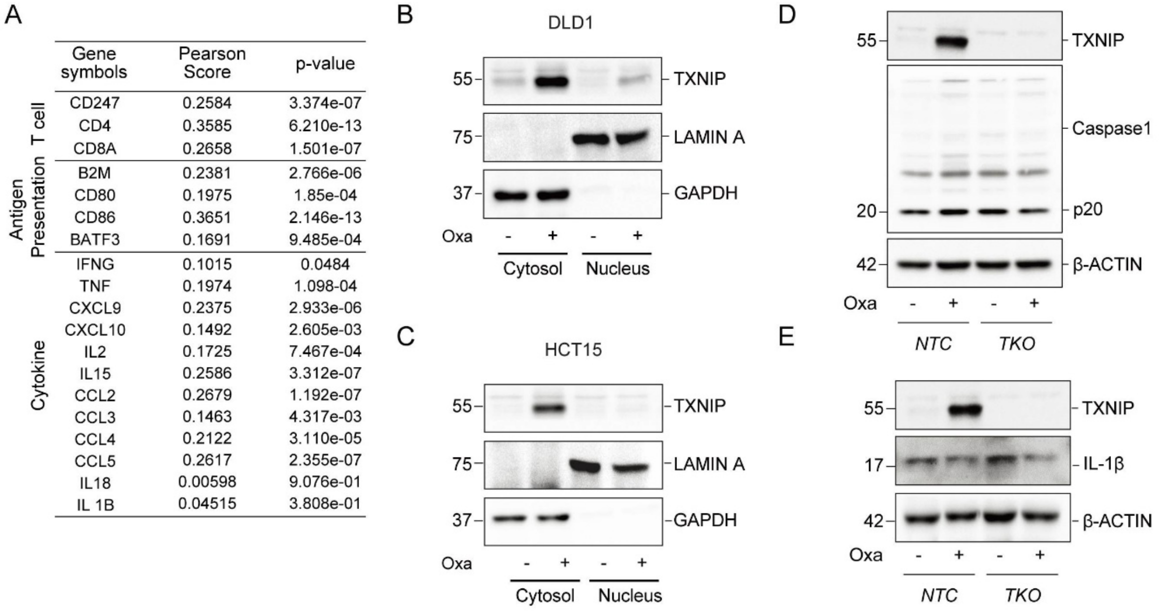

	Gene symbols	Pearson Score	p-value
T cell	CD247	0.2584	3.374e-07
	CD4	0.3585	6.210e-13
	CD8A	0.2658	1.501e-07
Antigen Presentation	B2M	0.2381	2.766e-06
	CD80	0.1975	1.85e-04
	CD86	0.3651	2.146e-13
	BATF3	0.1691	9.485e-04
Cytokine	IFNG	0.1015	0.0484
	TNF	0.1974	1.098-04
	CXCL9	0.2375	2.933e-06
	CXCL10	0.1492	2.605e-03
	IL2	0.1725	7.467e-04
	IL15	0.2586	3.312e-07
	CCL2	0.2679	1.192e-07
	CCL3	0.1463	4.317e-03
	CCL4	0.2122	3.110e-05
	CCL5	0.2617	2.355e-07
	IL18	0.00598	9.076e-01
	IL 1B	0.04515	3.808e-01

Appendix Figure S2. TXNIP is associated with immune activation, which is independent of inflammasome activity. (A) Pearson correlation coefficient scores and p values showing the relationship between *TXNIP* transcript expression and different immune marker transcript expression; including T cell markers (*CD247, CD4, CD8A*), antigen presentation markers (*B2M, CD80, CD86, BATF3*) and cytokines (*IFNG, TNF, CXCL9, CXCL10, IL2, IL15, CCL2, CCL3, CCL4, CCL5, IL18, IL1B*) from the TCGA COAD dataset. (B-C) Effects of oxaliplatin (10µm for 48h) on subcellular localization of TXNIP assessed by cell fractionation and immunoblotting in DLD1 cells (B) and HCT15 cells (C). (D-E) Immunoblot analysis of cleaved caspase 1(p20) (D) and IL-1β (E) in control (NTC) and *TXNIP*-KO (TKO) DLD1 cells with/ without 10µm oxaliplatin treatment for 48h. Results shown are representative of three independent experiments.

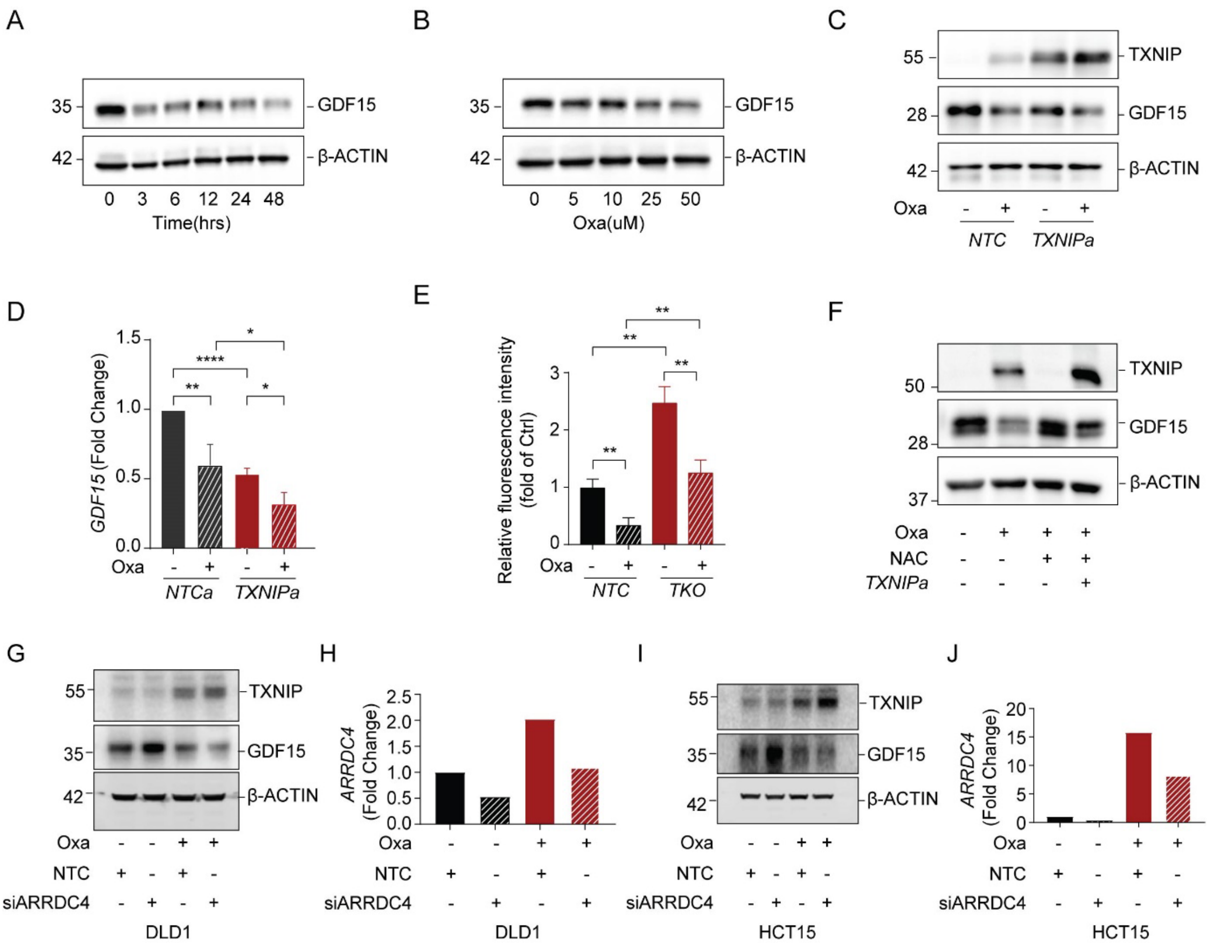

Appendix Figure S3. Oxaliplatin treatment, TXNIP and ARRDC4 suppress GDF15 expression. (A-B) Immunoblotting of GDF15 in DLD1 cells after treatment with 10µm oxaliplatin at indicated time points. (A); after treatment of different dosages of oxaliplatin for 48 hours (B). (C-D) Immunoblotting of TXNIP and GDF15 in control (NTC) and *TXNIP*-overexpressing (*TXNIP*a) DLD1 cells with or without 10µm oxaliplatin treatment for 48h (C); pooled densiometric data from C (D). Standard error bars are shown n=3. (E) Quantitation of immunofluorescence from Figure 3I (GDF15 levels relative to cell area) from 3 independent experiments. (F) Immunoblotting of TXNIP and GDF15 in *TXNIP*a or NTC cells treated with oxaliplatin (10µm) or combined treatment with oxaliplatin and NAC (1.25mM) for 48h. (G-J) DLD1 cells (G-H) or HCT15 cells (I-J) were treated with siARRDC4 or NTC +/- 10mM oxaliplatin for 48h. TXNIP and GDF15 protein expression was measured by Western, with b-actin as a loading control (G, I). *ARRDC4* transcript expression was measured using RT-qPCR. Expression was normalised to GAPDH and fold change compared to NTC treated cells shown (H, J). Results shown are representative of three independent experiments. All values were expressed as mean ± SEM. *p<0.1, **p<0.01, ****p < 0.0001, vs. Control.

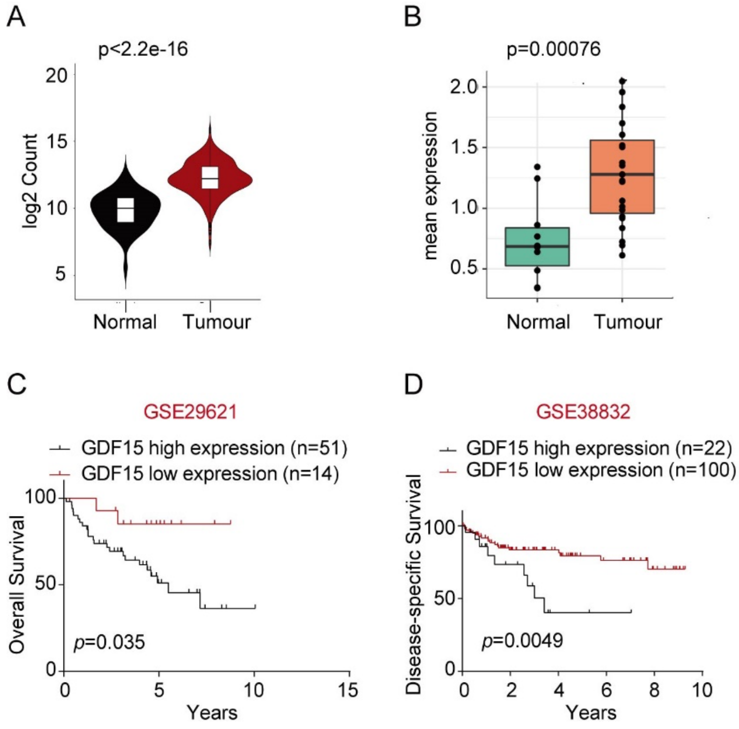

Appendix Figure S4. *GDF15* expression is higher in colorectal cancer samples compared to normal tissues. (A) Analysis of The Cancer Genomic Atlas (TCGA) Colon Adenocarcinoma (COAD) database. Comparative analysis of expression of *GDF15* between adjacent normal tissue and cancer tissues. Wilcoxon rank-sum test p value indicated. (B) *GDF15* transcript expression in single epithelial cells derived from matched primary CRC tumors and adjacent normal colon (n=10 pairs). (C-D) Kaplan-Meier analysis of overall survival (C) and distant metastasis-free survival (D) in CRC patients with different *GDF15* mRNA expression levels.

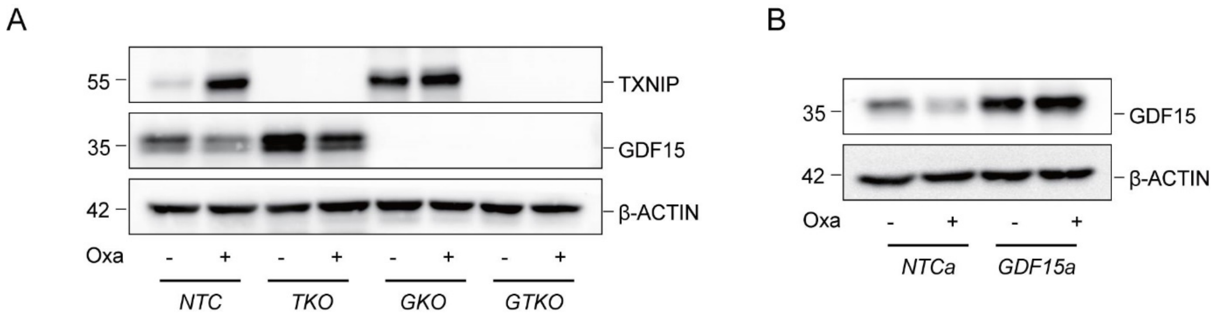

Appendix Figure S5. Establishment of knock-out and over-expressing DLD1 cell models (A) Immunoblot of TXNIP and GDF15 expression in NTC, *GDF15* knockout (GKO), TKO, *GDF15* and *TXNIP* knockout (GTKO) DLD1 cell lines after 48h of oxaliplatin treatment (10µm). (B) Immunoblot of GDF15 expression in *GDF15*-CRISPRa (*GDF15*a) DLD1 cell line in the presence of 10µm oxaliplatin for 48h. Results shown are representative of three independent experiments.

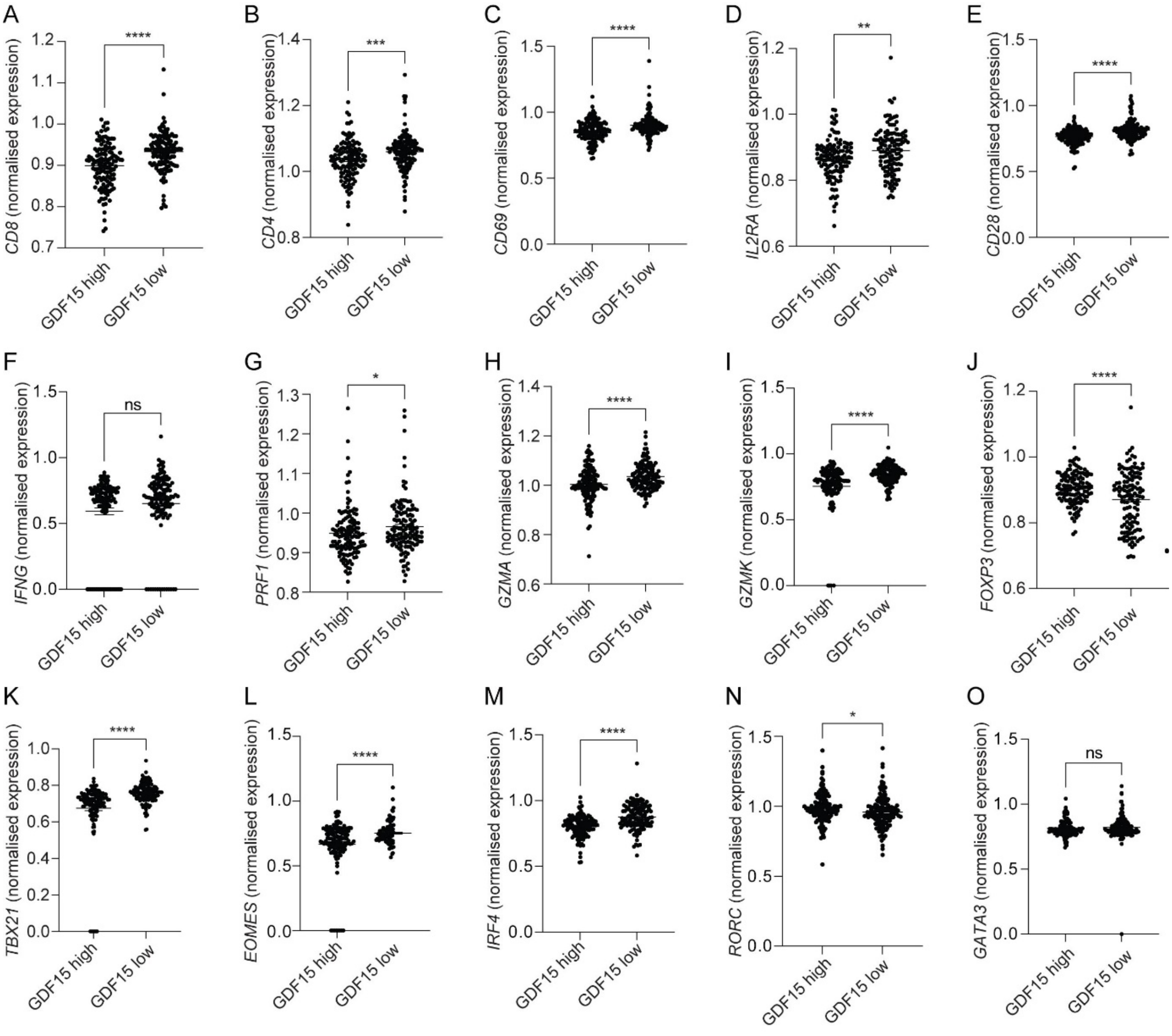

Appendix Figure S6. Low *GDF15* expressing tumors express more activated cytotoxic CD8 T cell transcripts. Analysis of TCGA COAD database. Comparative analysis of *CD8A* (A), *CD4* (B), *CD69* (C), *IL2RA* (D), *CD28* (E), *IFNG* (F), *PRF1* (G), *GZMA* (H), *GZMK* (I), *FOXP3* (J), *TBX21* (K), *EOMES* (L), *IRF4* (M), *RORC* (N) and *GATA3* (O) transcript expression between high *GDF15* tumors and low *GDF15* tumors. Normalised expression = transcript expression normalised to the mean of (CD3D+ CD3E)/2. *GDF15* high and low groups were defined as the top and bottom quartiles when cases were ranked by *GDF15* expression. Two-tailed Student's t test; *$p<0.05$, **$p<0.01$, ***$p < 0.001$, ****$p < 0.0001$.

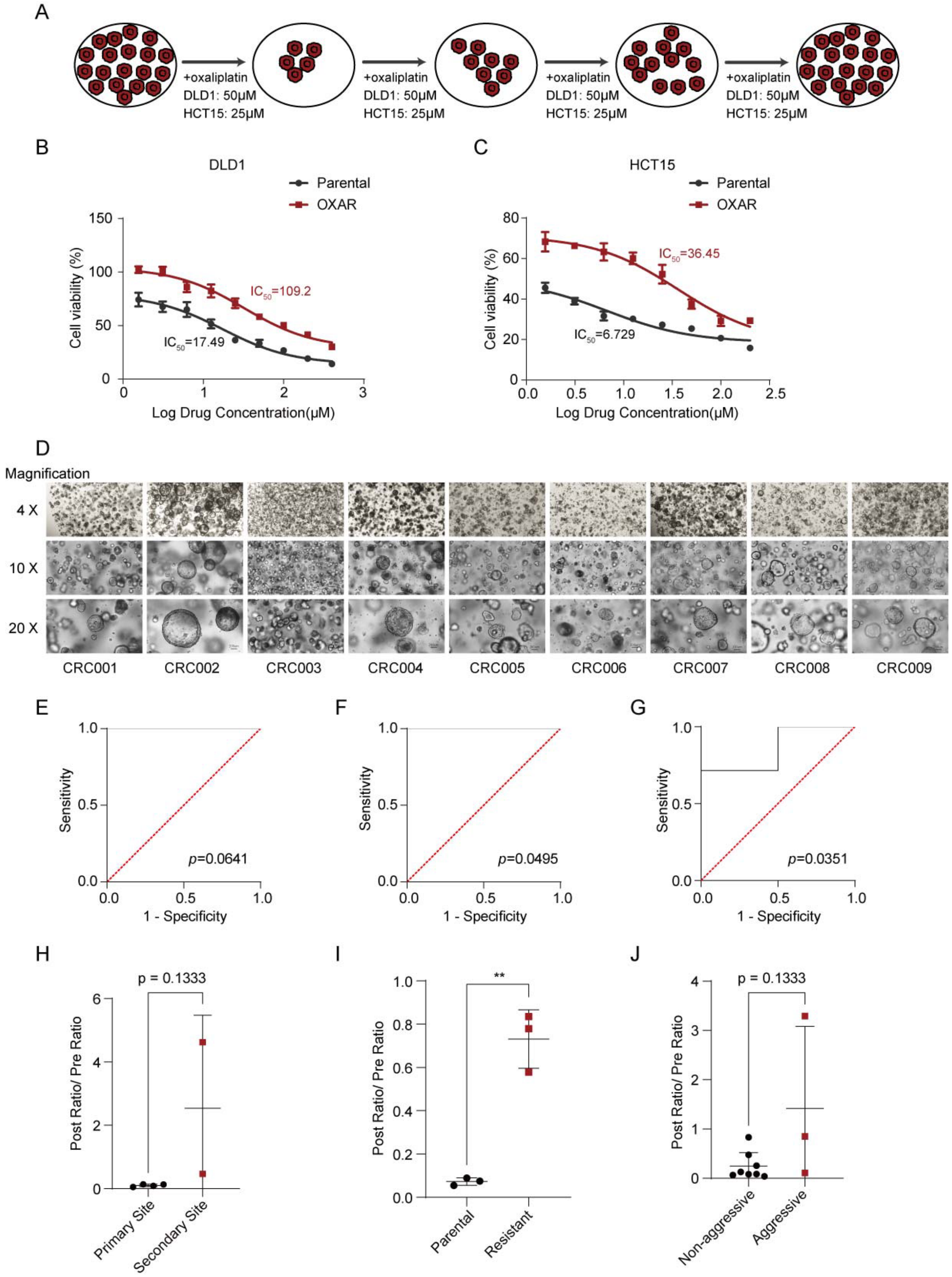

Appendix Figure S7. Establishment of oxaliplatin-resistant cell lines and patient-derived tumor organoids. (A) A schematic model showing the process by which oxaliplatin-resistant CRC cells were generated. (B-C) IC50 values of oxaliplatin in oxaliplatin-resistant cells (OXAR) and their parental cells. DLD1 and DLD1-OXAR (B); HCT15 and HCT15-OXAR (C). (D) Bright field images of different organoids at different magnifications. (E-G) Receiver

operating characteristic (ROC) curves showing area under the curve and p values for the use of GDF15/TXNIP ratio in predicting origin of cell line (E; primary; DLD1, HCT15, HT29, SW48 [n=4] or secondary; DiFi, LIM1215 [n=2]), sensitivity to oxaliplatin (F; parental DLD1 (plus biological repeat), HCT15 [n=3] or resistant DLD1 (plus biological repeat), HCT15 [n=3]), aggression of tumor (G; non-aggressive; <T4M1. Patients 1, 4, 5, 7, 8, 9, 11 [n=7] or aggressive; ≥ T4M1. Patients 2, 3, 6 and 10 [n=4]) Please see supplementary table 8 for detailed clinical information for G. (H-J) Post-treatment GDF15/TXNIP ratio divided by pre-treatment GDF15/TXNIP ratio for primary or secondary cell line source (H), parental or resistant cell line (I), or aggression of fresh primary tumor (J). ** $p<0.01$ using unpaired t test. H and J tested using Mann-Whitney.

Appendix Table S1. Clinical information for colorectal cancer cohort; 42 'whole tumor' sections.

Patient No.	Sex	Collect procedure	origin of tissue	Sample pathology diagnosis	Age at diagnosis	TNM stage
B0064258	Female	Tumour mass	sigmoid colon	adenocarcinoma	57	T2N0M0
B0041098	Female	Tumour mass	right hemicolon	adenocarcinoma	39	T2N0M0
B0033411	Male	Tumour mass	left hemicolon	adenocarcinoma	33	T2N0M0
B0026350	Male	biopsy	ascending colon	adenocarcinoma	45	T3N2M1
B0027606	Male	Tumour mass	descending colon	adenocarcinoma	72	T2N1M0
B0027501	Male	Tumour mass	right hemicolon	adenocarcinoma	44	T4N2M1
B0027406	Male	biopsy	sigmoid colon	adenocarcinoma	58	T3N2M1
B0028709	Female	Tumour mass	colon	adenocarcinoma	60	T4N0M0
B0029815	Female	Tumour mass	colon	adenocarcinoma	57	T4N1M1
B0030697	Male	Tumour mass	right hemicolon	adenocarcinoma	55	T2N0M0
B0044409	Female	biopsy	transverse colon	adenocarcinoma	79	T4N2M1
B0047013	Male	Tumour mass	right hemicolon	adenocarcinoma	59	T4N0M0
B0035475	Female	Tumour mass	ileocecum	adenocarcinoma	54	T2N1M0
B0035849	Male	Tumour mass	colon	adenocarcinoma	55	T4N1M0
B0042047	Male	Tumour mass	left hemicolon	adenocarcinoma	56	T2N2M0
B0042815	Female	Tumour mass	right hemicolon	adenocarcinoma	66	T3N1M0
B0045868	Female	Tumour mass	left hemicolon	adenocarcinoma	55	T3N0M0
B0046985	Male	Tumour mass	left hemicolon	adenocarcinoma	57	T3N1M0
B0055650	Male	Tumour mass	transverse colon	adenocarcinoma	54	T4aN0M0
B0056913	Male	Tumour mass	transverse colon	adenocarcinoma	47	T3N1M1
B0058974	Female	Tumour mass	ileocecum	adenocarcinoma	64	T4N2M0

B0060127	Male	Tumour mass	right hemicolon	adenocarcinoma	49	T2N0M0
B0060644	Female	Tumour mass	sigmoid colon	adenocarcinoma	65	T3N1cM0
B0062970	Male	Tumour mass	right hemicolon	adenocarcinoma	53	T4bN2bM1a
B0063214	Female	Tumour mass	ileocecum	adenocarcinoma	65	T2N2M1
B0063782	Female	Tumour mass	rectosigmoid	adenocarcinoma	51	T3N2M1
B0063918	Male	Tumour mass	sigmoid colon	adenocarcinoma	71	T4N0M0
B0066989	Female	Tumour mass	colon	adenocarcinoma	40	T3N0M0
B0027766	Female	Tumour mass	right hemicolon	adenocarcinoma	50	T4N1cM0
B0048402	Female	Tumour mass	colon	adenocarcinoma	37	T4N1M1
B0056543	Male	Tumour mass	rectum	adenocarcinoma	65	T4N0M0
B0055465	Male	Tumour mass	ileocecum	adenocarcinoma	54	T4N2M1
B0036557	Male	Tumour mass	rectum	adenocarcinoma	64	T4N2M0
B0061212	Female	Tumour mass	rectum	adenocarcinoma	43	T4bN2bM1a
B0059654	Female	Tumour mass	sigmoid colon	adenocarcinoma	55	T4N1M1
B0046554	Male	Tumour mass	colon	adenocarcinoma	68	T4N0M0
B0056554	Male	Tumour mass	colon	adenocarcinoma	72	T3N0M0
B0061223	Female	Tumour mass	sigmoid colon	adenocarcinoma	61	T4N1cM0
B0033454	Male	Tumour mass	left hemicolon	adenocarcinoma	50	T3N2M1
B0051221	Female	Tumour mass	right hemicolon	adenocarcinoma	44	T4N1M1
B0060201	Female	Tumour mass	colon	adenocarcinoma	65	T4N2M1
B0058548	Male	Tumour mass	ileocecum	adenocarcinoma	44	T4N1M1

Appendix Table S2. Clinical information for 11 colorectal cancer cohort; pre and post oxaliplatin treatment.

Patient No.	Sex	Collect procedure	origin of tissue	Sample pathology diagnosis	Age at diagnosis	TNM stage
B0033322	Male	biopsy	sigmoid colon	adenocarcinoma	57	T2N2aM1a
B0044089		Tumour mass	sigmoid colon	adenocarcinoma		T2N2aM1a
B0050898	Male	biopsy	rectum	adenocarcinoma	50	T4N1M1
B0053926		Tumour mass	rectum	adenocarcinoma		T4N1M1
B0058435	Male	Tumour mass	sigmoid colon	adenocarcinoma	56	T4N2M0
B0060820		Tumour mass	sigmoid colon	adenocarcinoma		T4N2M0
B0057998	Female	biopsy	rectum	adenocarcinoma	47	T3N1M0
B0060098		Tumour mass	rectum	adenocarcinoma		T3N1M0
B0057098	Male	biopsy	sigmoid colon	adenocarcinoma	52	T4N2M0
B0062139		Tumour mass	sigmoid colon	adenocarcinoma		T4N2M0
B0059033	Male	biopsy	sigmoid colon	adenocarcinoma	55	T4N1M1
B0061098		Tumour mass	sigmoid colon	adenocarcinoma		T4N1M1
B0049023	Female	biopsy	right hemicolon	adenocarcinoma	59	T2N1M1
B0060981		Tumour mass	right hemicolon	adenocarcinoma		T2N1M1
B0051232	Female	biopsy	rectum	adenocarcinoma	49	T4N2M1
B0059879		Tumour mass	rectum	adenocarcinoma		T4N2M1
B0053467	Female	biopsy	left hemicolon	adenocarcinoma	51	T2N2M0
B0061211		Tumour mass	left hemicolon	adenocarcinoma		T2N2M0
B0055376	Male	Tumour mass	sigmoid colon	adenocarcinoma	55	T3N1cM0
B0059008		Tumour mass	sigmoid colon	adenocarcinoma		T3N1cM0
B0048978	Female	biopsy	sigmoid colon	adenocarcinoma	61	T4N1M1
B0056721		Tumour mass	sigmoid colon	adenocarcinoma		T4N1M1

Appendix Table S3. Clinical information for colorectal cancer cohort; TMA.

Patient No.	Sex	Collect procedure	origin of tissue	Sample pathology diagnosis	Age at diagnosis	T stage	N stage	M stage	clinical stage
D15A3053	Male	Resection	sigmoid colon	mucinous adenocarcinoma	40	T4a	N2b	M0	3C
D15A3054	Female	Resection	colon	mucinous adenocarcinoma	59	T4a	N0	M0	2B
D15A3055	Male	Resection	right hemicolon	adenocarcinoma	71	T4a	N0	M0	2B
D15A3003	Male	Resection	right hemicolon	adenocarcinoma	82	T4b	N2b	M0	3C
D15A3030	Male	Resection	sigmoid colon	adenocarcinoma	59	T4a	N1	M0	3B
D15A3066	Male	Resection	colon	adenocarcinoma	62	T3	N0	M0	2A
D15A3067	Male	Resection	right hemicolon	adenocarcinoma	61	T4a	N2b	M0	3C
D15A3068	Male	Resection	sigmoid colon	adenocarcinoma	75	T4a	N1	M0	3B
D15A3069	Female	Resection	left hemicolon	adenocarcinoma	41	T3	N0	M0	2A
D15A3070	Male	Resection	colon	adenocarcinoma	77	T4a	N1	M0	3B
D15A3072	Female	Resection	sigmoid colon	adenocarcinoma	82	T4a	N0	M0	2B
D15A3083	Female	Resection	ileocecum	adenocarcinoma	80	T3	N1	M0	3B
D15A3086	Male	Resection	right hemicolon	adenocarcinoma	78	T4a	N0	M0	2B
D15A3092	Female	Resection	transverse colon	adenocarcinoma	66	T3	N0	M0	2A
D15A3015	Female	Resection	left hemicolon	mucinous adenocarcinoma	76	T3	N1	M0	3B
D15A3096	Male	Resection	sigmoid colon	adenocarcinoma	79	T4a	N1c	M0	3B
D15A3097	Female	Resection	right hemicolon	adenocarcinoma	59	T4a	N1	M1	4
D15A3098	Male	Resection	ascending colon	adenocarcinoma	52	T4a	N1	M0	3B
D15A3104	Female	Resection	right hemicolon	adenocarcinoma	64	T4a	N1	M0	3B
D15A3122	Female	Resection	right hemicolon	adenocarcinoma	43	T2	N0	M0	1
D15A3129	Female	Resection	right hemicolon	adenocarcinoma	78	T4a	N0	M0	2B
D15A3169	Male	Resection	sigmoid colon	mucinous adenocarcinoma	75	T3	N2b	M0	3C
D15A3170	Female	Resection	ascending colon	adenocarcinoma	63	T3	N0	M0	2A
D15A3171	Male	Resection	rectosigmoid	adenocarcinoma	54	T3	N1	M0	3B
D15A3172	Female	Resection	colon	adenocarcinoma	61	T4a	N0	M0	2B
D15A3173	Male	Resection	sigmoid colon	adenocarcinoma	57	T4a	N0	M0	2B
D15A3167	Male	Resection	rectosigmoid	adenocarcinoma	64	T3	N0	M0	2A
D15A3168	Male	Resection	ascending colon	adenocarcinoma	84	T3	N0	M0	2A
D15A3177	Male	Resection	sigmoid colon	adenocarcinoma	49	T4a	N1	M0	3B

D15A3179	Male	Resection	sigmoid colon	adenocarcinoma	45	T3	N0	M0	2A
D15A3181	Male	Resection	sigmoid colon	adenocarcinoma	57	T3	N0	M0	2A
D15A3182	Male	Resection	splenic region of the colon	adenocarcinoma	77	T3	N1	M0	3B
D15A3191	Female	Resection	Hepatic region of colon	adenocarcinoma	80	T3	N1	M0	3B
D15A3193	Male	Resection	sigmoid colon	adenocarcinoma	76	T4a	N0	M0	2B
D15A3132	Male	Resection	colon	adenocarcinoma	54		N0	M0	
D15A3195	Male	Resection	right hemicolon	adenocarcinoma	79	T3	N1	M0	3B
D15A3196	Female	Resection	sigmoid colon	adenocarcinoma	87	T4a	N1	M0	3B
D15A3197	Male	Resection	Hepatic region of colon	mucinous adenocarcinoma	34	T3	N1	M0	3B
D15A3198	Female	Resection	transverse colon	adenocarcinoma	68	T3	N0	M0	2A
D15A3200	Female	Resection	rectosigmoid	adenocarcinoma	57	T3	N0	M0	2A
D15A3203	Female	Resection	sigmoid colon	adenocarcinoma	73	T3	N0	M0	2A
D15A3209	Female	Resection	descending colon	adenocarcinoma	83	T4a		M0	
D15A3210	Male	Resection	sigmoid colon	adenocarcinoma	84	T3	N0	M0	2A
D15A3212	Female	Resection	right hemicolon	adenocarcinoma	75	T4a	N2b	M1	4
D15A3223	Male	Resection	sigmoid colon	adenocarcinoma	76	T3	N1	M0	3B
D15A3134	Female	Resection	right hemicolon	mucinous adenocarcinoma	80	T3	N0	M0	2A
D15A3225	Female	Resection	splenic region of the colon	adenocarcinoma	81	T3	N1	M0	3B
D15A3227	Female	Resection	sigmoid colon	adenocarcinoma	74	T4b	N0	M0	2C
D15A3228	Male	Resection	Hepatic region of colon	adenocarcinoma	78	T3	N0	M0	2A
D15A3135	Male	Resection	splenic region of the colon	adenocarcinoma	71	T4a	N0	M0	2B
D15A3230	Male	Resection	sigmoid colon	adenocarcinoma	55	T3	N0	M0	2A
D15A3231	Male	Resection	transverse colon	adenocarcinoma	54		N1	M0	3
D15A3138	Male	Resection	right hemicolon	adenocarcinoma	57	T4a	N1	M0	3B
D15A3139	Female	Resection	right hemicolon	adenocarcinoma	76	T4a	N2b	M0	3C
D15A3221	Female	Resection	ascending colon	adenocarcinoma	71	T3	N1	M0	3B
D15A3222	Male	Resection	sigmoid colon	adenocarcinoma	80	T3	N0	M0	2A
D15A3233	Female	Resection	transverse colon	adenocarcinoma	53	T3	N2a	M0	3B
D15A3141	Male	Resection	sigmoid colon	adenocarcinoma	70		N1	M0	3
D15A3234	Female	Resection	right hemicolon	adenocarcinoma	84	T3	N0	M0	2A
D15A3236	Female	Resection	ascending colon	adenocarcinoma	67	T2	N0	M0	1

D15A3237	Female	Resection	descending colon	adenocarcinoma	87	T3	N1	M0	3B
D15A3238	Female	Resection	sigmoid colon	adenocarcinoma	67	T3	N0	M0	2A
D15A3283	Male	Resection	sigmoid colon	adenocarcinoma	56	T4a	N0	M0	2B
D15A3284	Male	Resection	ascending colon	adenocarcinoma	54	T3	N0	M0	2A
D15A3285	Female	Resection	ascending colon	mucinous adenocarcinoma	56	T4b	N1	M0	3C
D15A3286	Male	Resection	sigmoid colon	adenocarcinoma	74	T3	N1	M0	3B
D15A3287	Female	Resection	Hepatic region of colon	adenocarcinoma	76	T4a	N1	M0	3B
D15A3289	Female	Resection	descending colon	adenocarcinoma	54	T4a	N2a	M0	3C
D15A3290	Male	Resection	rectosigmoid	adenocarcinoma	67	T3	N0	M0	2A
D15A3291	Male	Resection	sigmoid colon	adenocarcinoma	62	T3	N0	M0	2A
D15A3292	Female	Resection	sigmoid colon	adenocarcinoma	61	T3	N1	M0	3B
D15A3293	Female	Resection	sigmoid colon	adenocarcinoma	55	T3	N1	M0	3B
D15A3296	Male	Resection	descending colon	adenocarcinoma	73	T3	N0	M0	2A
D15A3281	Male	Resection	ascending colon	adenocarcinoma	63	T3	N0	M0	2A
D15A3297	Female	Resection	splenic region of the colon	adenocarcinoma	72	T2	N0	M0	1
D15A3298	Male	Resection	ascending colon	adenocarcinoma	70	T3	N0	M0	2A
D15A3299	Male	Resection	sigmoid colon	adenocarcinoma	58	T3	N0	M0	2A
D15A3312	Male	Resection	ascending colon	adenocarcinoma	70	T3	N0	M0	2A
D15A3313	Female	Resection	ascending colon	adenocarcinoma	72	T3	N0	M0	2A
D15A3319	Female	Resection	sigmoid colon	adenocarcinoma	77	T3	N0	M0	2A
D15A3320	Male	Resection	Hepatic region of colon	adenocarcinoma	67	T3	N0	M0	2A
D15A3321	Male	Resection	Hepatic region of colon	adenocarcinoma	49	T3	N0	M0	2A
D15A3322	Male	Resection	ascending colon	adenocarcinoma	61	T4b	N0	M0	2C
D15A3323	Male	Resection	left hemicolon	adenocarcinoma	81	T4a	N0	M0	2B
D15A3059	Male	Resection	colon	mucinous adenocarcinoma	60	T4a	N0	M0	2B
D15A3075	Female	Resection	sigmoid colon	adenocarcinoma	37	T4a	N2b	M1	4
D15A3077	Male	Resection	sigmoid colon	adenocarcinoma	65	T3	N0	M0	2A
D15A3091	Female	Resection	sigmoid colon	adenocarcinoma	75	T3	N2a	M0	3B
D15A3095	Female	Resection	right hemicolon	adenocarcinoma	55	T4a	N0	M0	2B
D15A3131	Male	Resection	right hemicolon	adenocarcinoma	19	T4b	N0	M0	2C
D15A3192	Female	Resection	right hemicolon	adenocarcinoma	49	T4b	N2a	M0	3C
D15A3194	Male	Resection	right hemicolon	adenocarcinoma	47	T3	N0	M0	2A

D15A3205	Male	Resection	right hemicolon	adenocarcinoma	69	T4a	N0	M0	2B
D15A3136	Female	Resection	transverse colon	adenocarcinoma	53	T4a	N1	M0	3B
D15A3140	Female	Resection	right hemicolon	adenocarcinoma	61	T3	N0	M0	2A
D15A3314	Female	Resection	Hepatic region of colon	adenocarcinoma	82	T4a	N0	M0	2B

Appendix Table S4

Association between TXNIP expression and clinicopathological features of patients with colorectal cancer in the cohort of 42 CRC patients

	Total (n = 42)	TXNIP expression Low (n = 21)	High (n = 21)	*P* value
Gender				0.5366
Male	22	12	10	
Female	20	9	11	
Age(year)				>0.9999
<65	32	16	16	
≥65	10	5	5	
T stage				>0.9999
T1-T2	9	4	5	
T3-T4	33	17	16	
N stage				0.0219*
N0	14	3	11	
N1+N2+N3	28	18	10	
M stage				0.0278*
M0	25	9	16	
M1	17	12	5	
Clinical stage				0.0219*
I/II	14	3	11	
III/IV	28	18	10	

*$P<0.05$

The TNM staging system stands for Tumor, Node, Metastasis. T describes the size of the primary tumor (T1-2; <5cm. T3-4; >5cm). N describes the presence of tumor cells in the lymph nodes (N0; no lymph nodes. N1-3 >0). M describes whether there are any observable metastases (M0; no metastases. M1; metastases). The clinical stage system is as follows: I/II; the tumor has remained stable or grown, but hasn't spread. III/IV; the tumor has spread, either locally (III) or systemically (IV).

Appendix Table S5

Association between TXNIP expression and clinicopathological features of patients with colorectal cancer in the TMA cohort

	Total	TXNIP expression		
		Low	High	
	(n = 96)	(n = 48)	(n = 48)	*P* value
Gender				>0.9999
Male	52	26	26	
Female	44	22	22	
Age(year)				0.2191
<65	44	25	19	
≥65	52	23	29	
T stage				0.2419
T1-T2	3	0	3	
T3-T4	90	46	44	
N stage				<0.0001 *
N0	55	16	39	
N1+N2+N3	40	31	9	
M stage				0.2421
M0	93	45	48	
M1	3	3	0	
Clinical stage				<0.0001 *
I/II	54	16	38	
III/IV	40	31	9	

*$P<0.05$

The TNM staging system stands for Tumor, Node, Metastasis. T describes the size of the primary tumor (T1-2; <5cm. T3-4; >5cm). N describes the presence of tumor cells in the lymph nodes (N0; no lymph nodes. N1-3 >0). M describes whether there are any observable metastases (M0; no metastases. M1; metastases). The clinical stage system is as follows: I/II; the tumor has remained stable or grown, but hasn't spread. III/IV; the tumor has spread, either locally (III) or systemically (IV).

Appendix Table S6

Association between GDF15 expression and clinicopathological features of patients with colorectal cancer in the cohort of 42 CRC patients

		GDF15 expression		
	Total (n = 42)	Low (n = 21)	High (n = 21)	*P* value
Gender				>0.9999
Male	22	11	11	
Female	20	10	10	
Age(year)				0.7171
<65	32	15	17	
≥65	10	6	4	
T stage				0.4520
T1-T2	9	6	3	
T3-T4	33	15	18	
N stage				0.0219*
N0	14	11	3	
N1+N2+N3	28	10	18	
M stage				0.0017*
M0	25	18	7	
M1	17	3	14	
Clinical stage				0.0219*
I/II	14	11	3	
III/IV	28	10	18	

$^{*}P<0.05$

The TNM staging system stands for Tumor, Node, Metastasis. T describes the size of the primary tumor (T1-2; <5cm. T3-4; >5cm). N describes the presence of tumor cells in the lymph nodes (N0; no lymph nodes. N1-3 >0). M describes whether there are any observable metastases (M0; no metastases. M1; metastases). The clinical stage system is as follows: I/II; the tumor has remained stable or grown, but hasn't spread. III/IV; the tumor has spread, either locally (III) or systemically (IV).

Appendix Table S7

Association between GDF15 expression and clinicopathological features of patients with colorectal cancer in TMA cohort

	Total	GDF15 expression		
		Low	High	
	(n = 96)	(n = 48)	(n = 48)	*P* value
Gender				
Male	52	27	25	0.6820
Female	44	21	23	
Age(year)				
<65	44	22	22	>0.9999
≥65	52	26	26	
T stage				
T1-T2	3	3	0	0.2419
T3-T4	90	44	46	
N stage				
N0	55	41	14	<0.0001*
N1+N2+N3	40	7	33	
M stage				
M0	93	48	45	0.2421
M1	3	0	3	
Clinical stage				
I/II	54	40	13	<0.0001*
III/IV	40	7	33	

*$P<0.05$

The TNM staging system stands for Tumor, Node, Metastasis. T describes the size of the primary tumor (T1-2; <5cm. T3-4; >5cm). N describes the presence of tumor cells in the lymph nodes (N0; no lymph nodes. N1-3 >0). M describes whether there are any observable metastases (M0; no metastases. M1; metastases). The clinical stage system is as follows: I/II; the tumor has remained stable or grown, but hasn't spread. III/IV; the tumor has spread, either locally (III) or systemically (IV).
