# Supplementary figures and images for: The MondoA-dependent TXNIP/GDF15 axis predicts oxaliplatin response in colorectal adenocarcinomas
Source: EMBO Mol Med. 2024 Aug 5;16(9):7. doi: 10.1038/s44321-024-00105-2 (PMC11393413; doi:10.1038/s44321-024-00105-2)

Figure 1F: pre-treatment

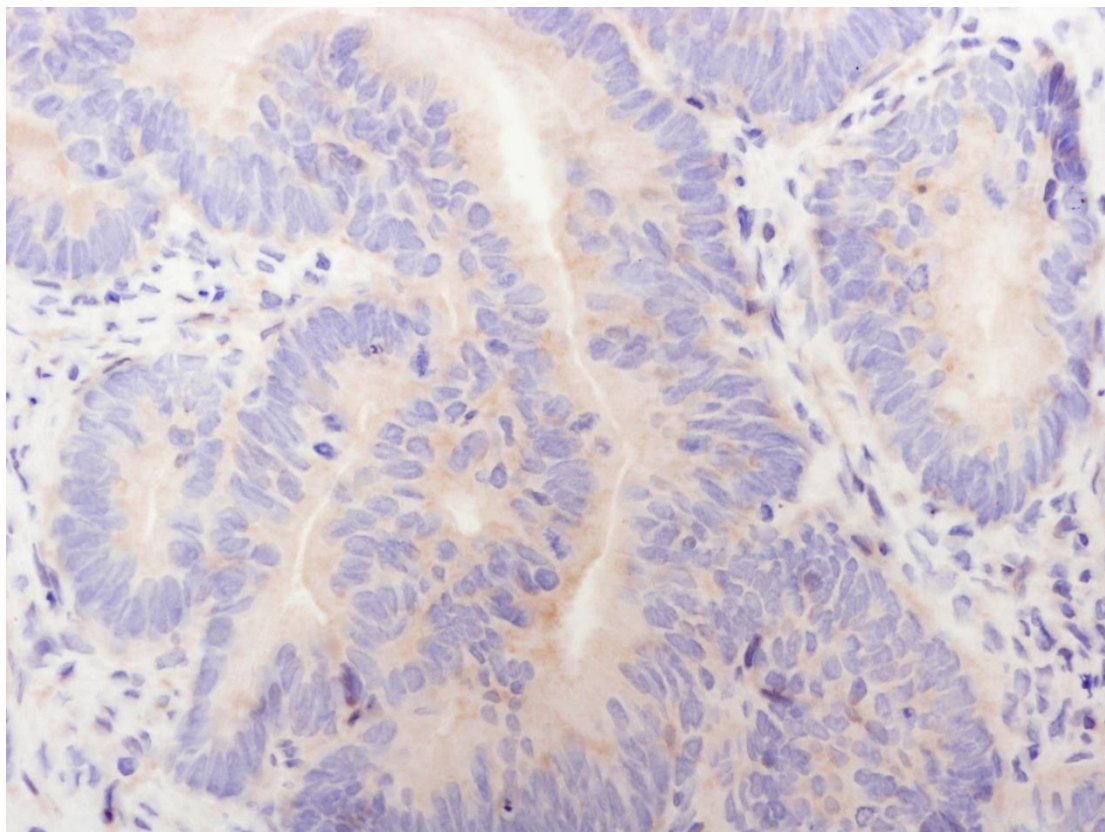

Figure 1F: post-treatment

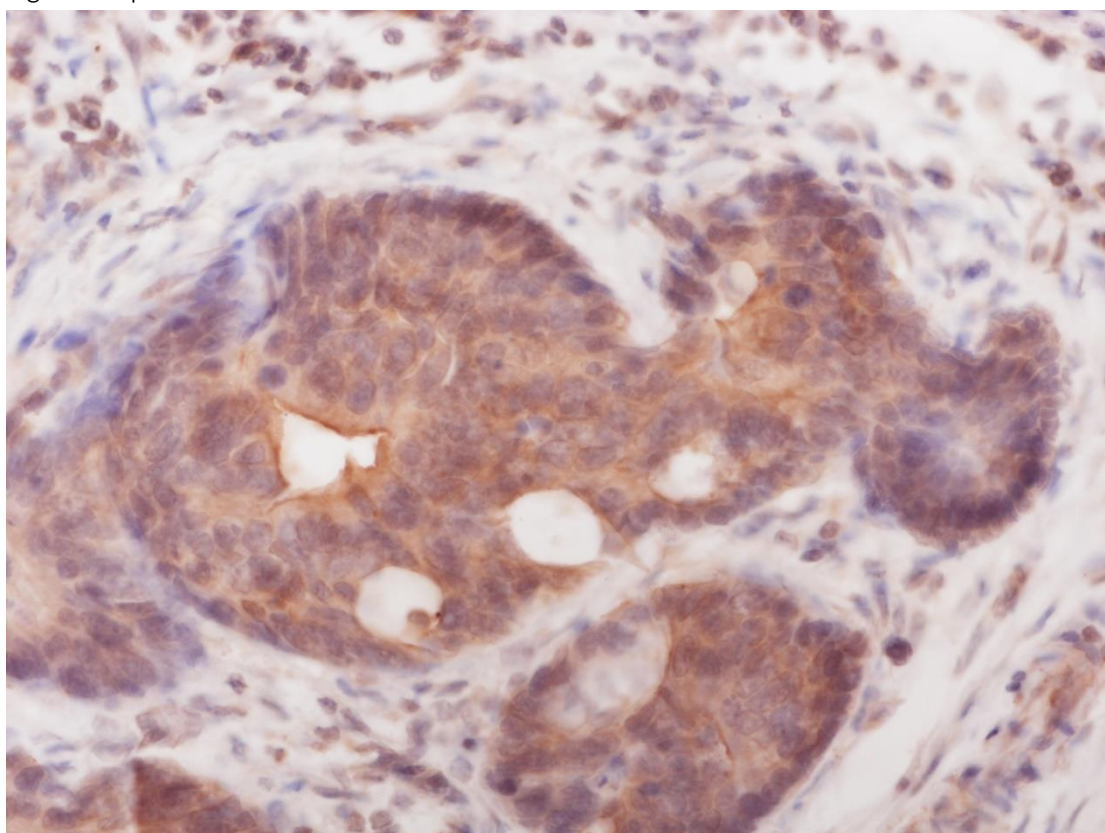

Supplement: Supplementary file 9 — Source data Fig. 1 [file 44321_2024_105_MOESM9_ESM.zip › Figure 1/Figure 1F/Figure 1F.pdf]

Figure 1A: ANT H&E

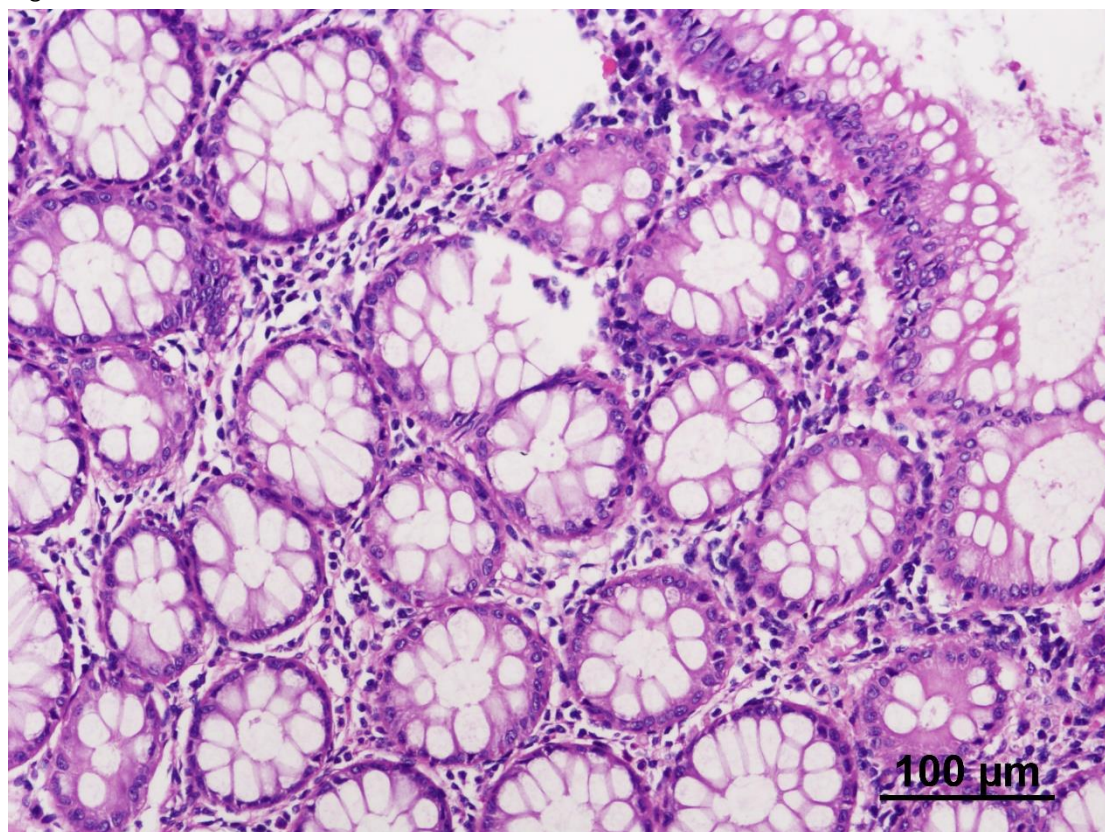

Figure 1A: Tumor H&E

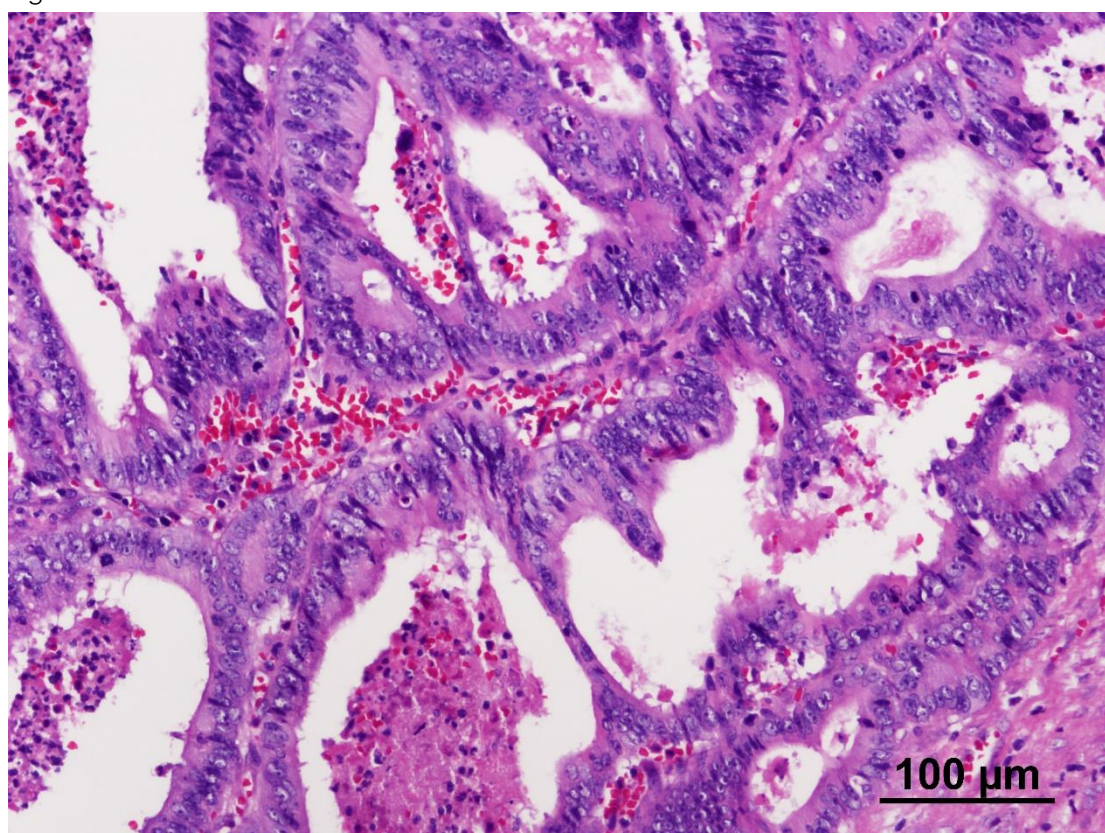

Figure 1A: ANT TXNIP

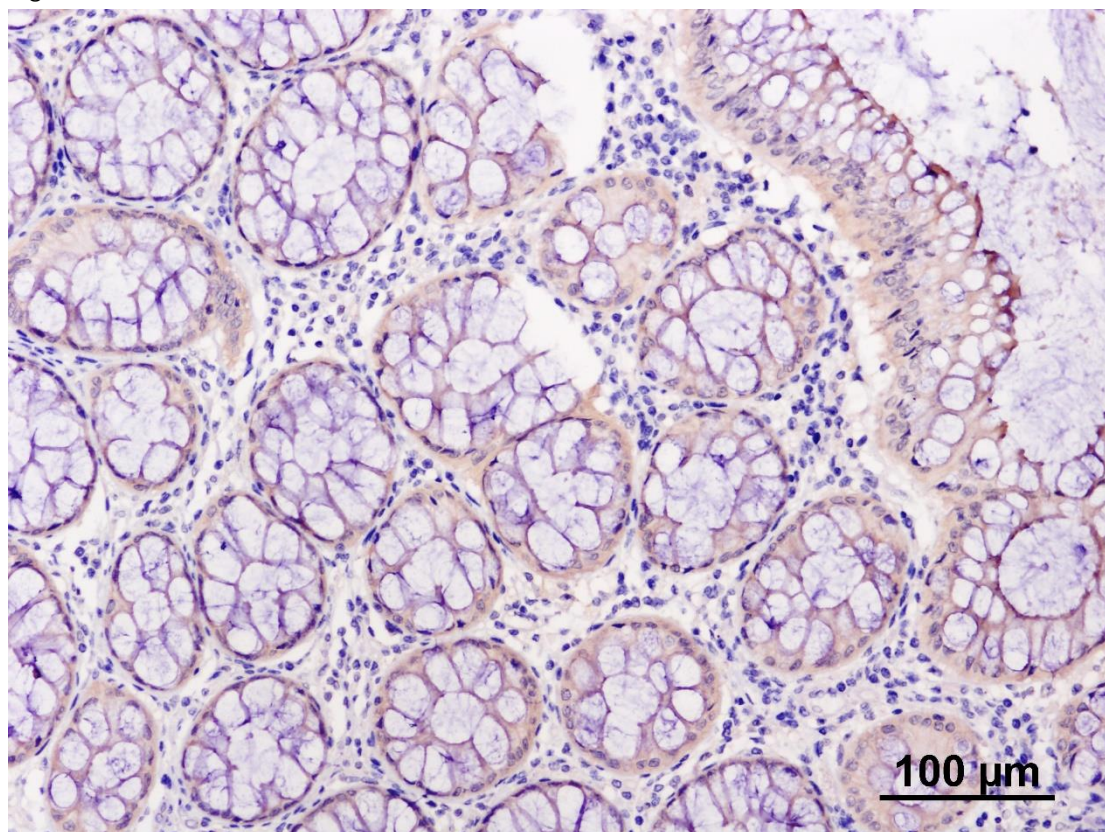

Figure 1A: Tumor TXNIP

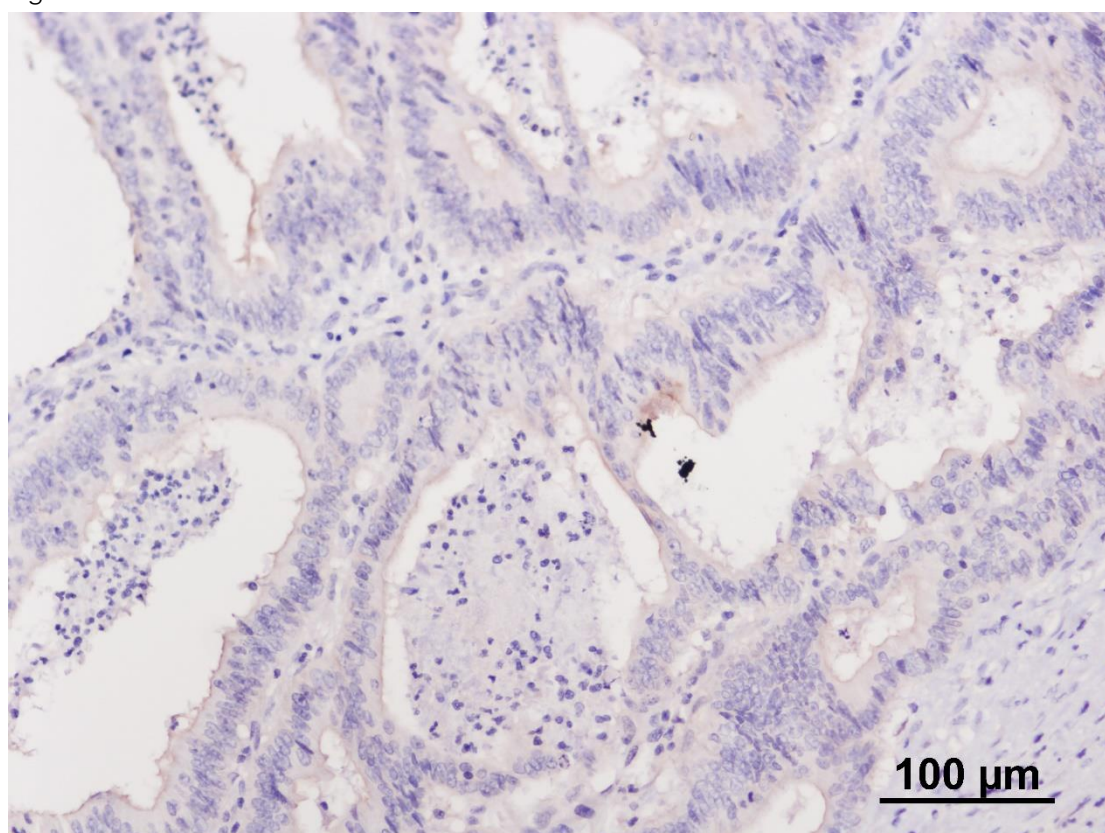

Supplement: Supplementary file 9 — Source data Fig. 1 [file 44321_2024_105_MOESM9_ESM.zip › Figure 1/Figure 1A/Figure 1A.pdf]

Figure 1D

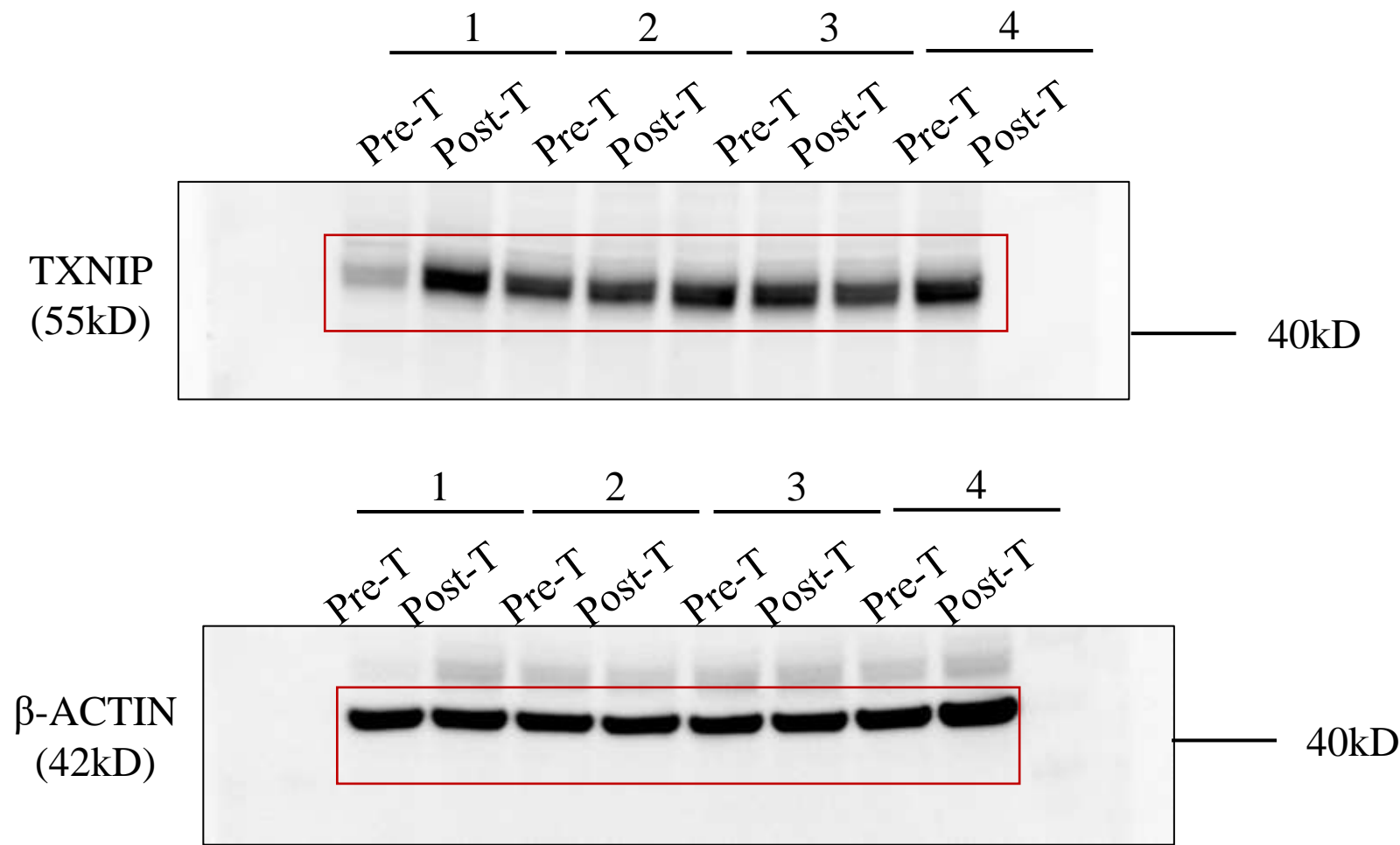

Figure 1D

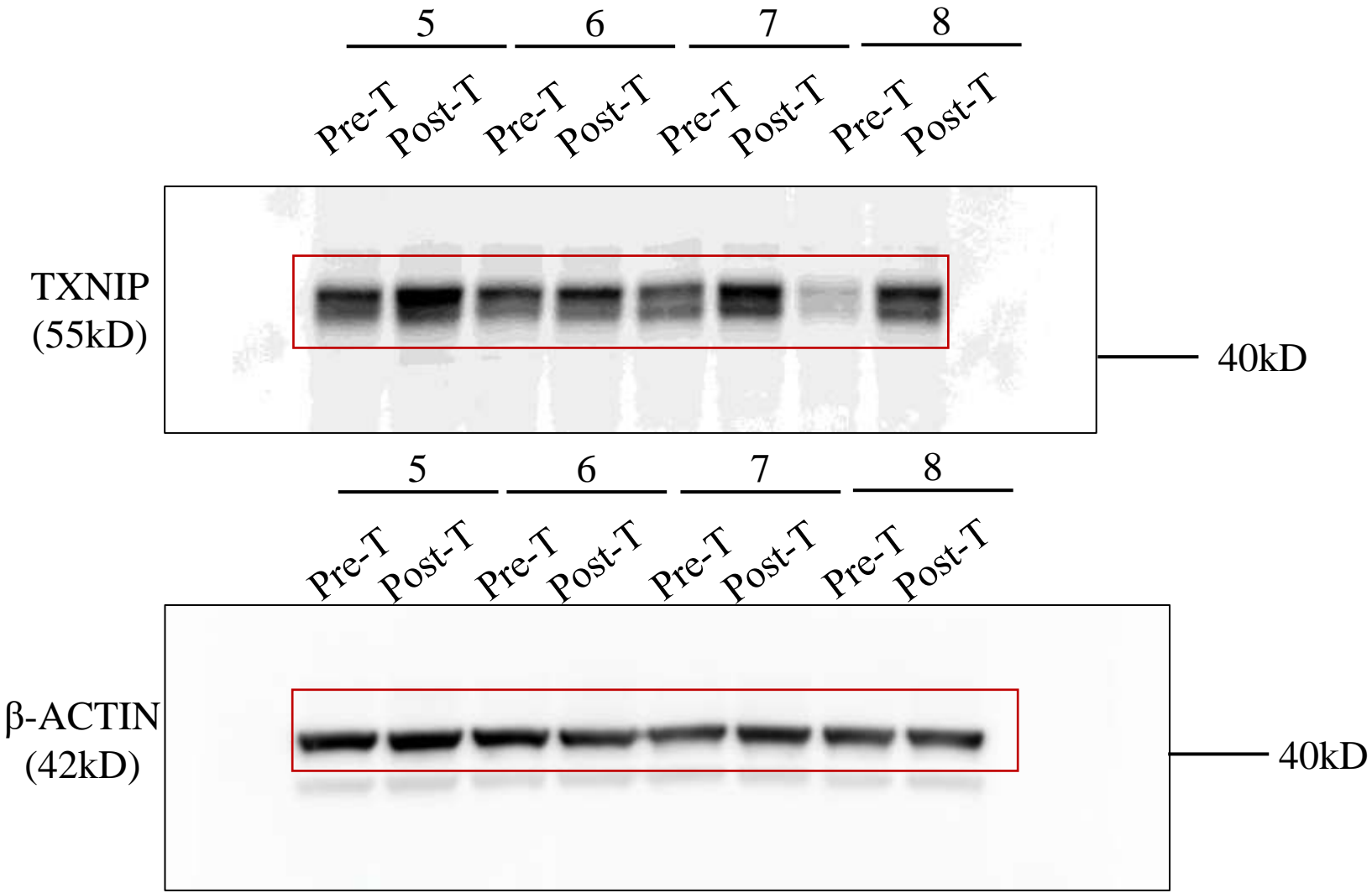

Figure 1D

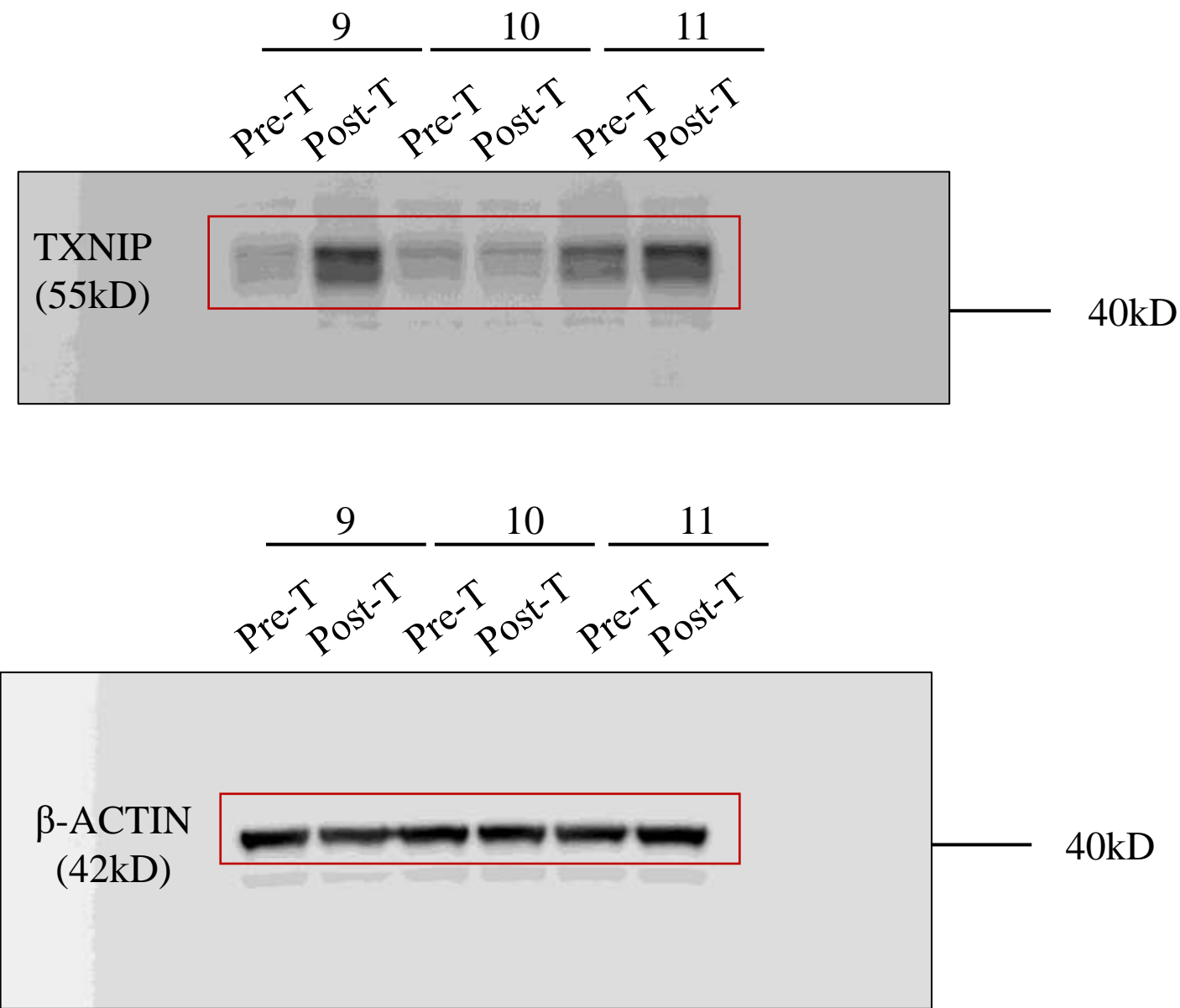

Supplement: Supplementary file 9 — Source data Fig. 1 [file 44321_2024_105_MOESM9_ESM.zip › Figure 1/Figure 1D/Figure 1D.pdf]

Figure 2G

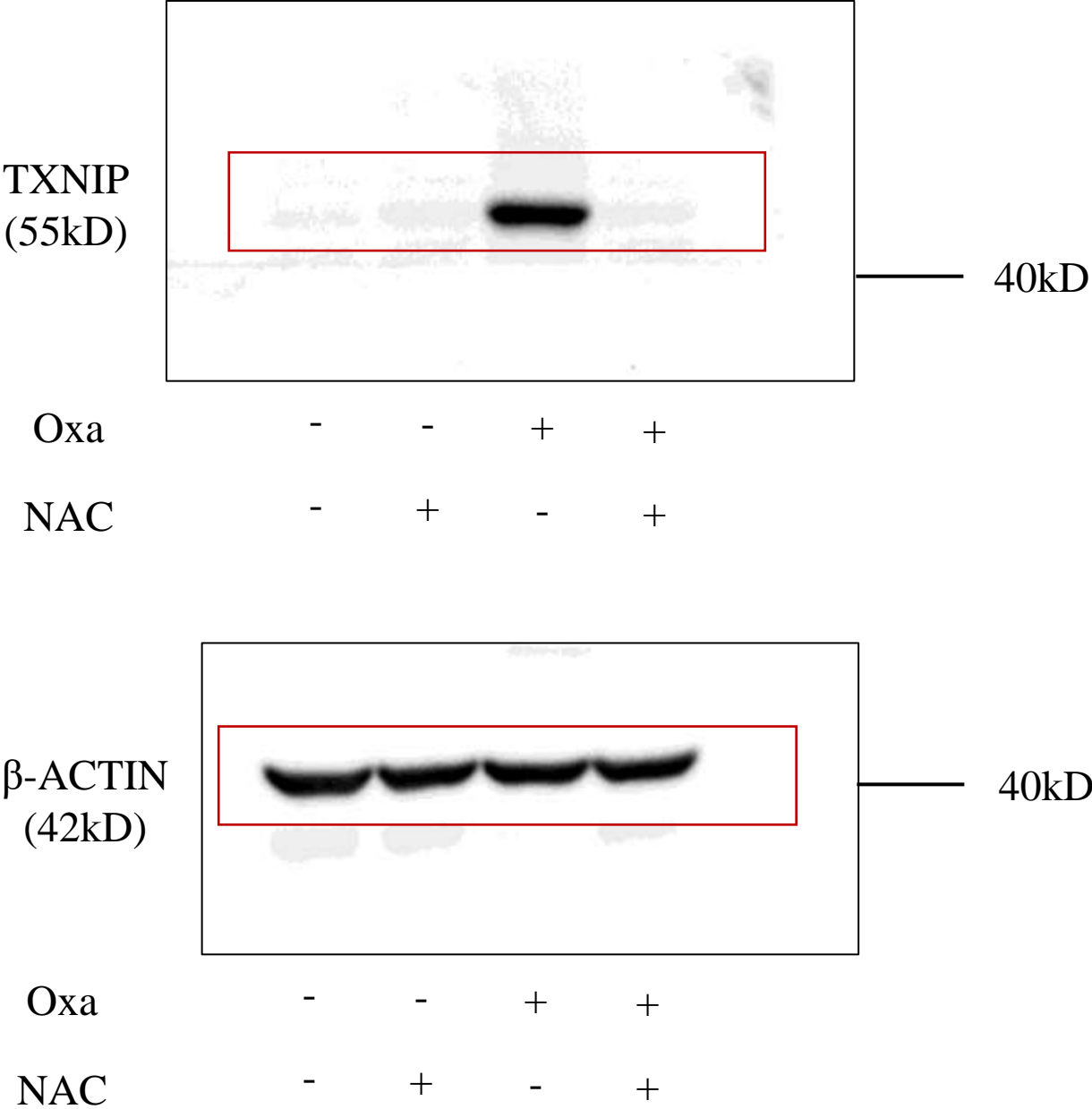

Supplement: Supplementary file 10 — Source data Fig. 2 [file 44321_2024_105_MOESM10_ESM.zip › Figure 2/Figure 2G/Figure 2G.pdf]

Figure 2H

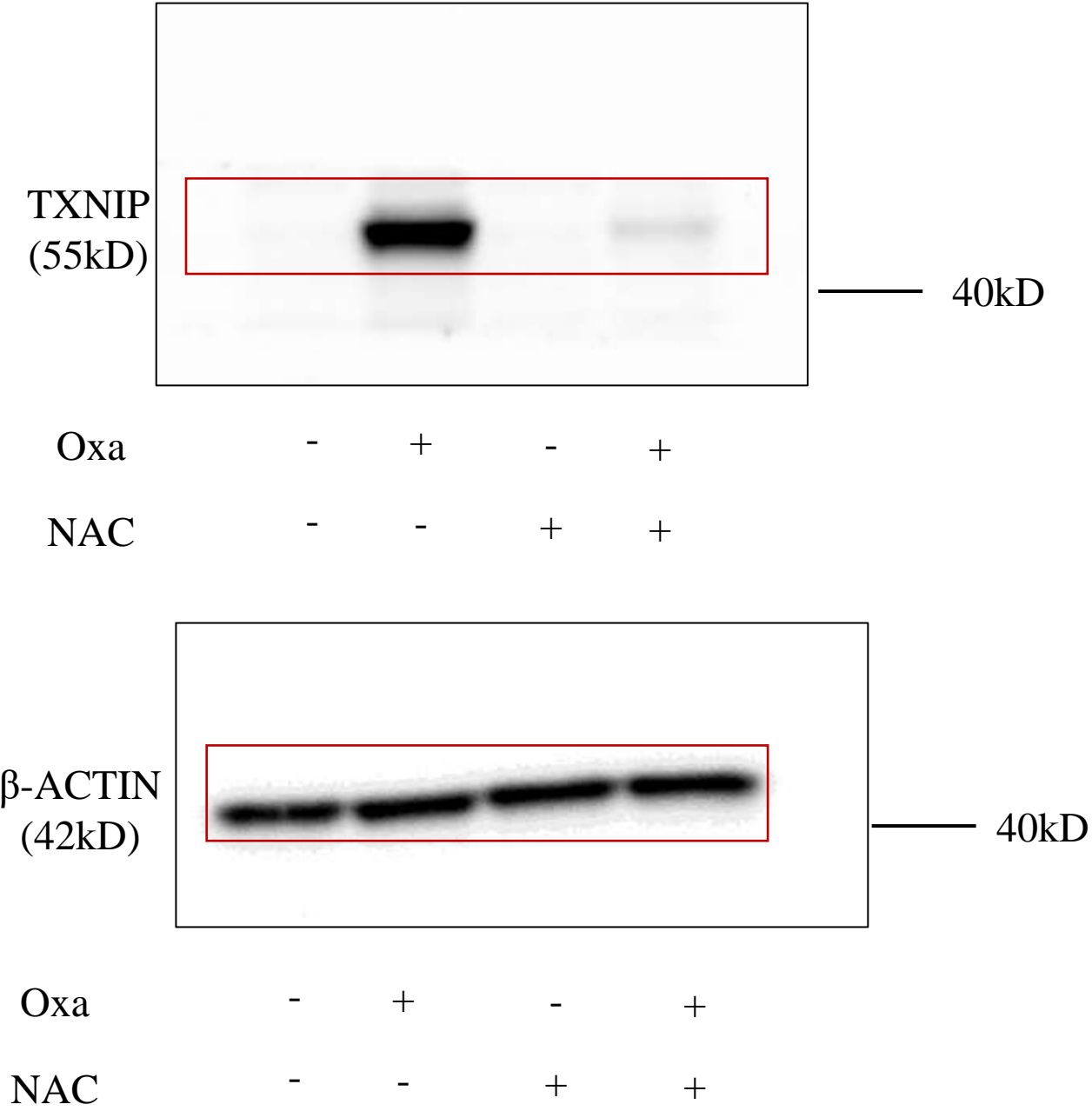

Supplement: Supplementary file 10 — Source data Fig. 2 [file 44321_2024_105_MOESM10_ESM.zip › Figure 2/Figure 2H/Figure 2H.pdf]

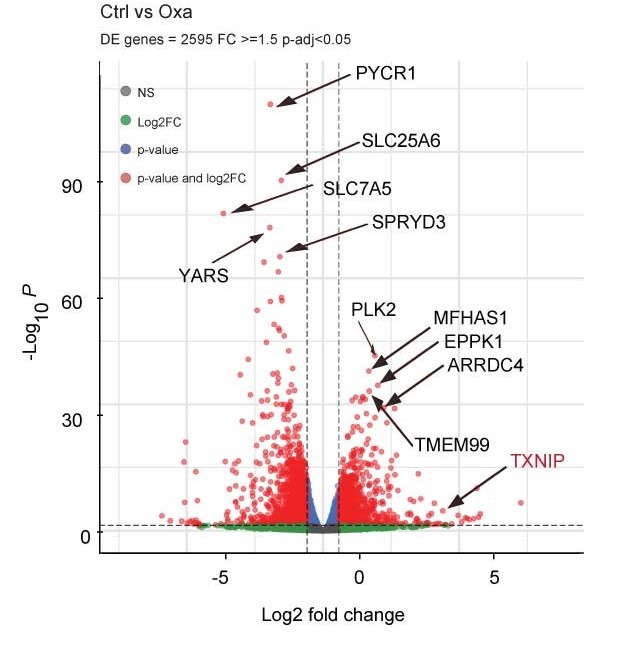

Supplement: Supplementary file 10 — Source data Fig. 2 [file 44321_2024_105_MOESM10_ESM.zip › Figure 2/Figure 2A/Figure 2A.jpg]

**Figure 2F**

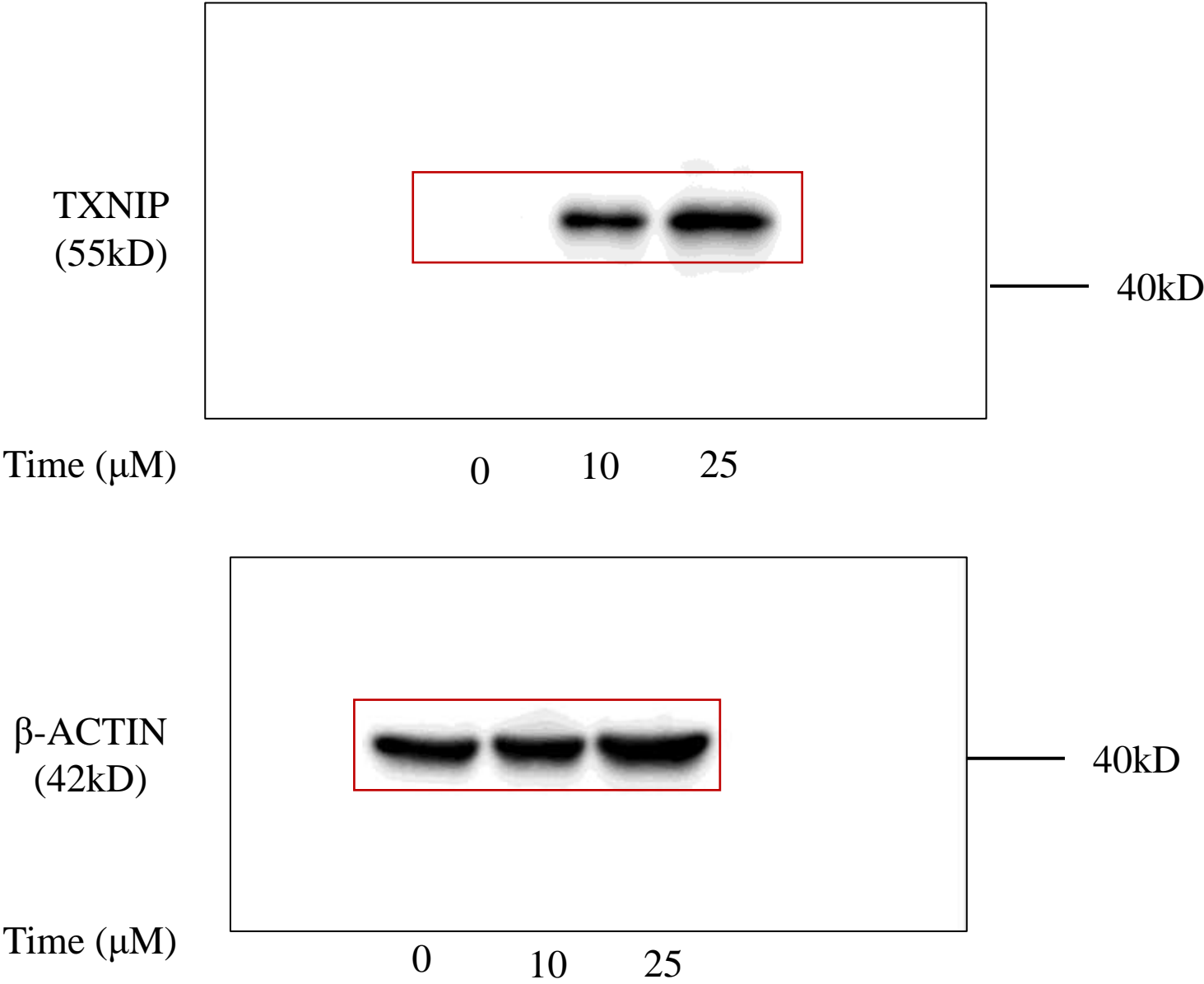

Supplement: Supplementary file 10 — Source data Fig. 2 [file 44321_2024_105_MOESM10_ESM.zip › Figure 2/Figure 2F/Figure 2F.pdf]

**Figure 2C**

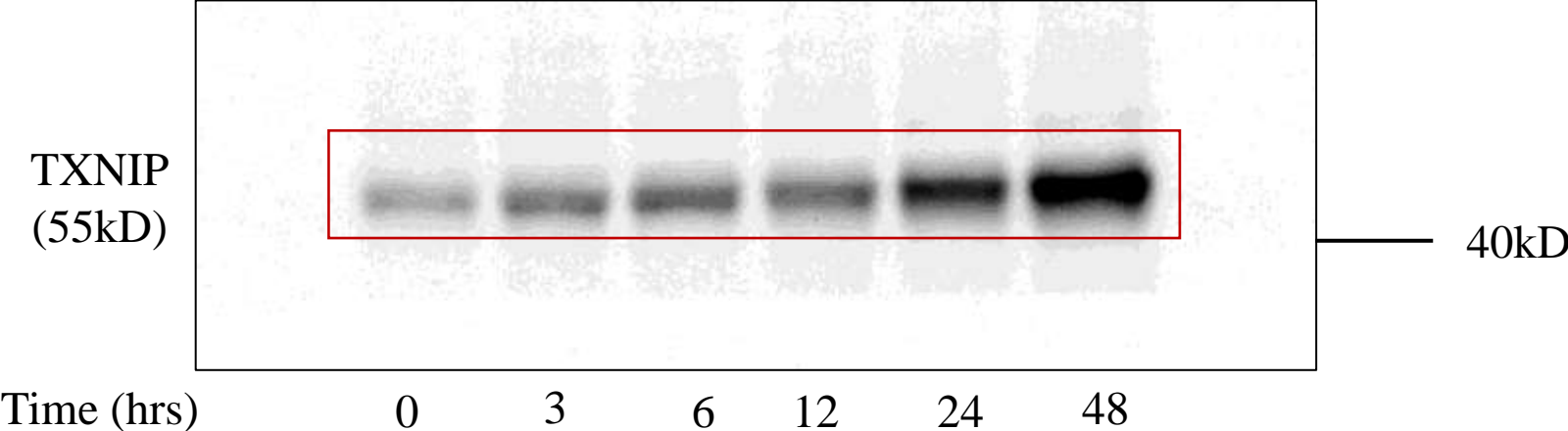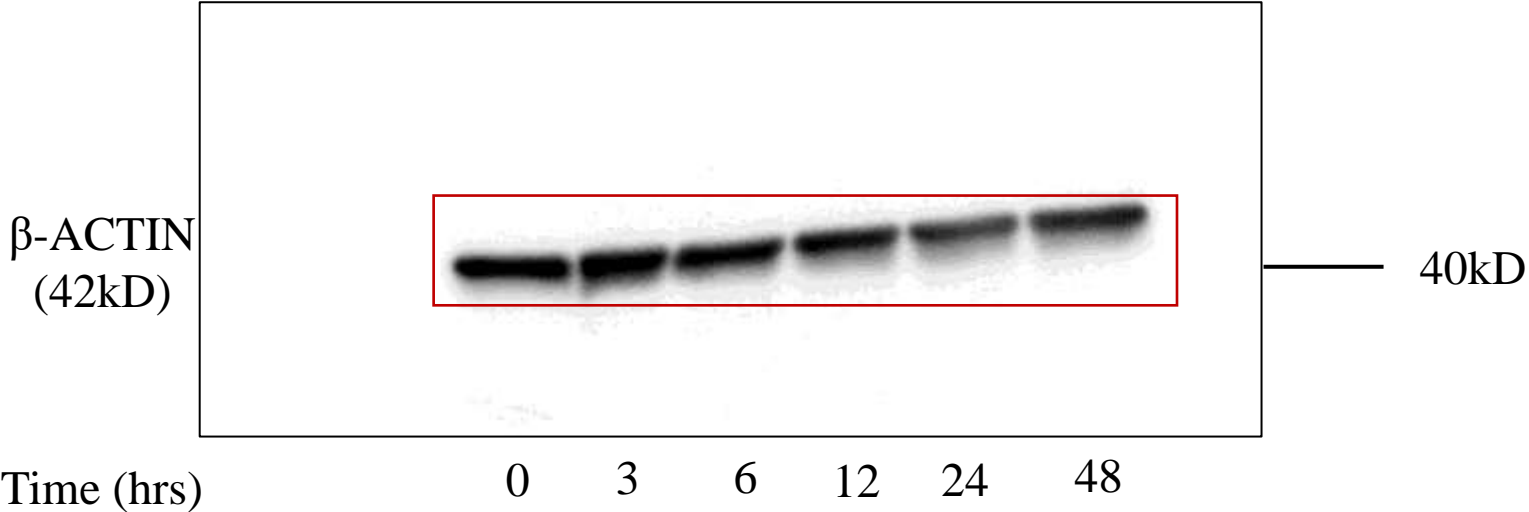

Supplement: Supplementary file 10 — Source data Fig. 2 [file 44321_2024_105_MOESM10_ESM.zip › Figure 2/Figure 2C/Figure 2C.pdf]

**Figure 2D**

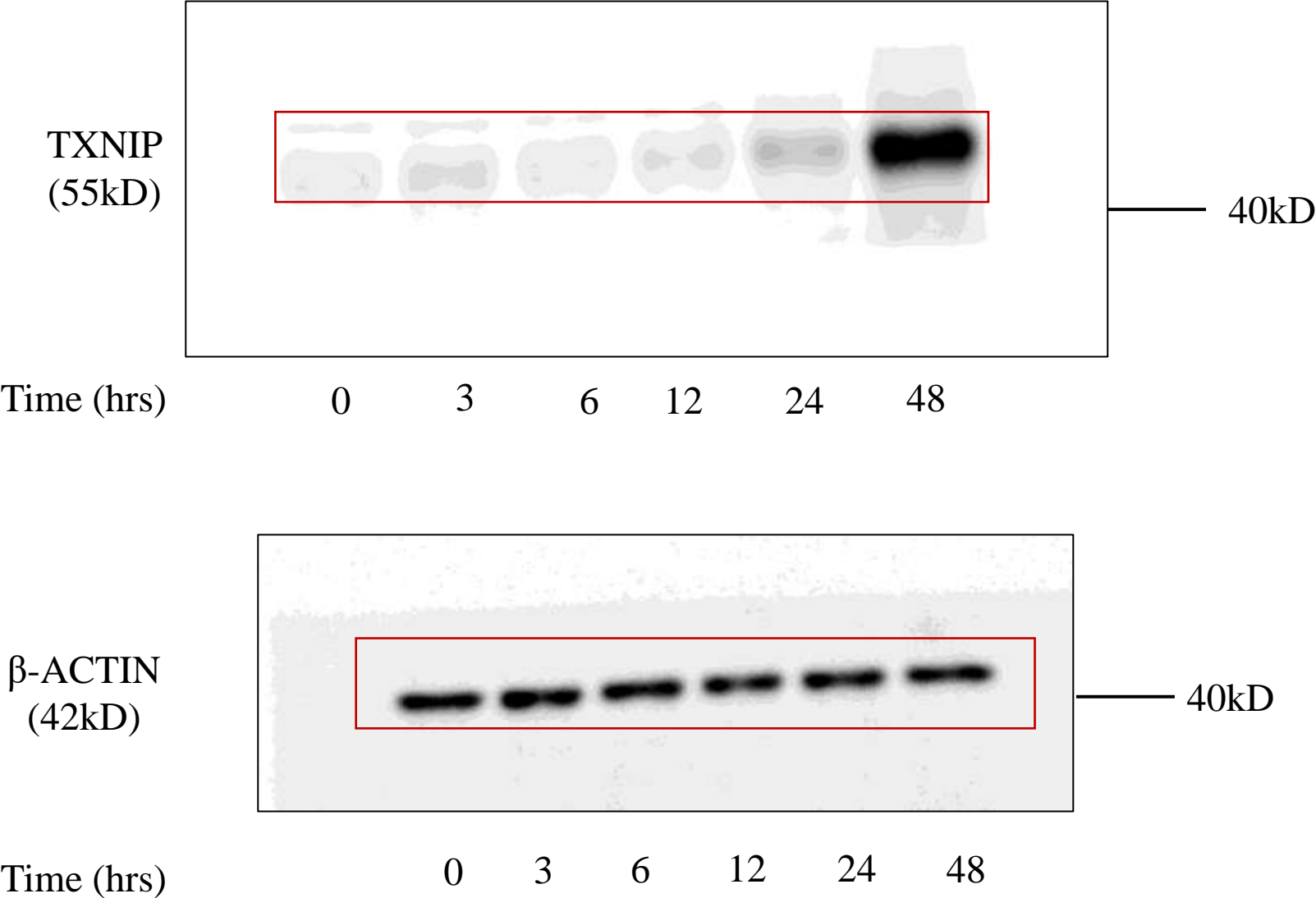

Supplement: Supplementary file 10 — Source data Fig. 2 [file 44321_2024_105_MOESM10_ESM.zip › Figure 2/Figure 2D/Figure 2D.pdf]

**Figure 2K**

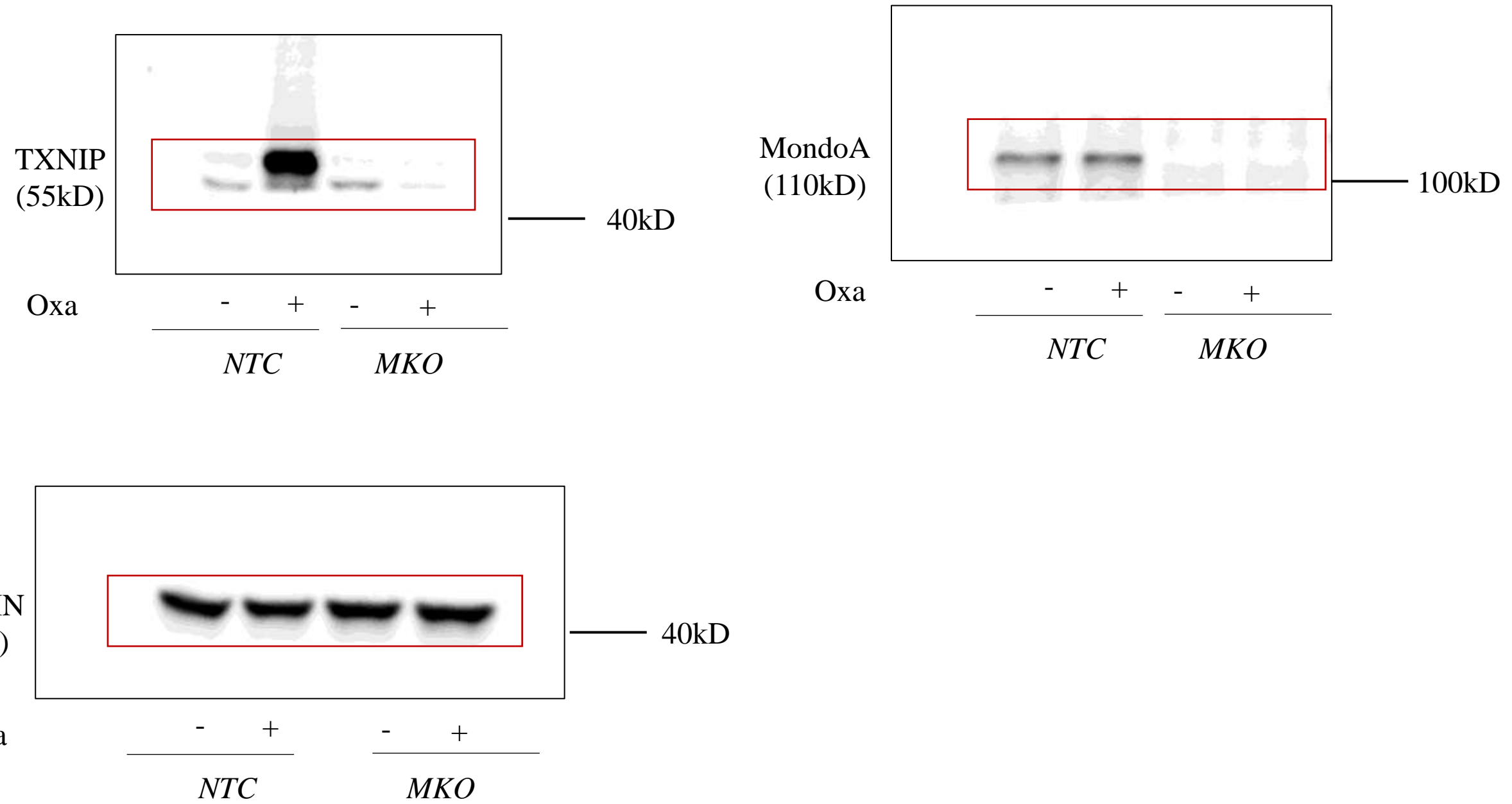

Supplement: Supplementary file 10 — Source data Fig. 2 [file 44321_2024_105_MOESM10_ESM.zip › Figure 2/Figure 2K/Figure 2K.pdf]

**Figure 2L**

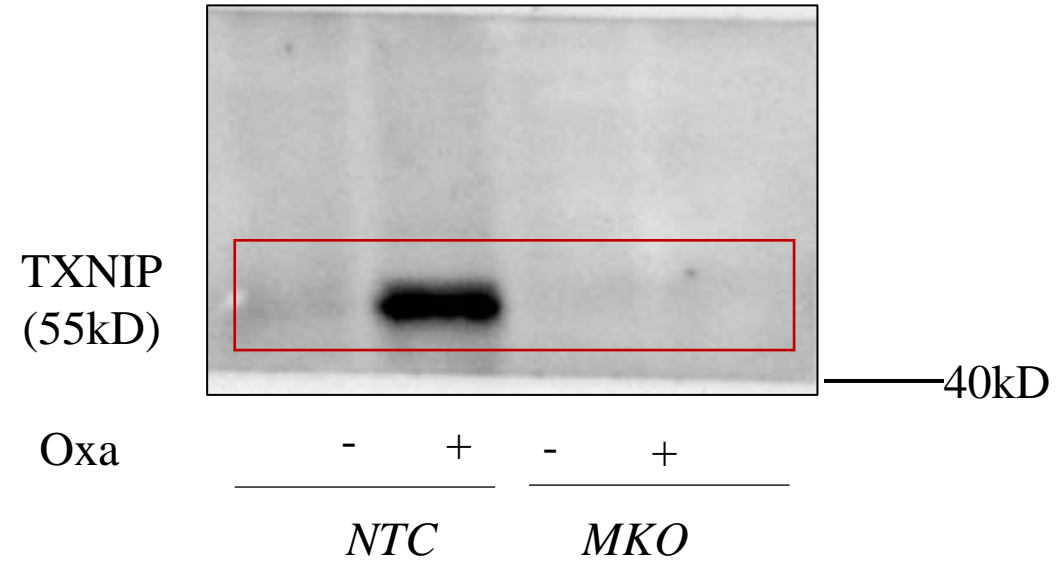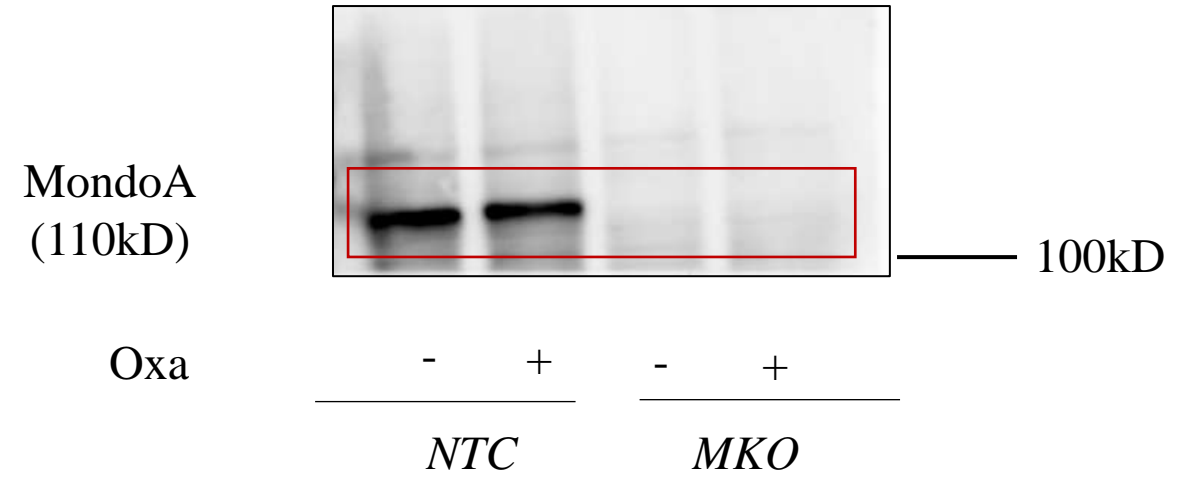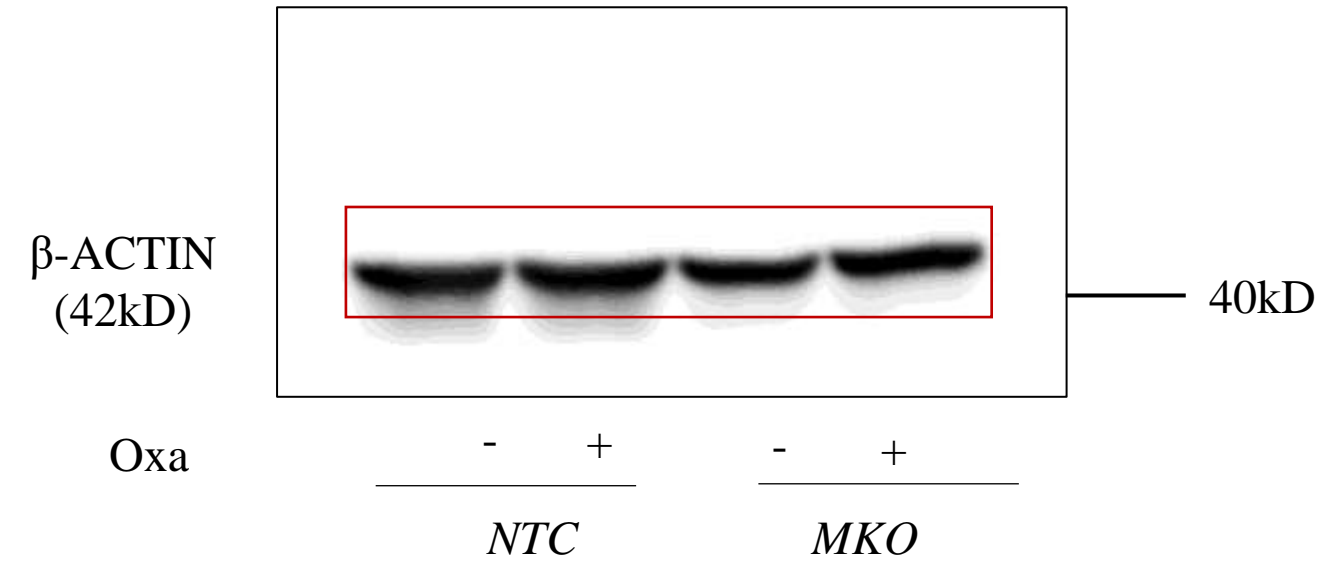

Supplement: Supplementary file 10 — Source data Fig. 2 [file 44321_2024_105_MOESM10_ESM.zip › Figure 2/Figure 2L/Figure 2L.pdf]

**Figure 2E**

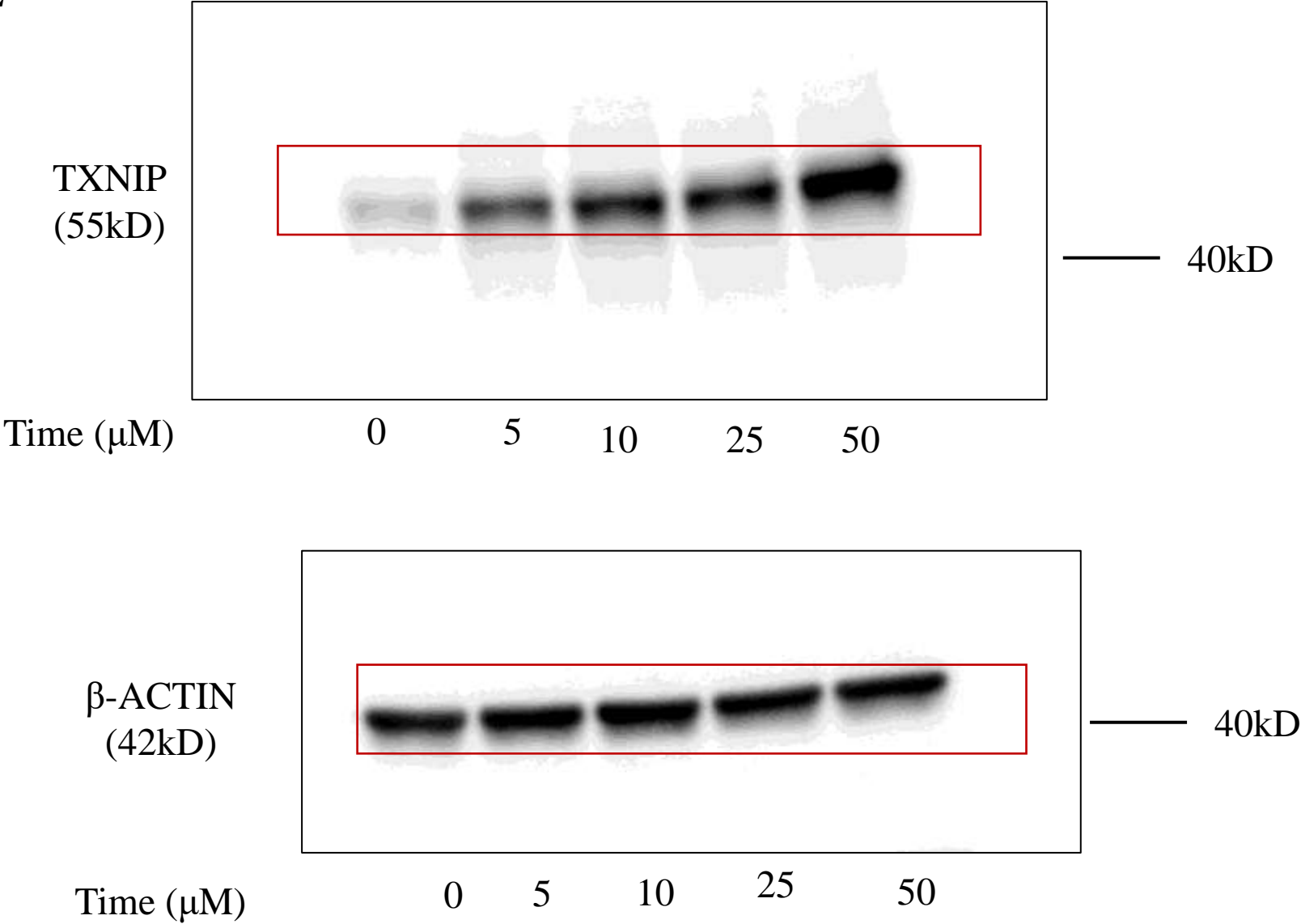

Supplement: Supplementary file 10 — Source data Fig. 2 [file 44321_2024_105_MOESM10_ESM.zip › Figure 2/Figure 2E/Figure 2E.pdf]

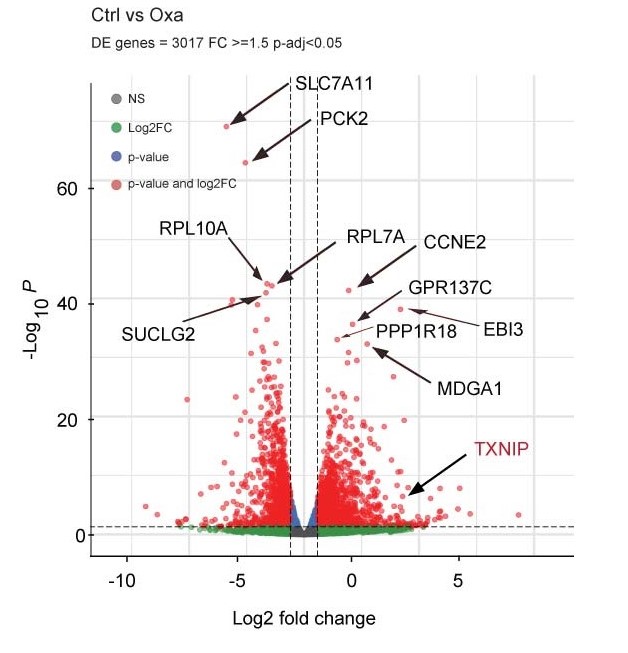

Supplement: Supplementary file 10 — Source data Fig. 2 [file 44321_2024_105_MOESM10_ESM.zip › Figure 2/Figure 2B/Figure 2B.jpg]

**NTC\_Ctrl**

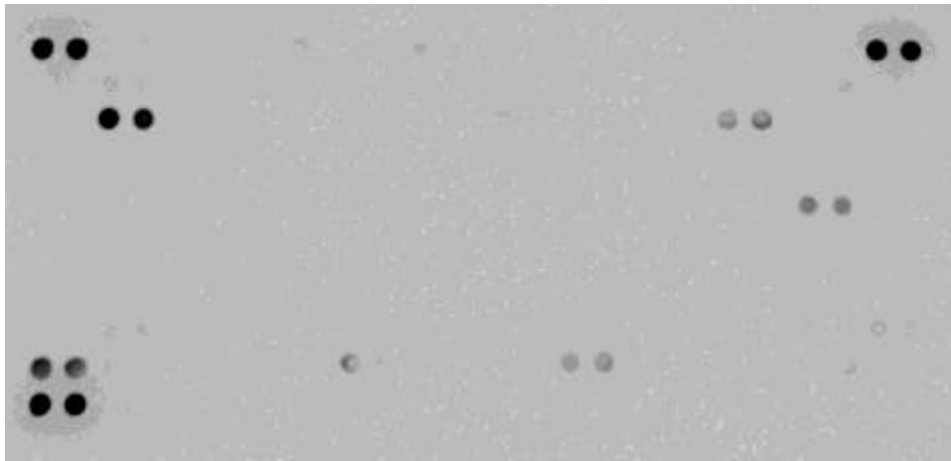

**TKO\_Ctrl**

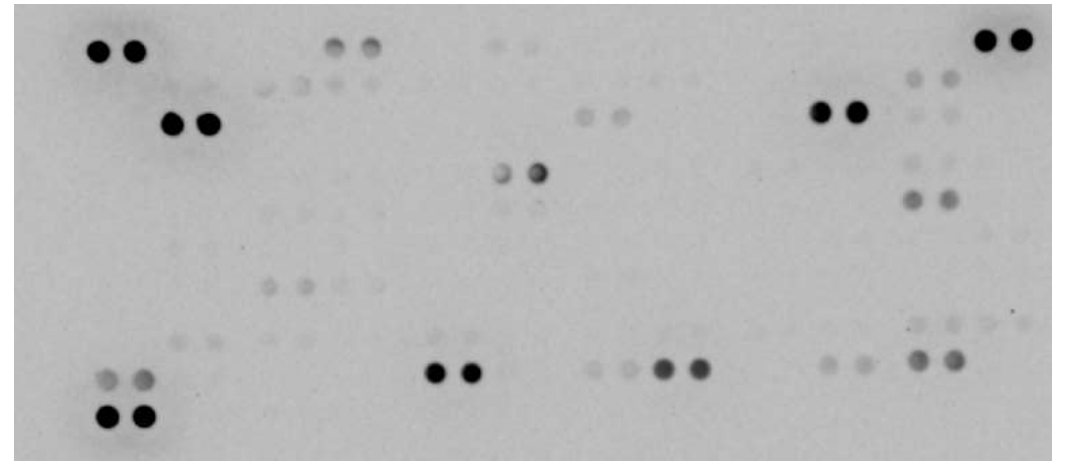

**NTC\_Oxa**

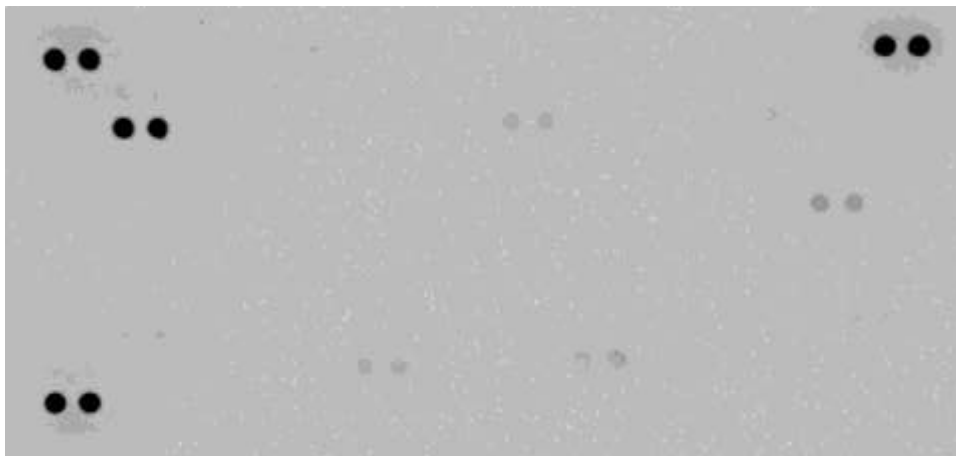

**TKO\_Oxa**

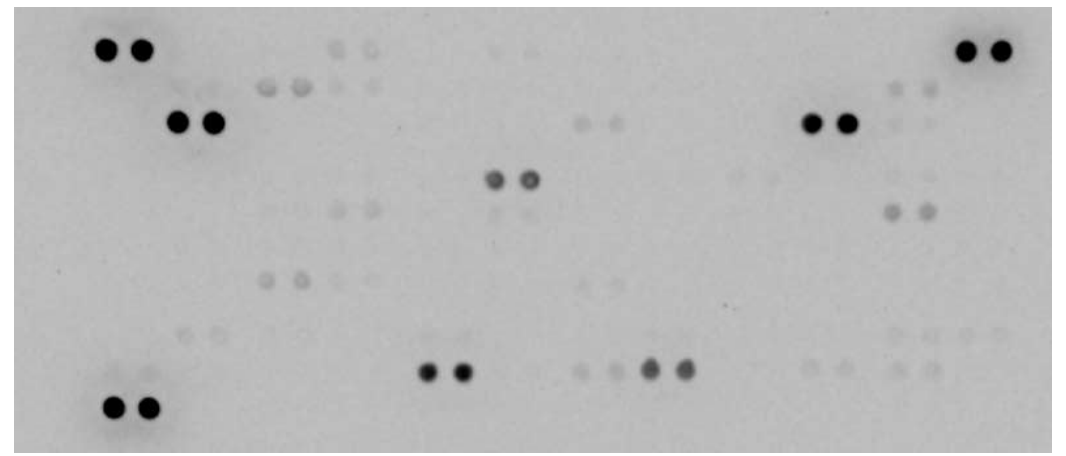

Supplement: Supplementary file 11 — Source data Fig. 3 [file 44321_2024_105_MOESM11_ESM.zip › Figure 3/Figure 3B/Figure 3B.pdf]

**Figure 3C**

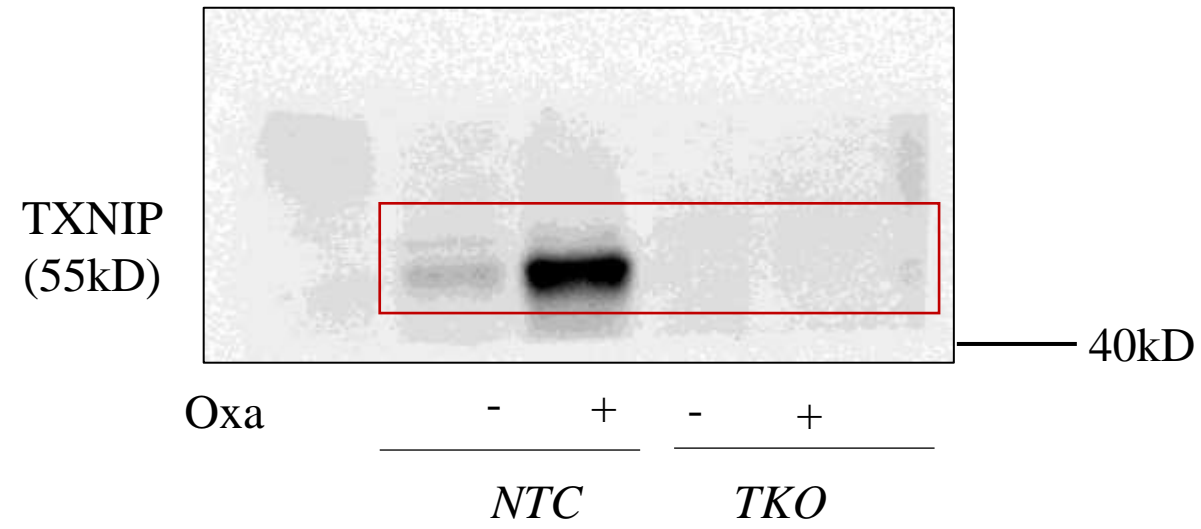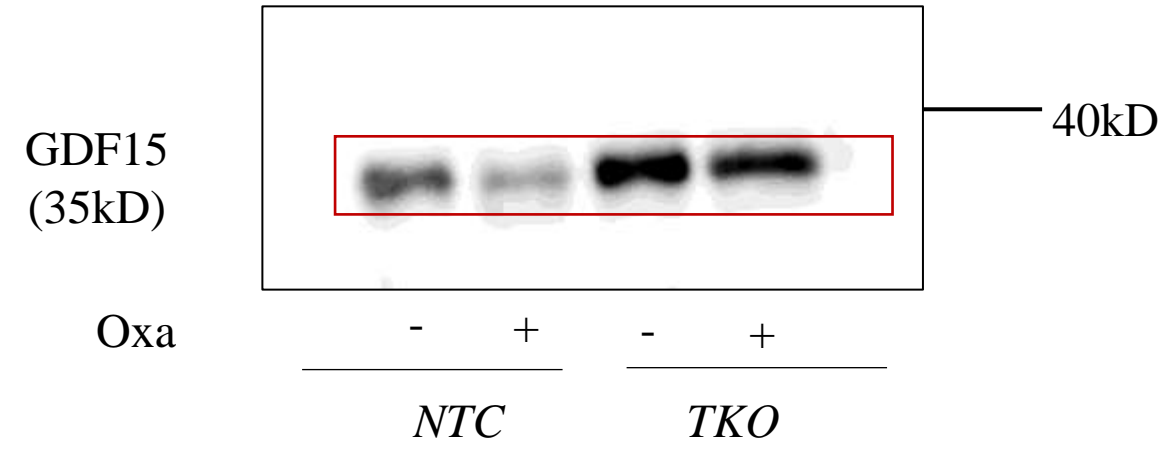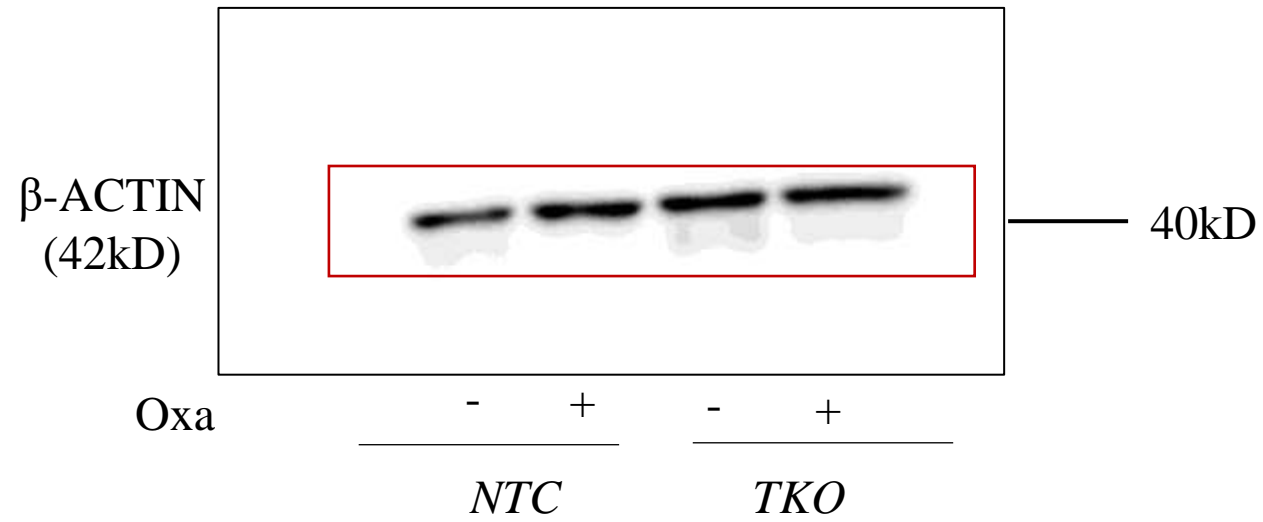

Supplement: Supplementary file 11 — Source data Fig. 3 [file 44321_2024_105_MOESM11_ESM.zip › Figure 3/Figure 3C/Figure 3C.pdf]

**Figure 3D**

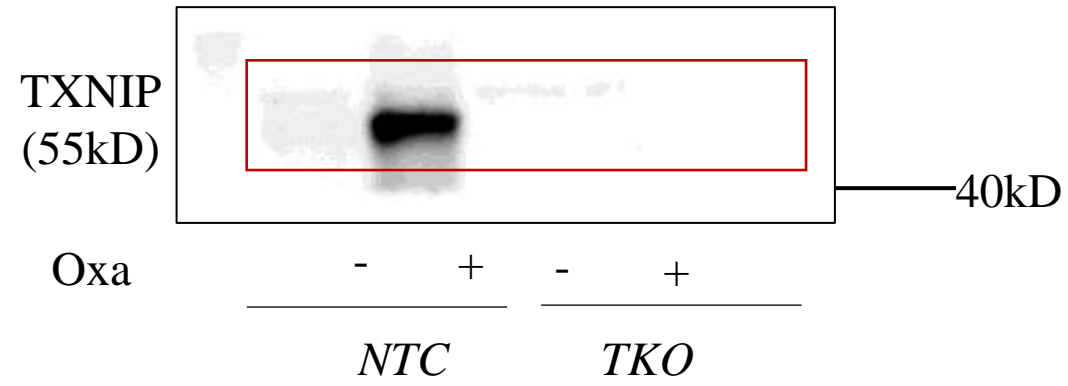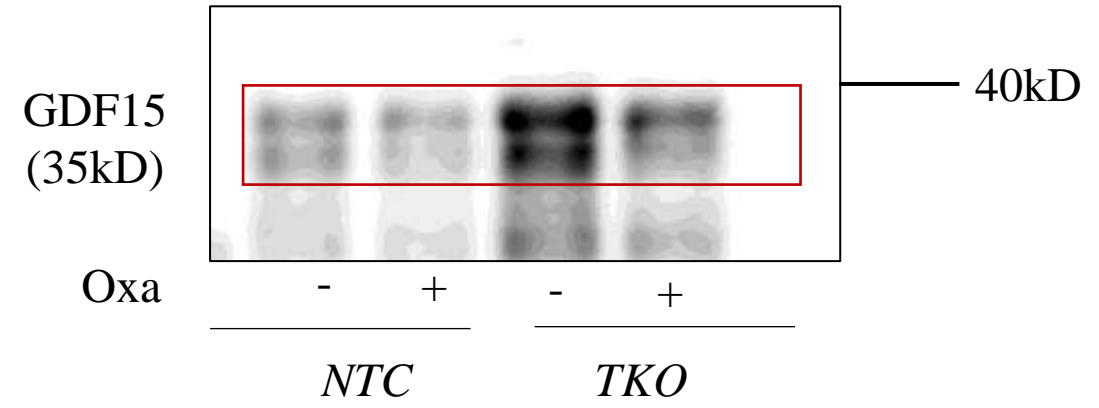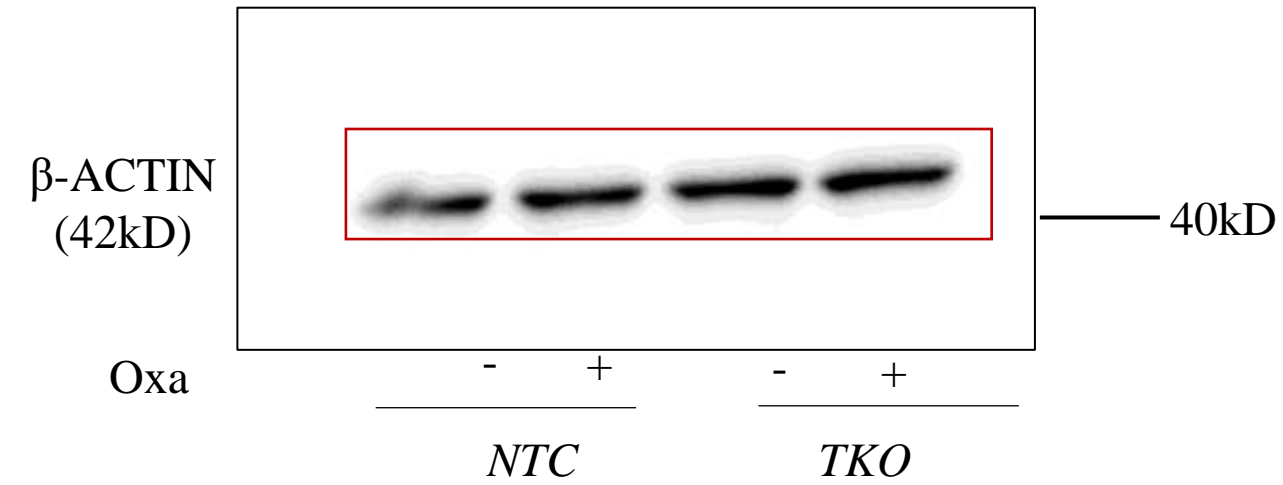

Supplement: Supplementary file 11 — Source data Fig. 3 [file 44321_2024_105_MOESM11_ESM.zip › Figure 3/Figure 3D/Figure 3D.pdf]

**Figure 3J**

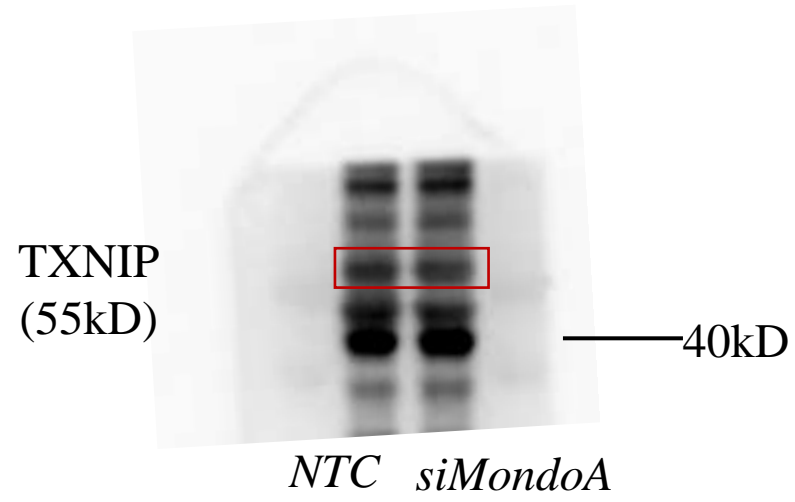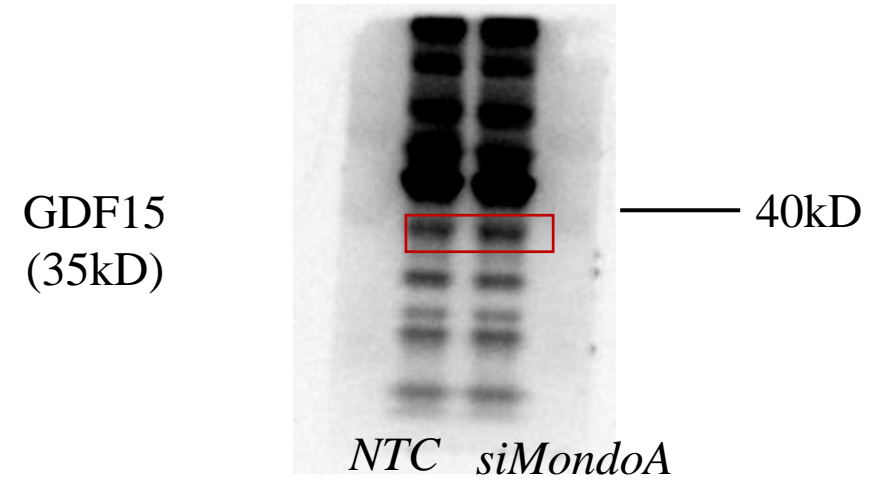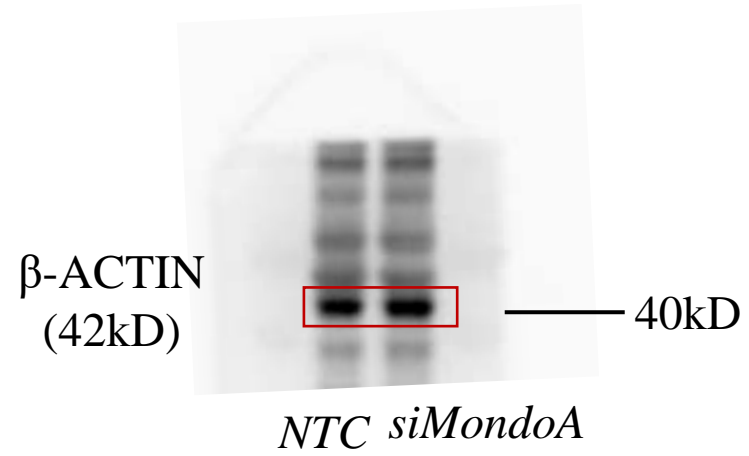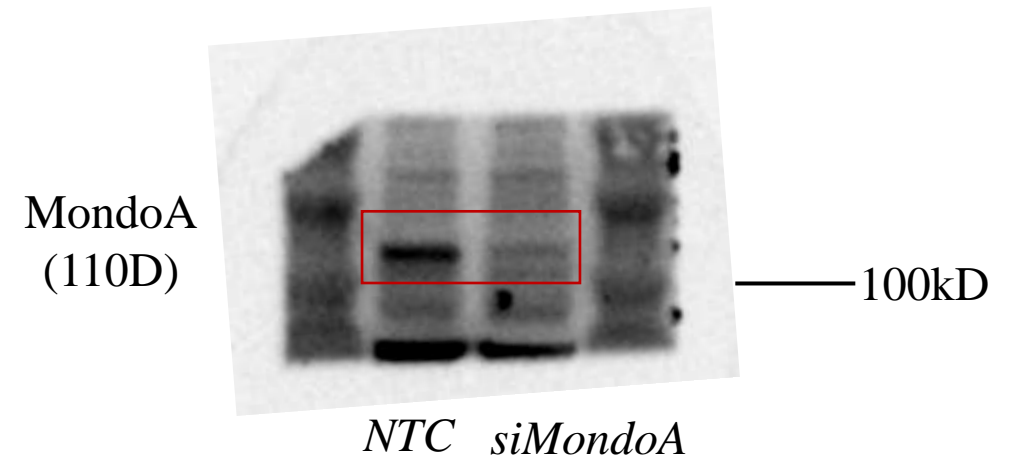

Supplement: Supplementary file 11 — Source data Fig. 3 [file 44321_2024_105_MOESM11_ESM.zip › Figure 3/Figure 3J/Figure 3H.pdf]

**Figure 3H**  
**CRC001**

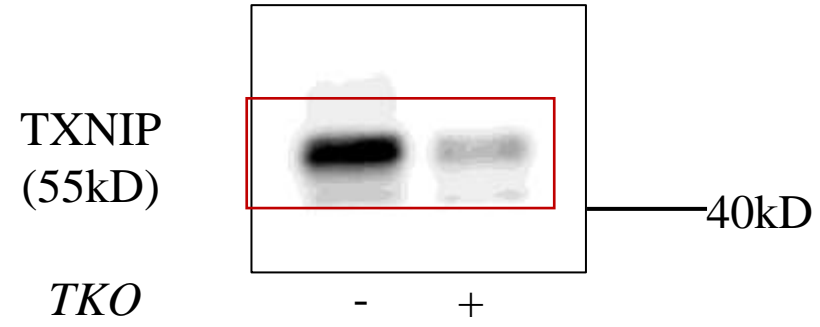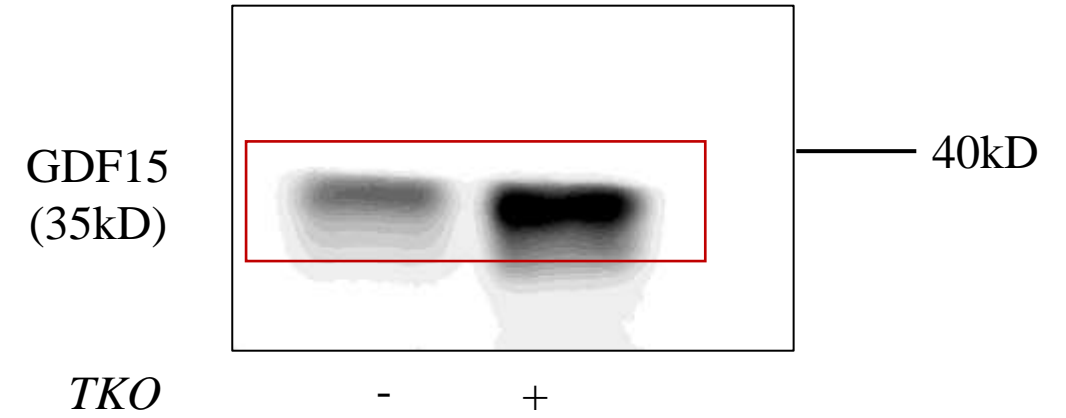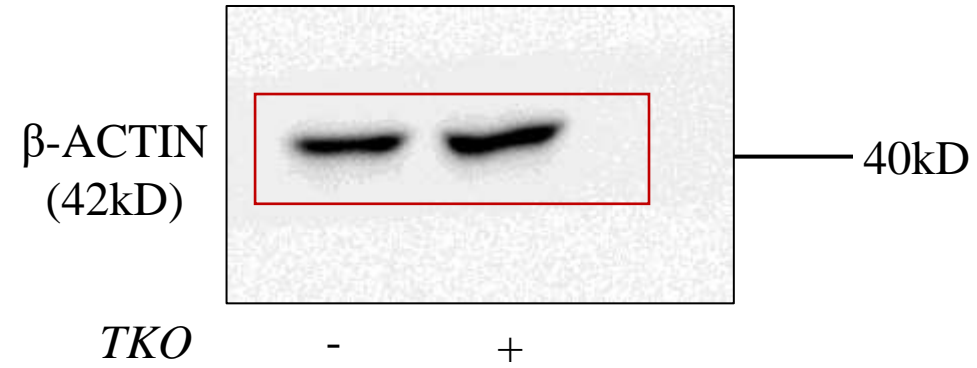

Figure 3H  
CRC002

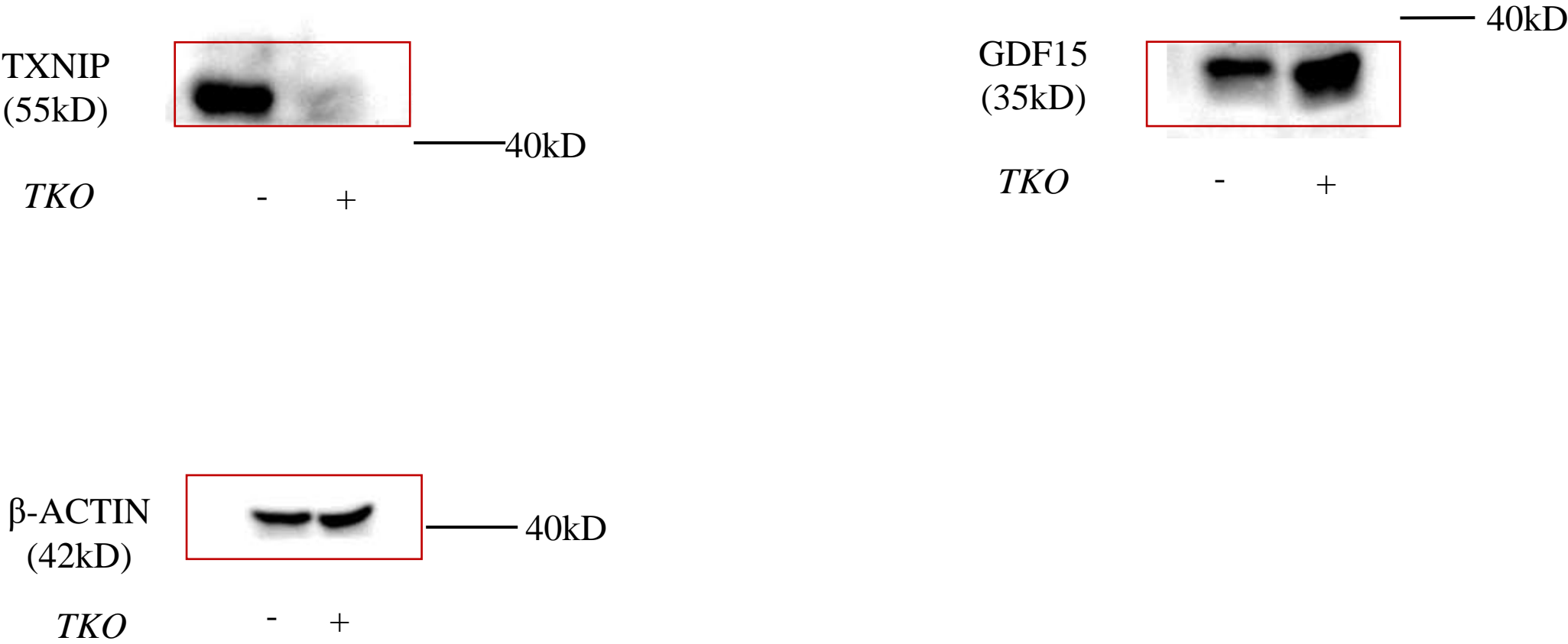

Supplement: Supplementary file 11 — Source data Fig. 3 [file 44321_2024_105_MOESM11_ESM.zip › Figure 3/Figure 3H/Figure 3H.pdf]

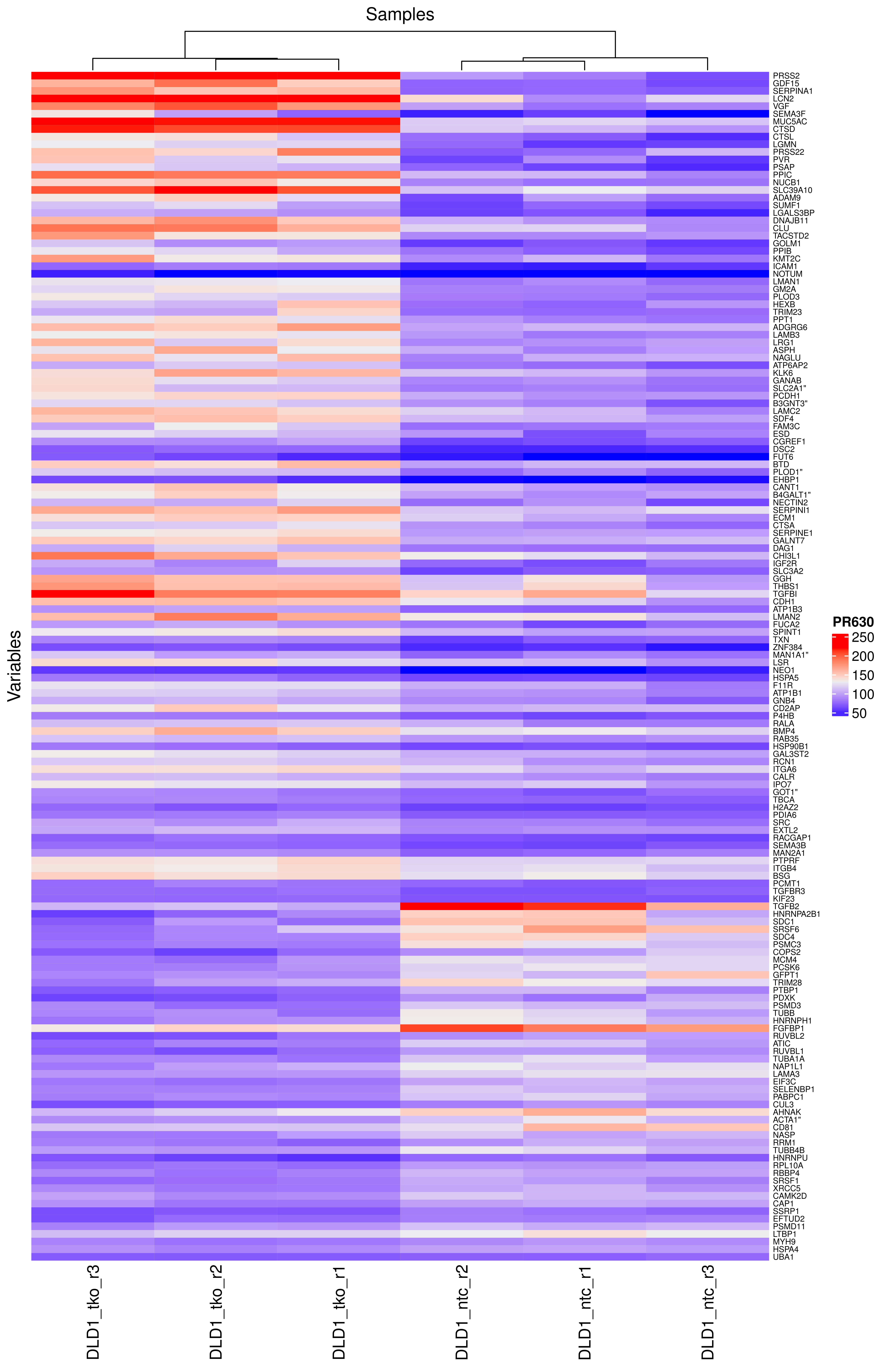

Supplement: Supplementary file 11 — Source data Fig. 3 [file 44321_2024_105_MOESM11_ESM.zip › Figure 3/Figure 3A/Figure 3A.tif]

DAPI/ Epcam/GDF15

CON

OXA

NTC

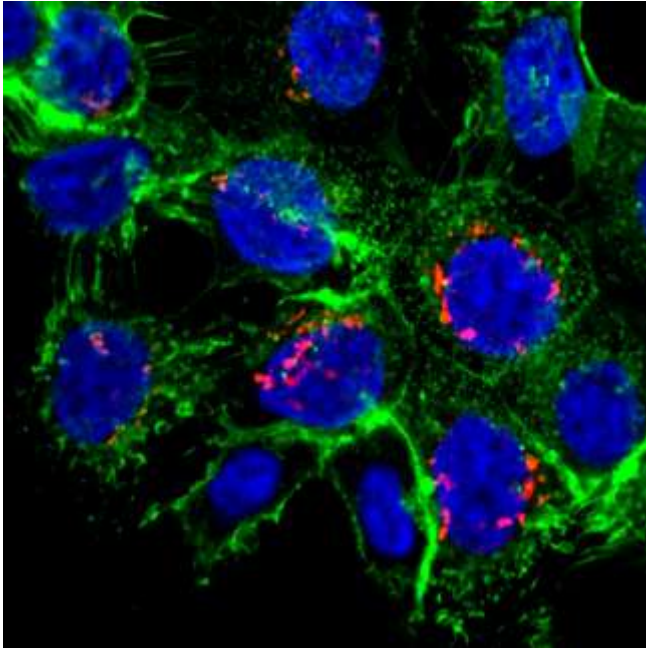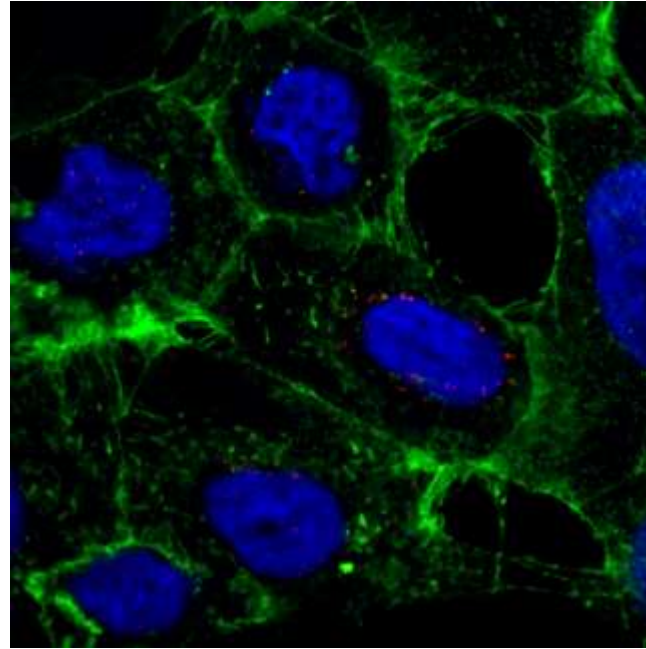

TKO

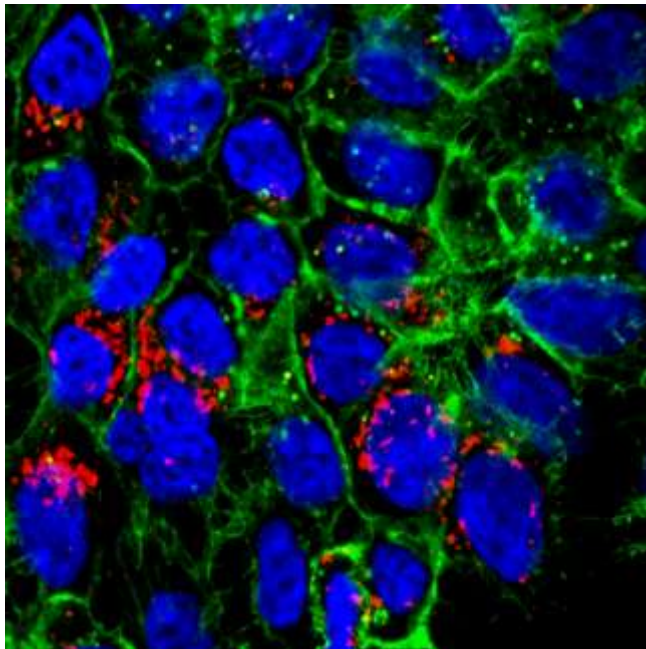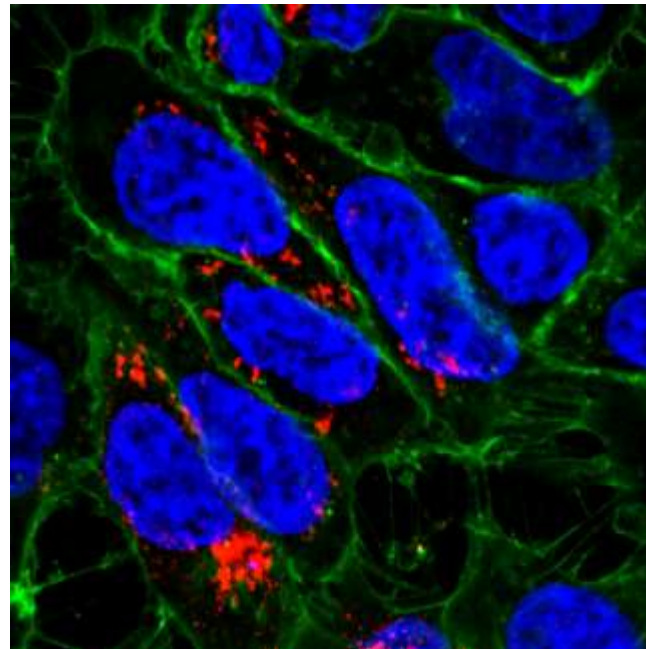

Supplement: Supplementary file 11 — Source data Fig. 3 [file 44321_2024_105_MOESM11_ESM.zip › Figure 3/Figure 3I/Figure 3I.pdf]

Treat-naïve GDF15

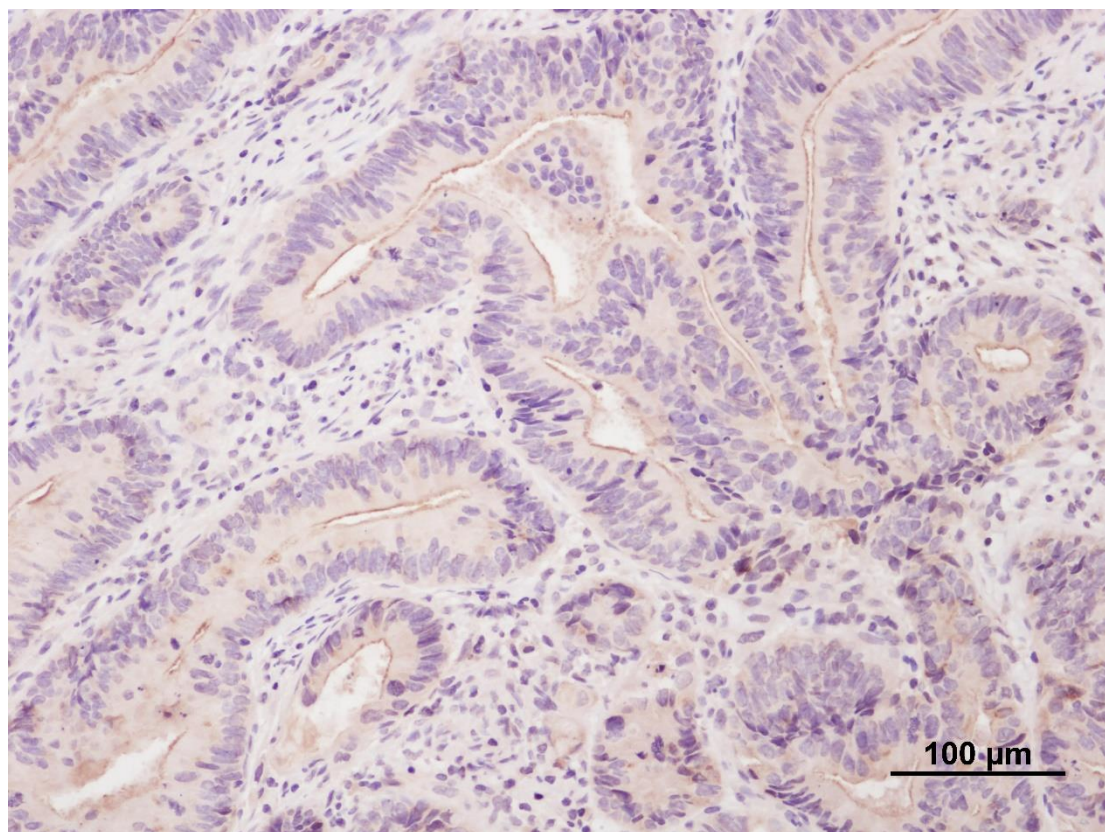

Treat-naïve TXNIP

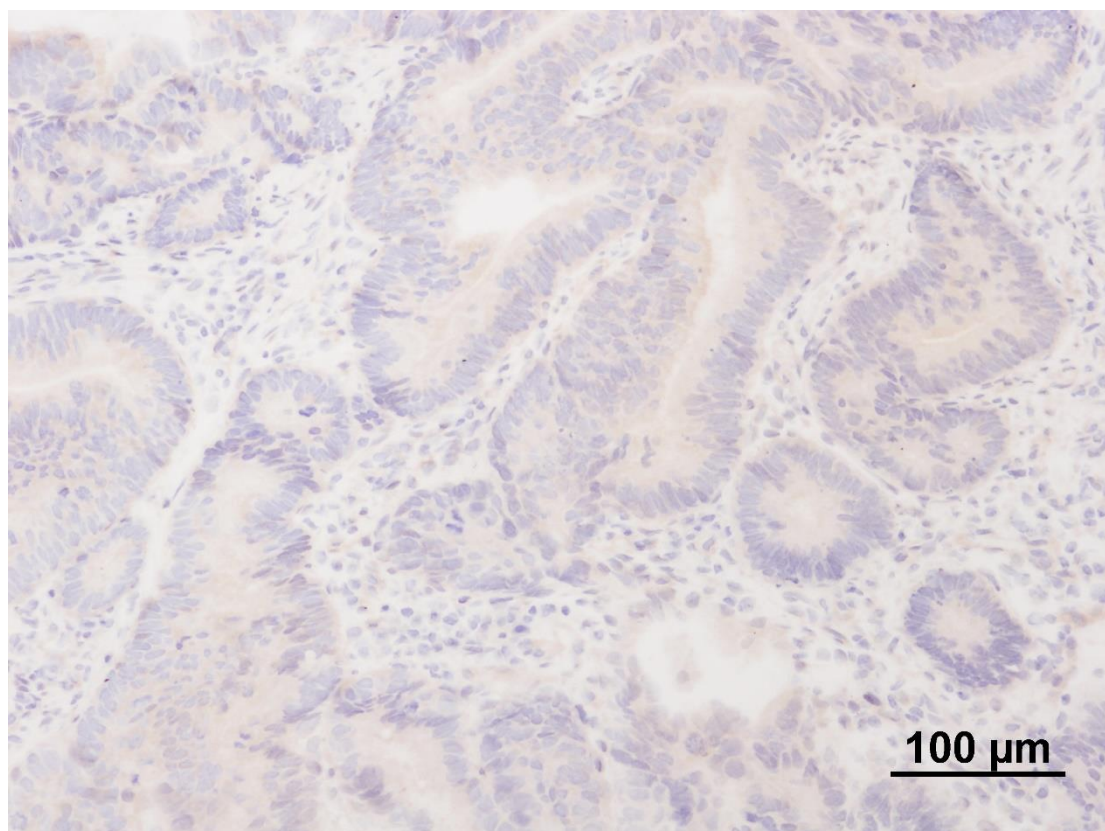

Chemotherapy GDF15

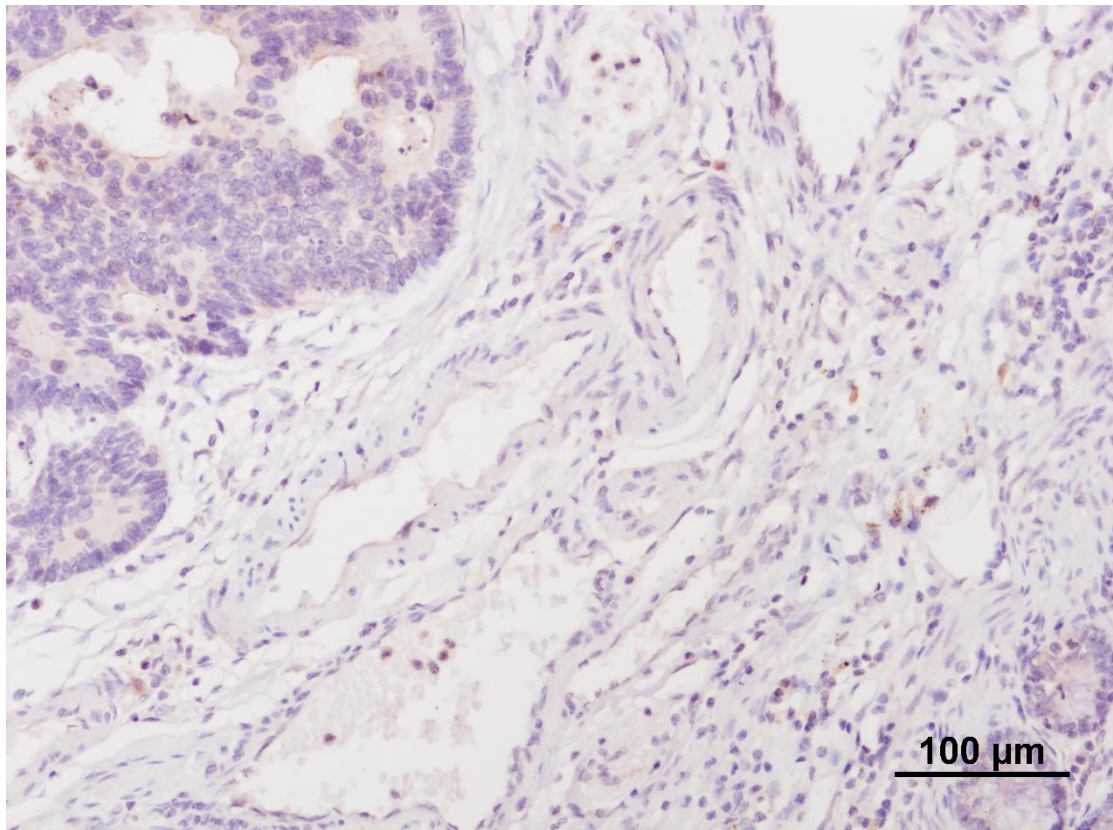

Chemotherapy TXNIP

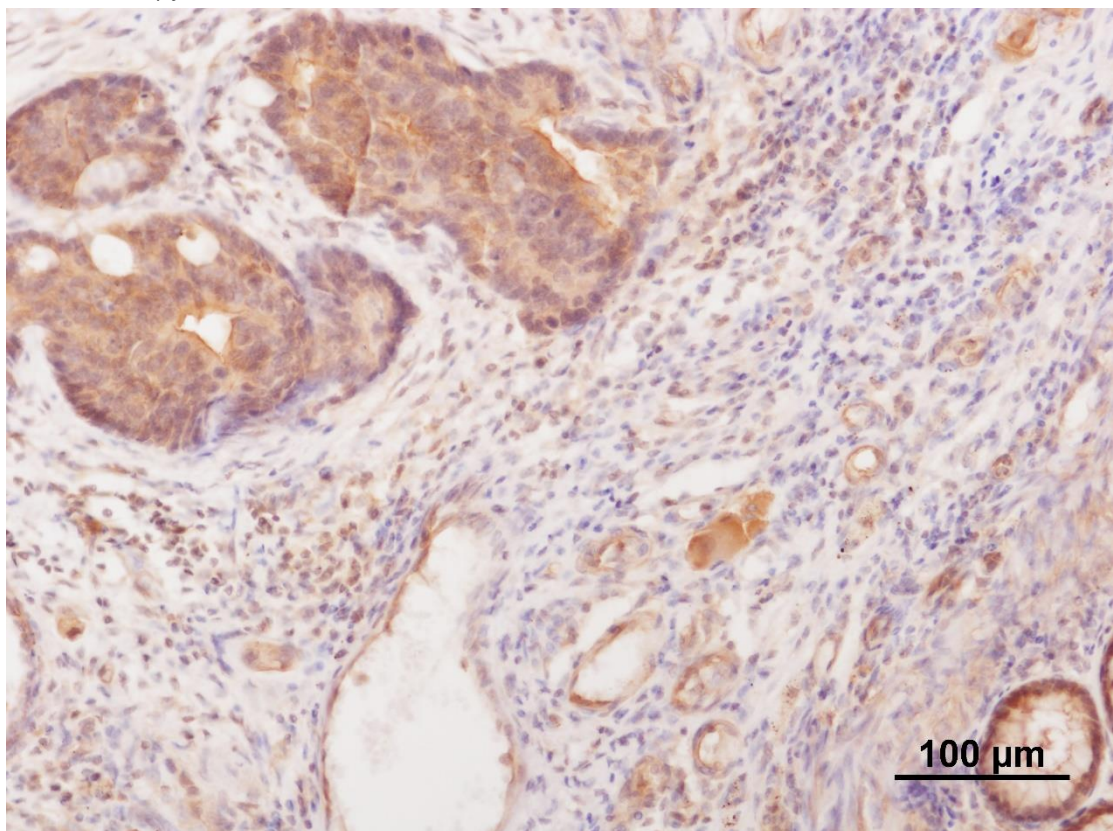

Supplement: Supplementary file 12 — Source data Fig. 4 [file 44321_2024_105_MOESM12_ESM.zip › Figure 4/Figure 4E/Figure 4E.pdf]

H&E ANT

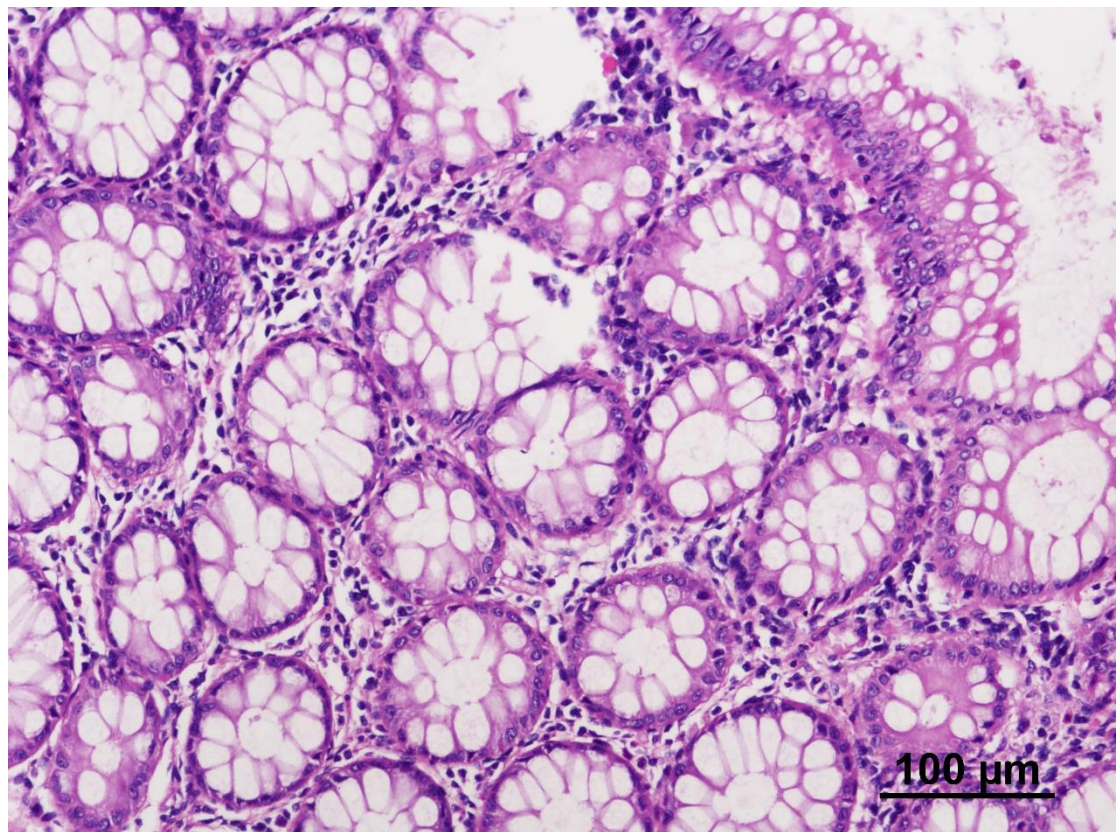

H&E Tumor

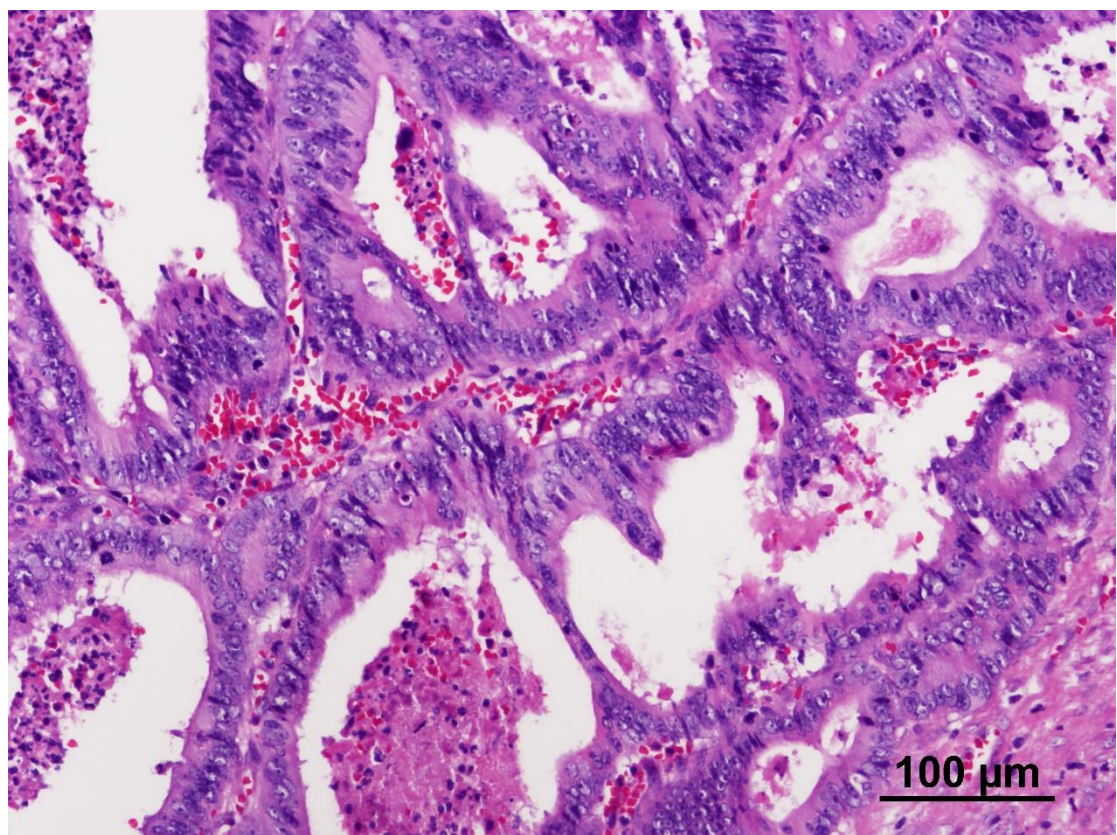

GDF15 ANT

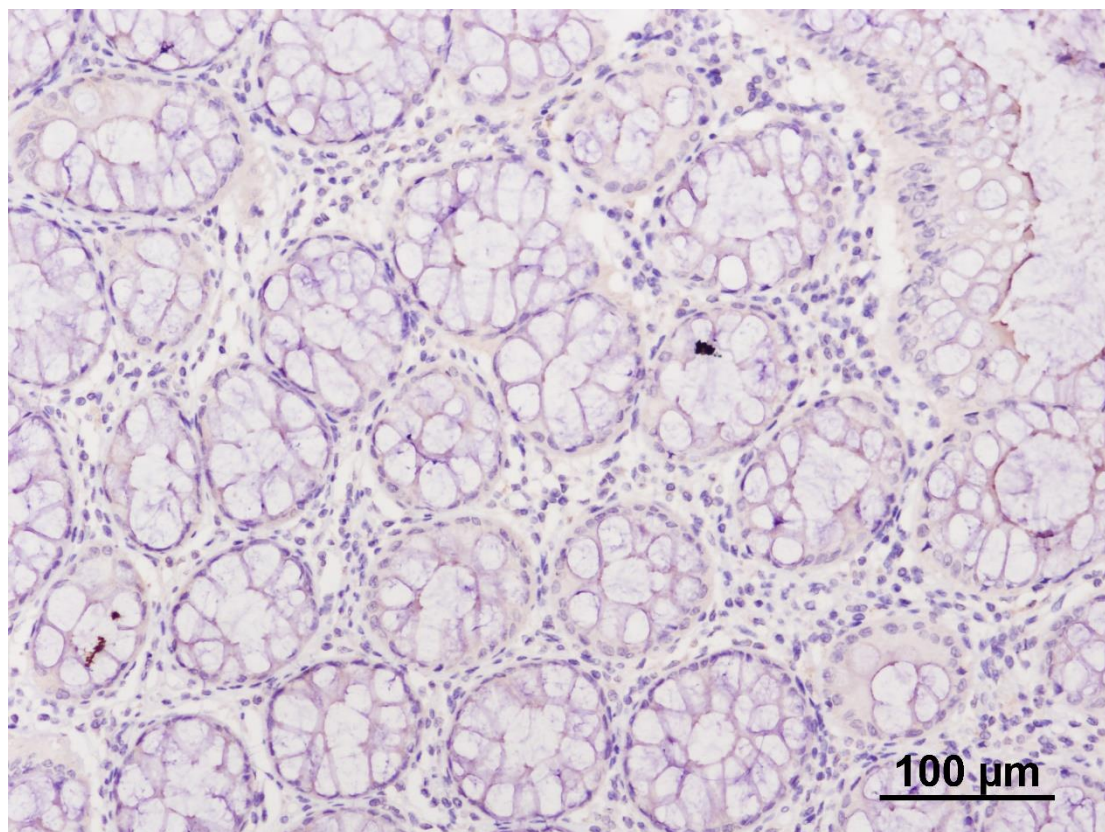

GDF15 Tumor

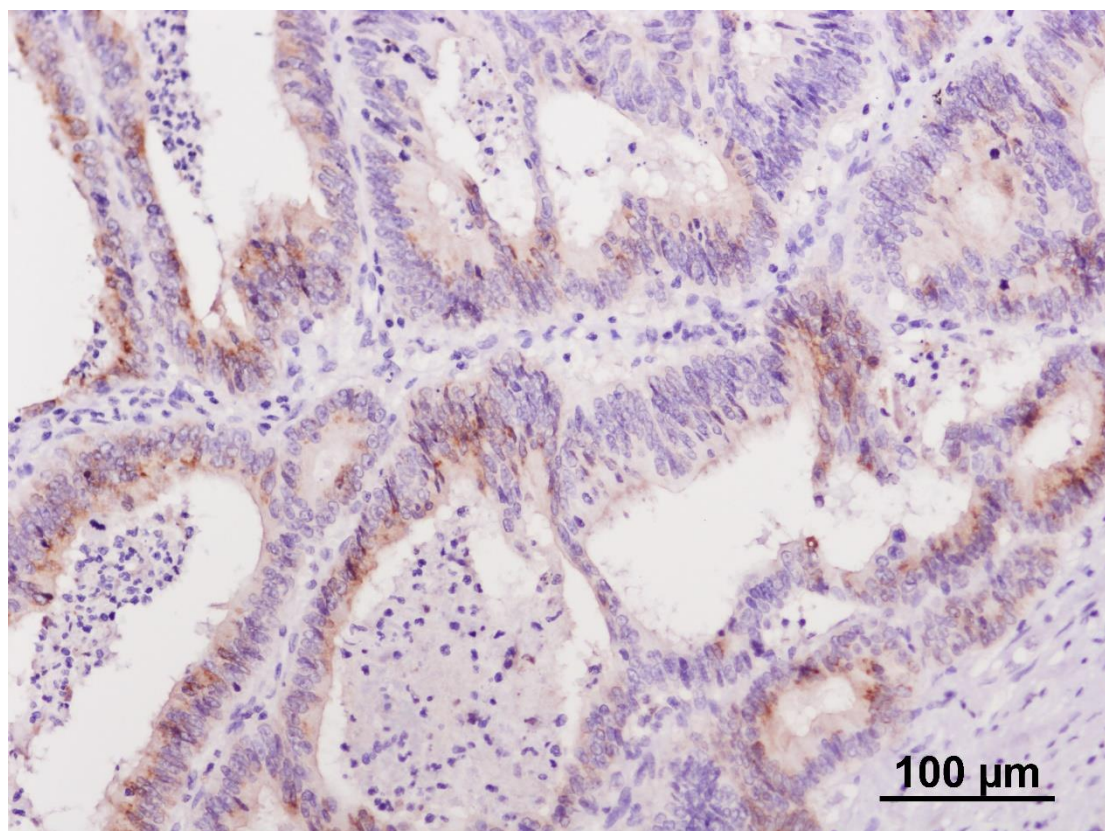

Supplement: Supplementary file 12 — Source data Fig. 4 [file 44321_2024_105_MOESM12_ESM.zip › Figure 4/Figure 4A/Figure 4A.pdf]

Figure 4F

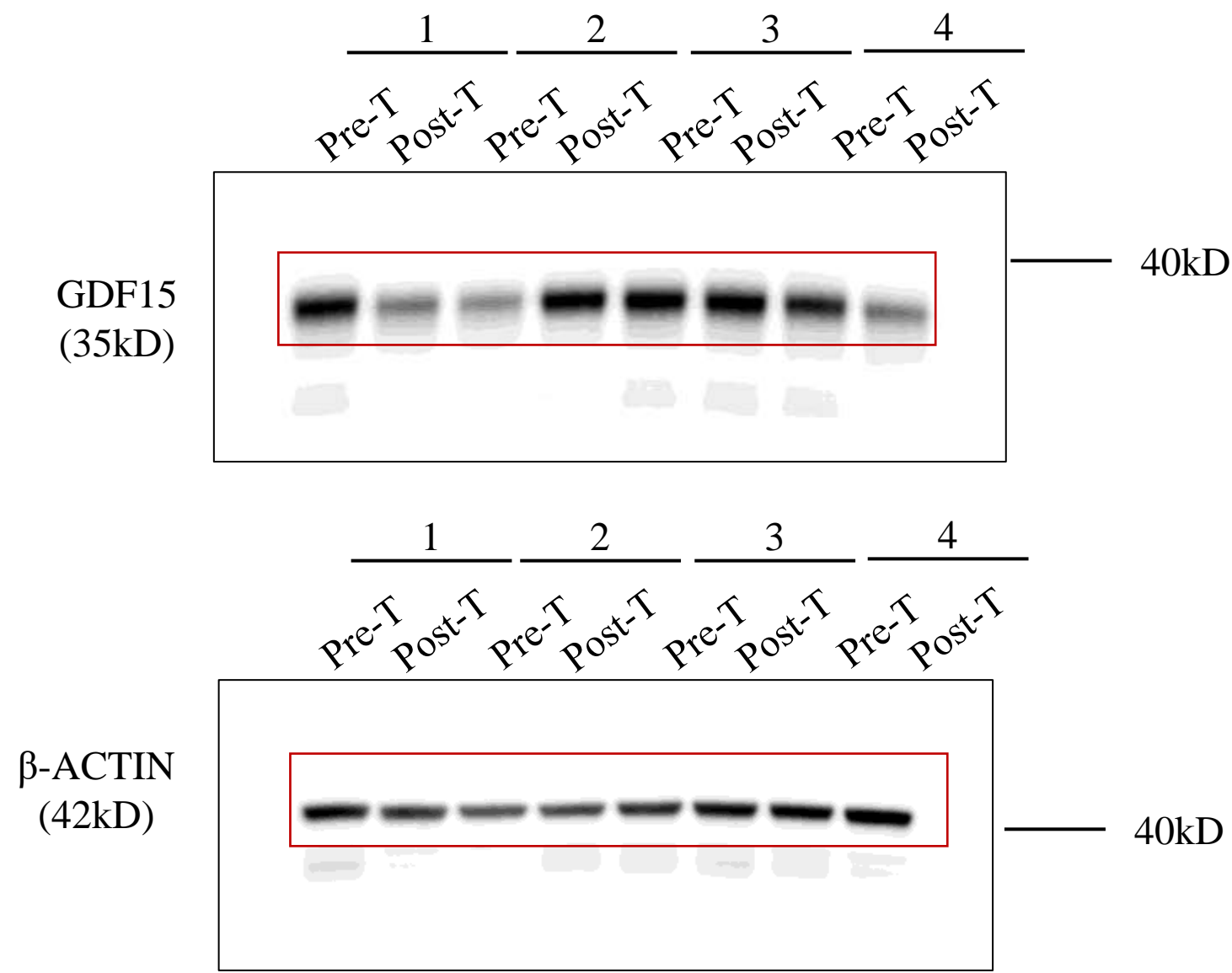

### Figure 4F

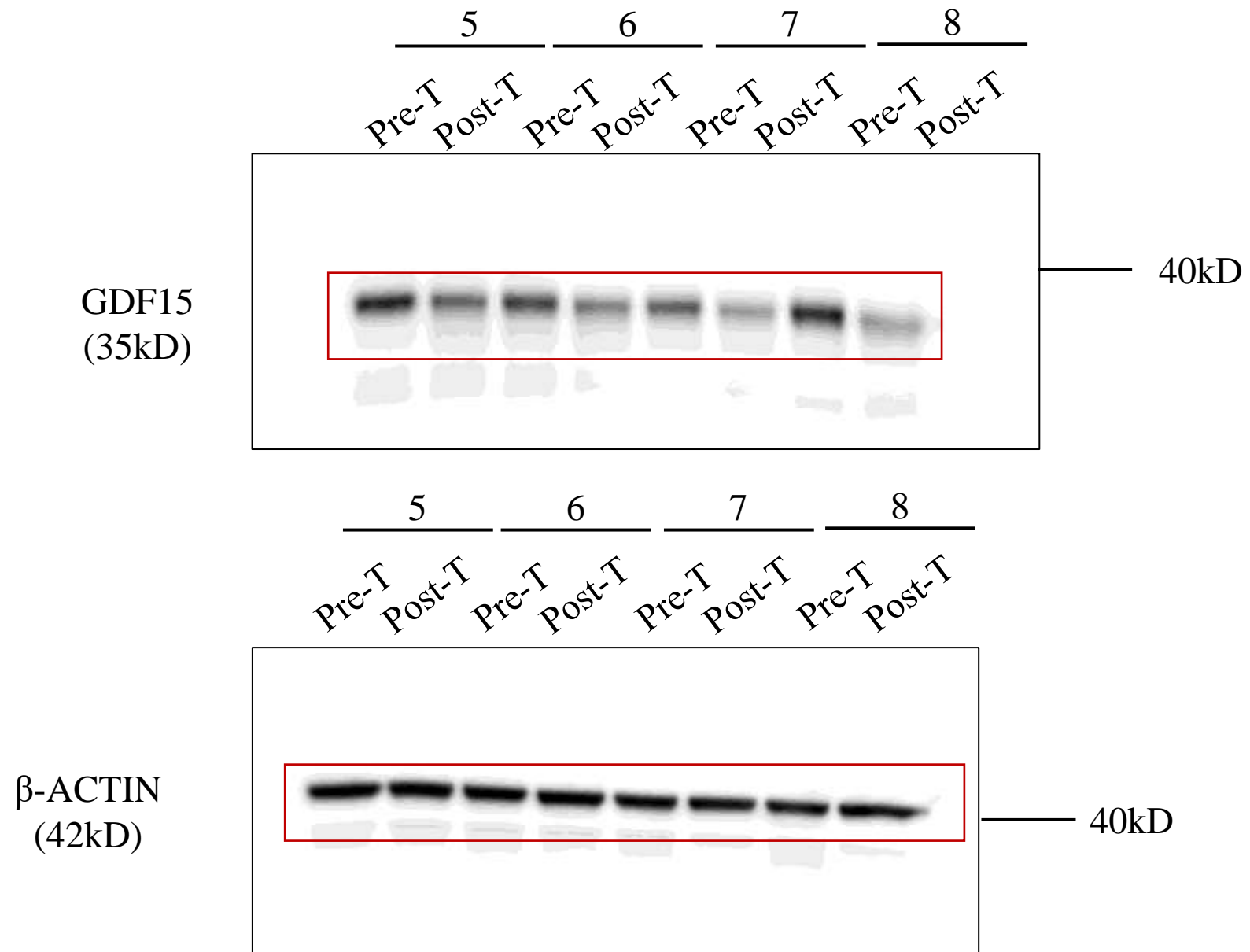

**Figure 4F**

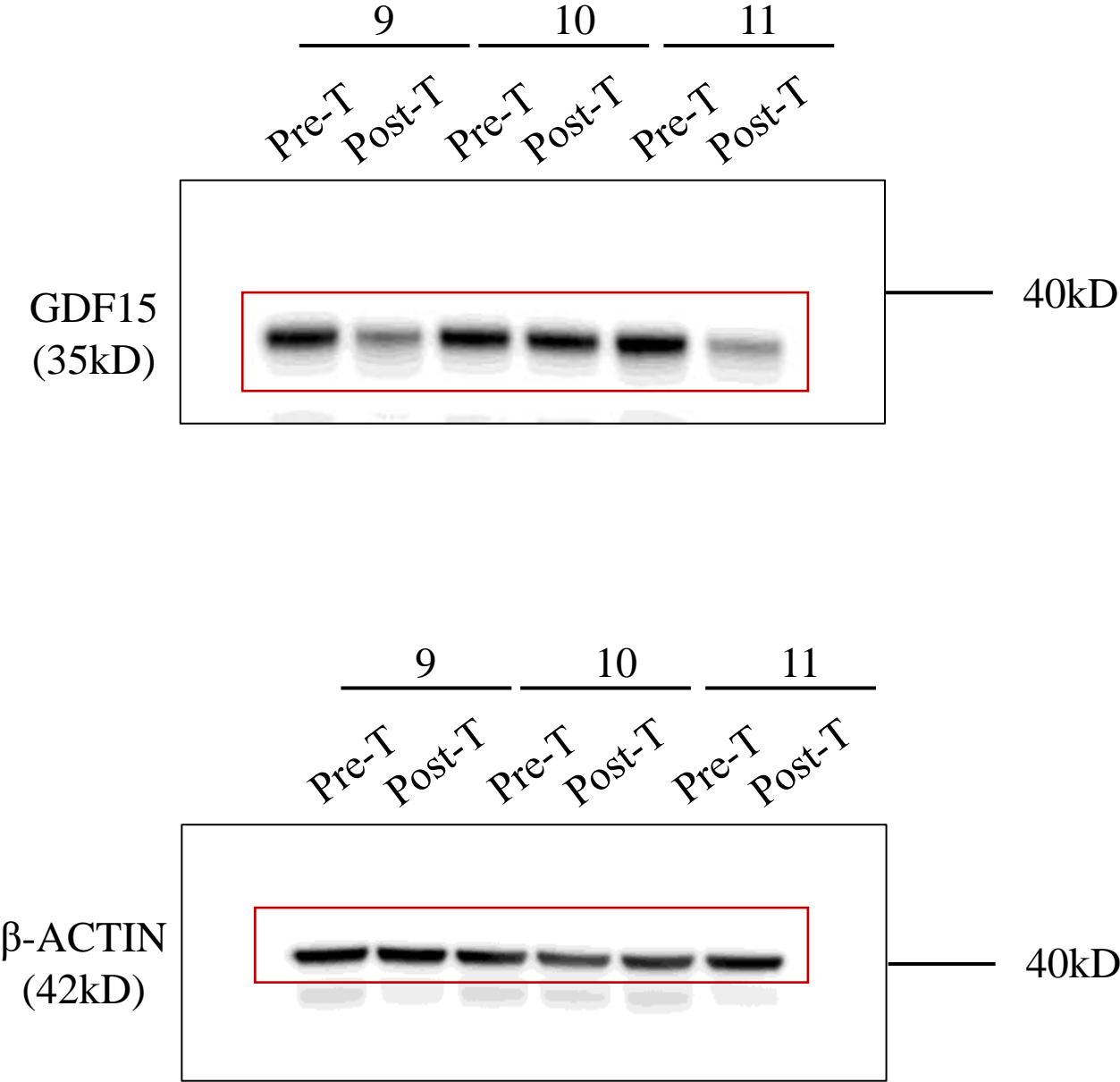

Supplement: Supplementary file 12 — Source data Fig. 4 [file 44321_2024_105_MOESM12_ESM.zip › Figure 4/Figure 4F/Figure 4F.pdf]

GDF15-GDF15 low

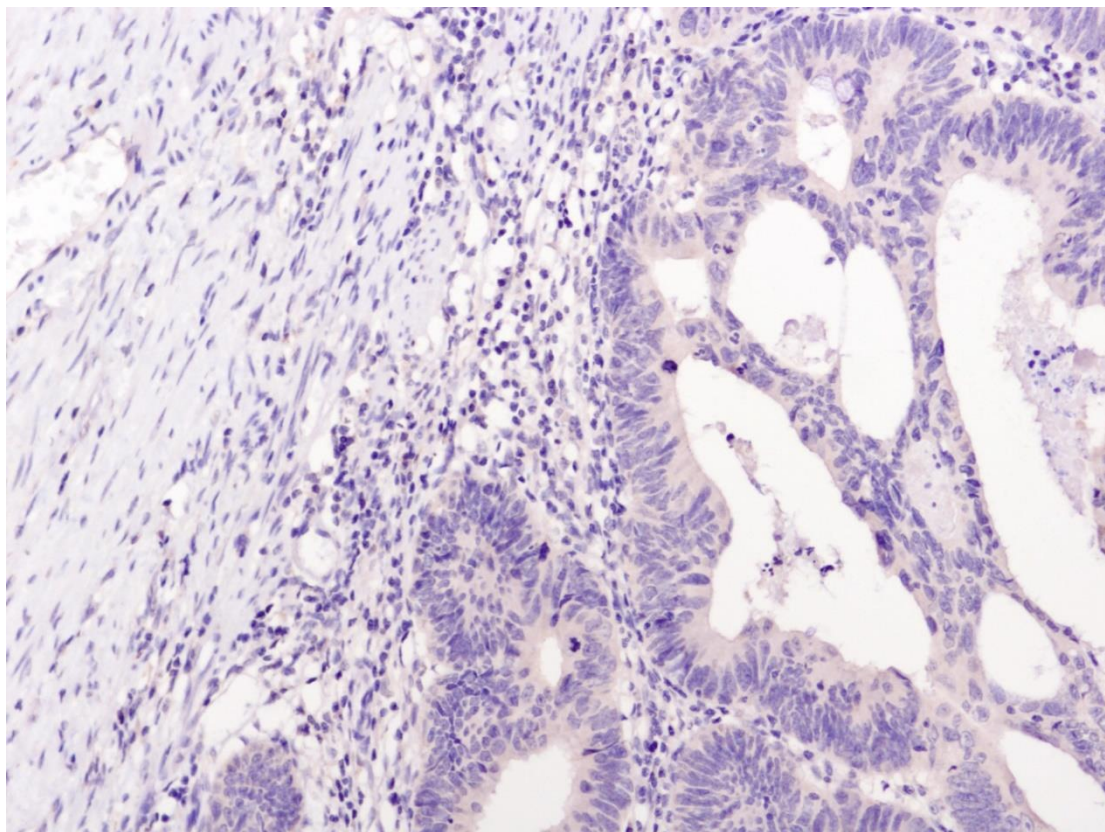

GDF15-GDF15 medium

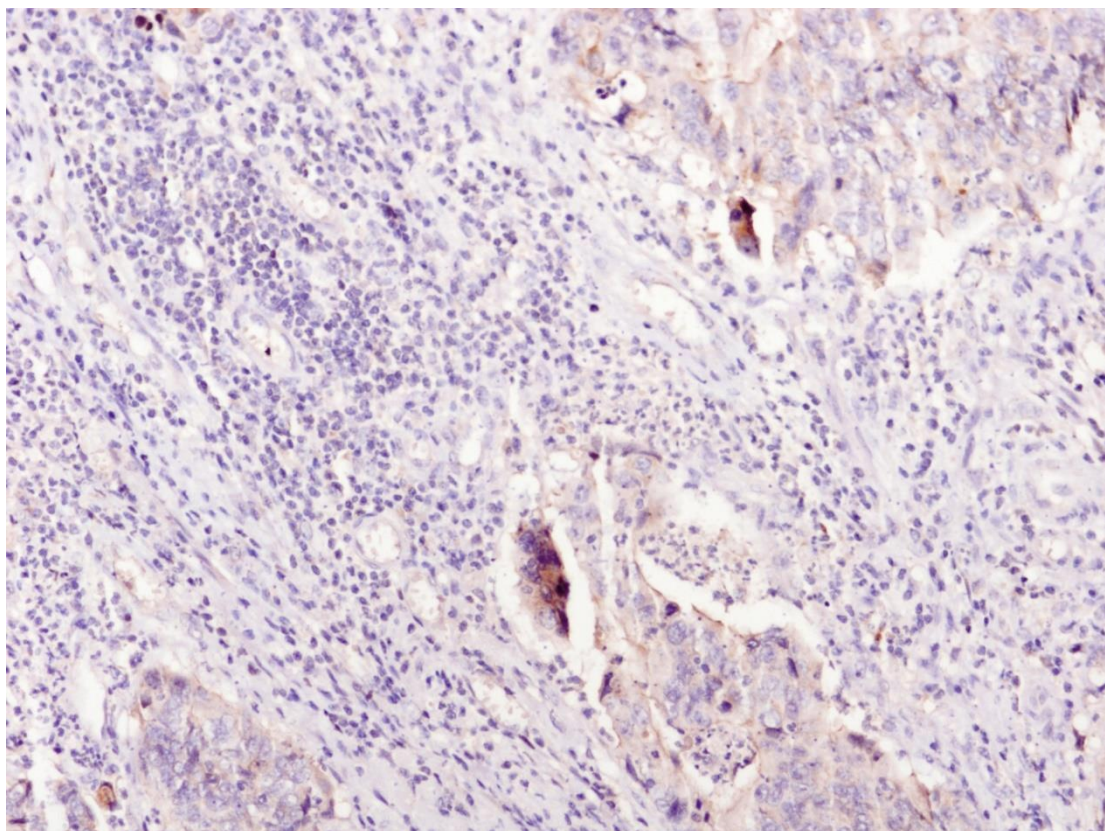

GDF15-GDF15 high

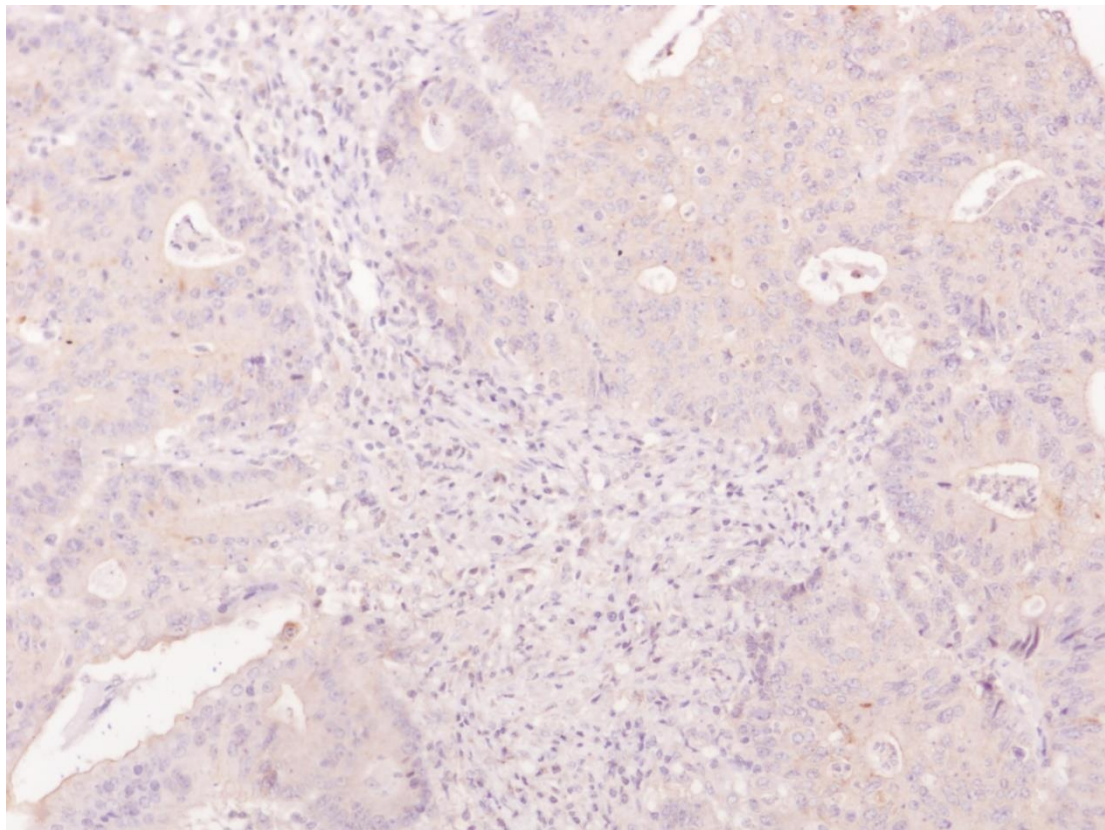

FOXP3-GDF15 low

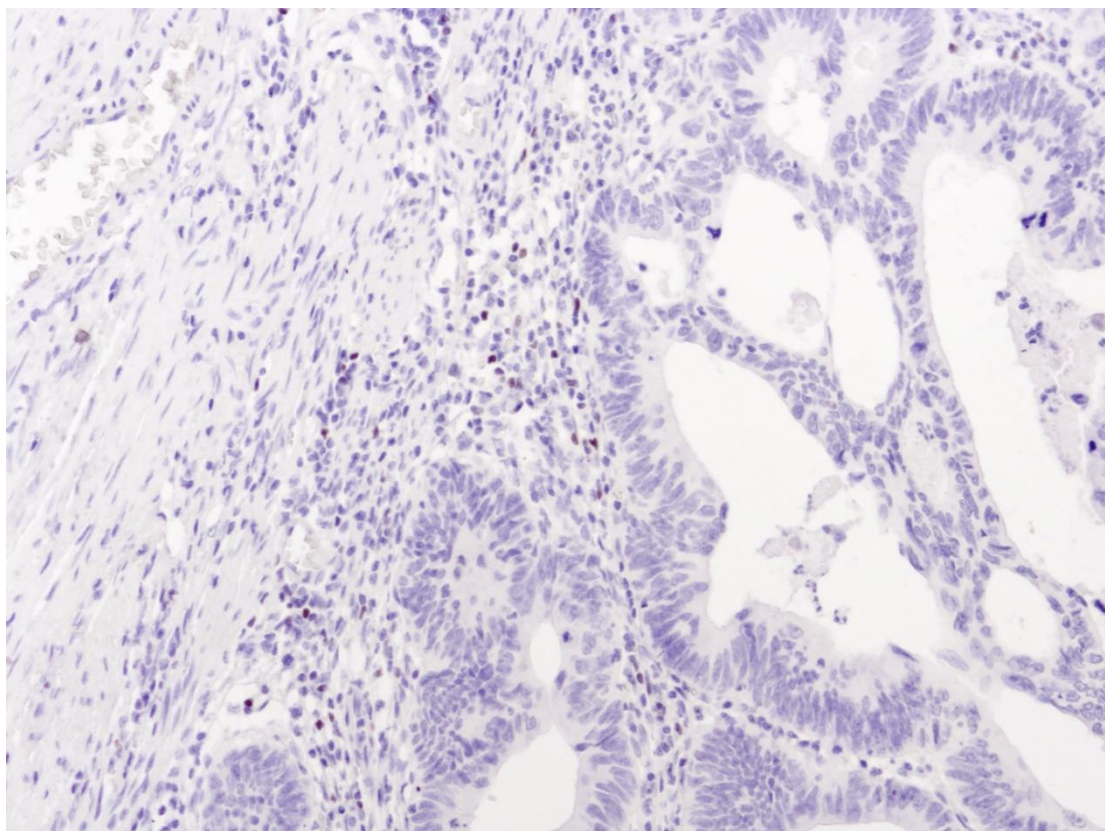

FOXP3-GDF15 medium

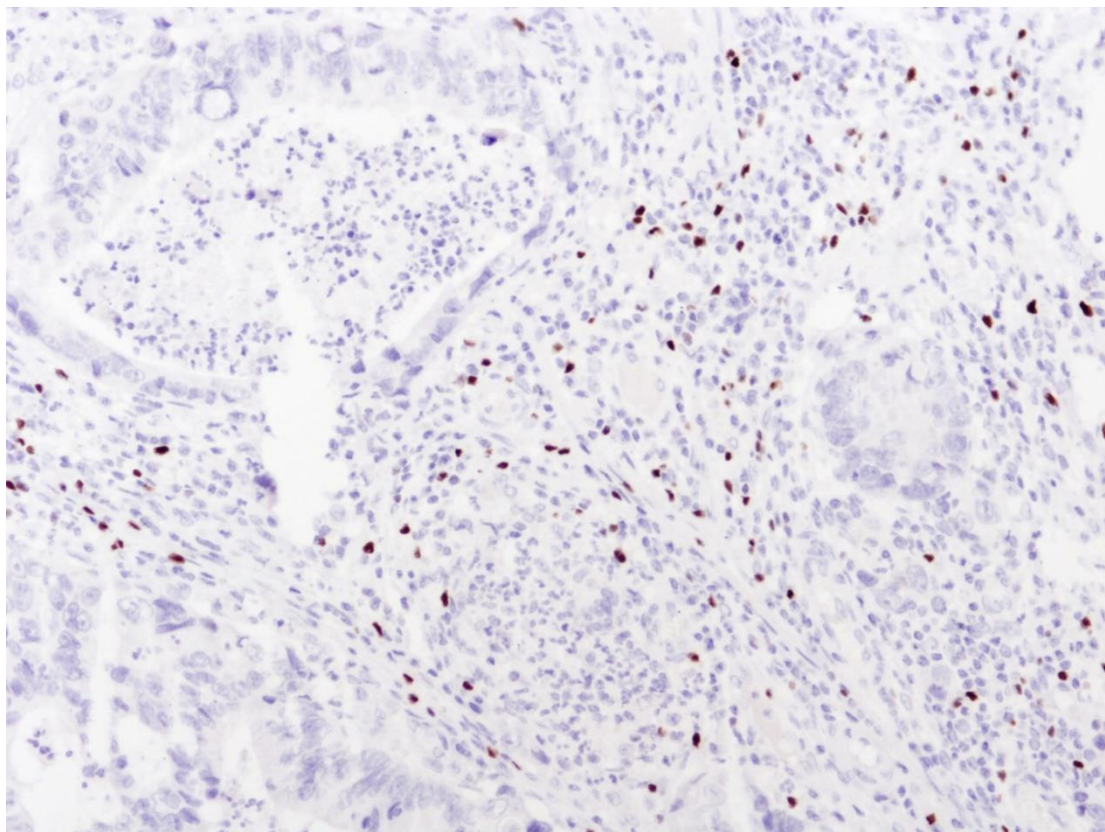

FOXP3-GDF15 high

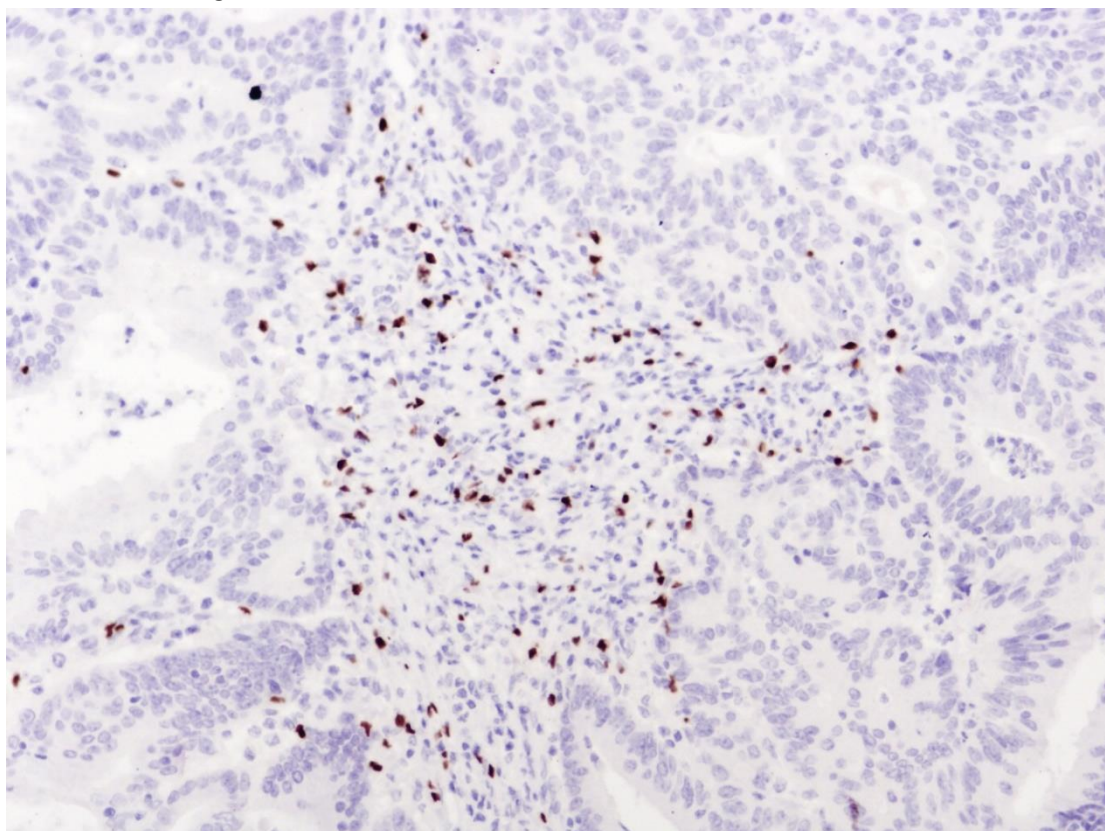

Supplement: Supplementary file 13 — Source data Fig. 5 [file 44321_2024_105_MOESM13_ESM.zip › Figure 5/Figure 5K/Figure 5K.pdf]

**Figure 6F**

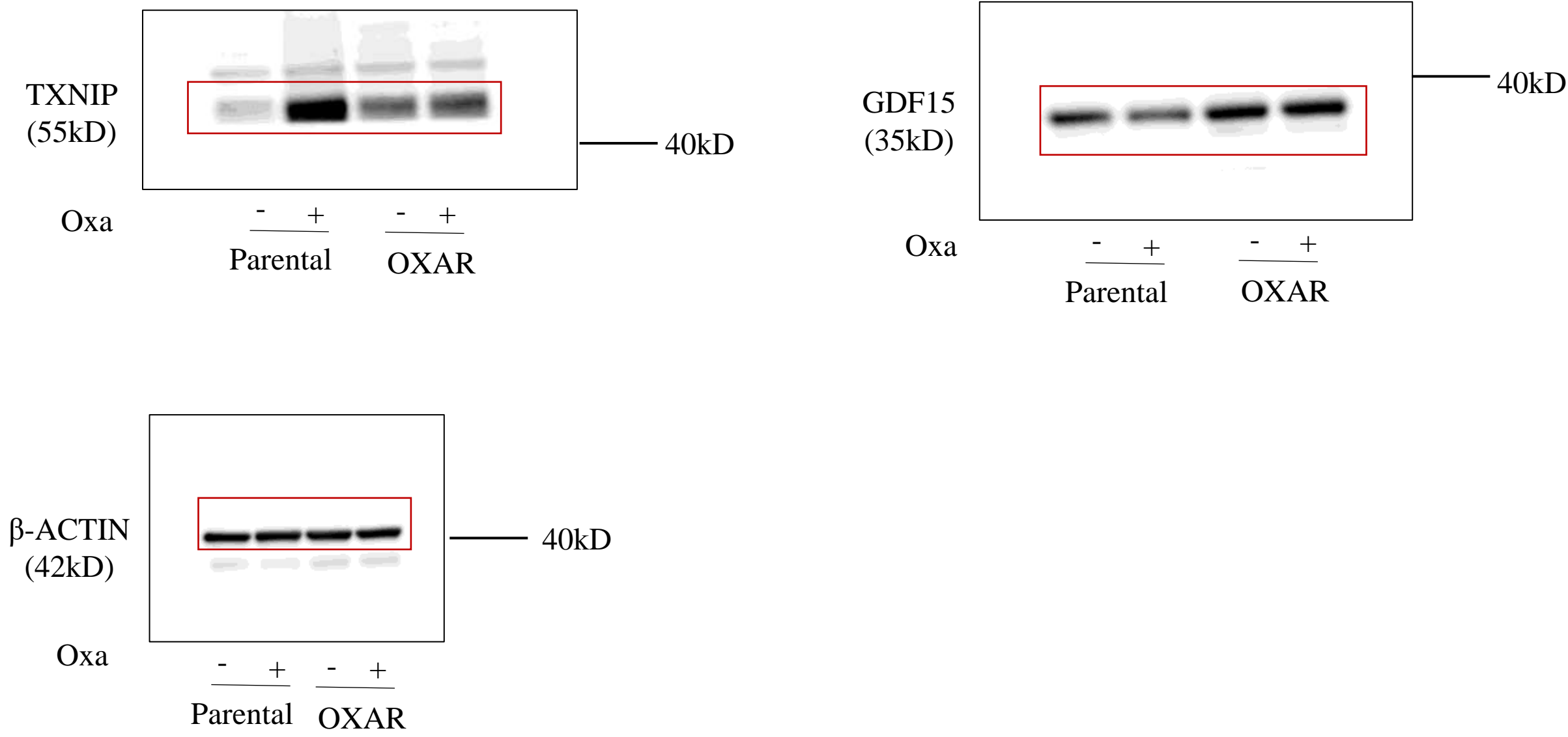

Supplement: Supplementary file 14 — Source data Fig. 6 [file 44321_2024_105_MOESM14_ESM.zip › Figure 6/Figure 6F/Figure 6F.pdf]

**Figure 6A-DLD1**

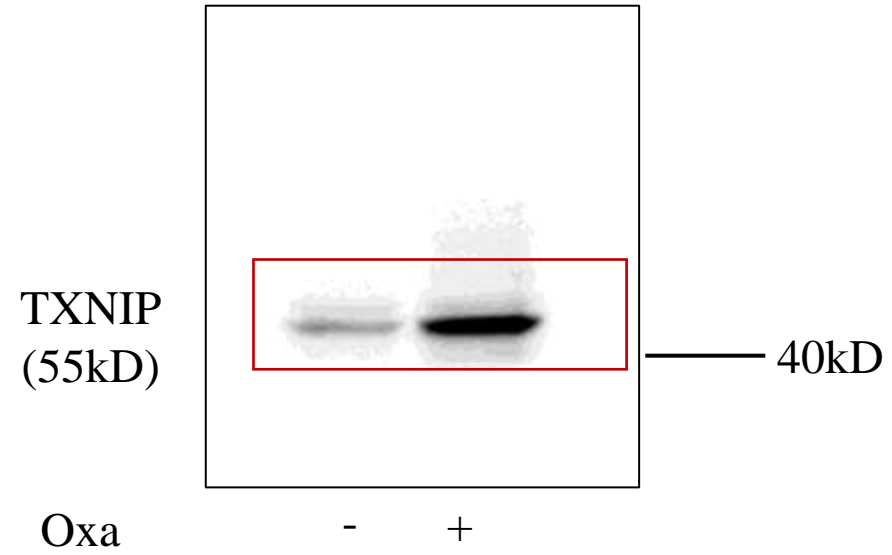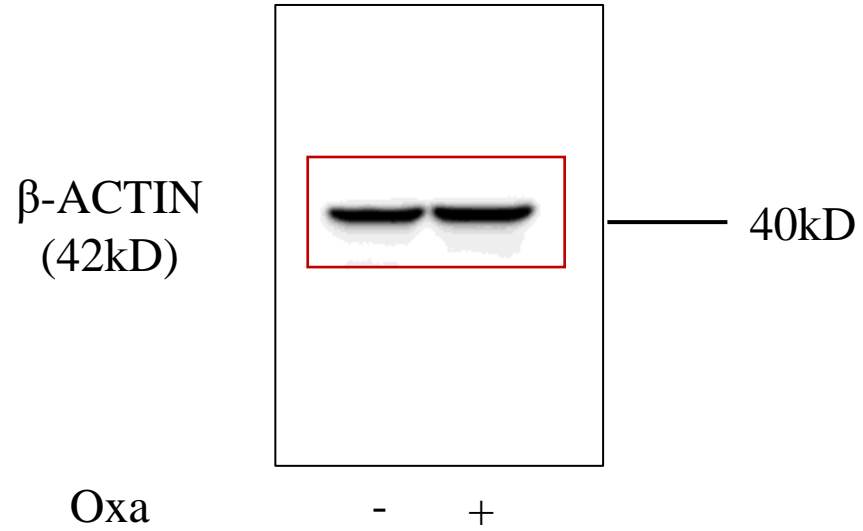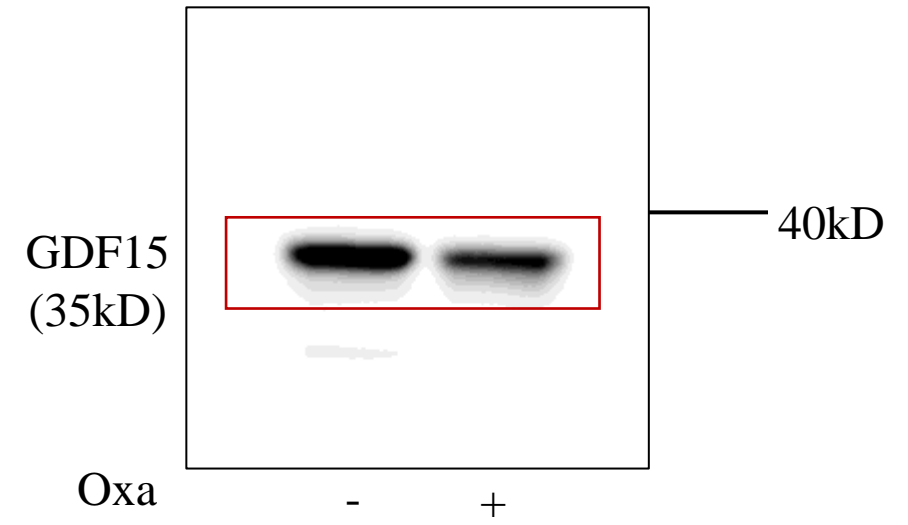

**Figure 6A-HCT15**

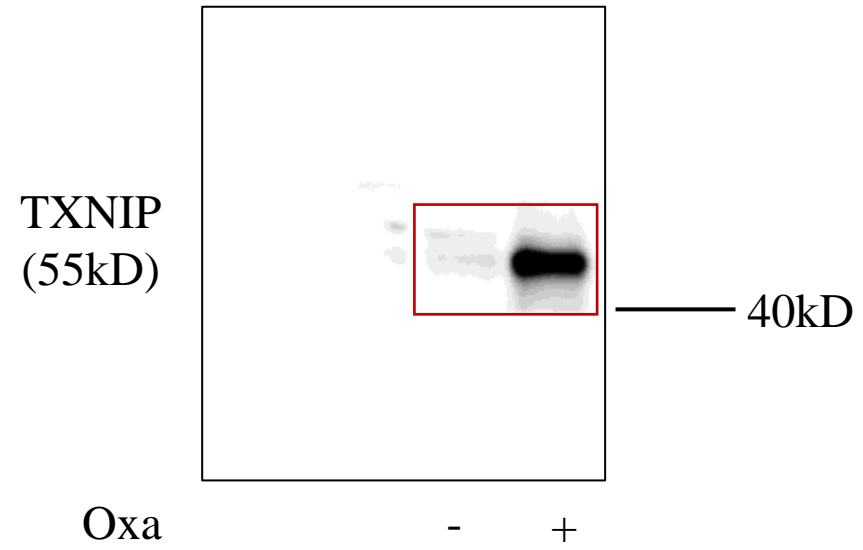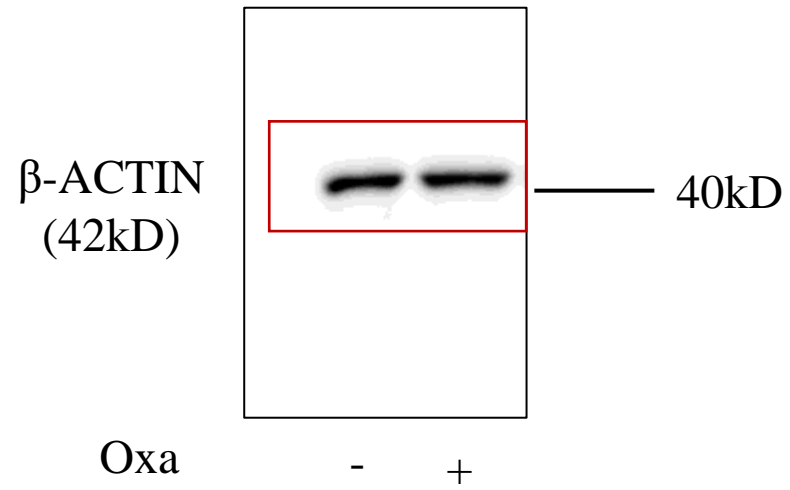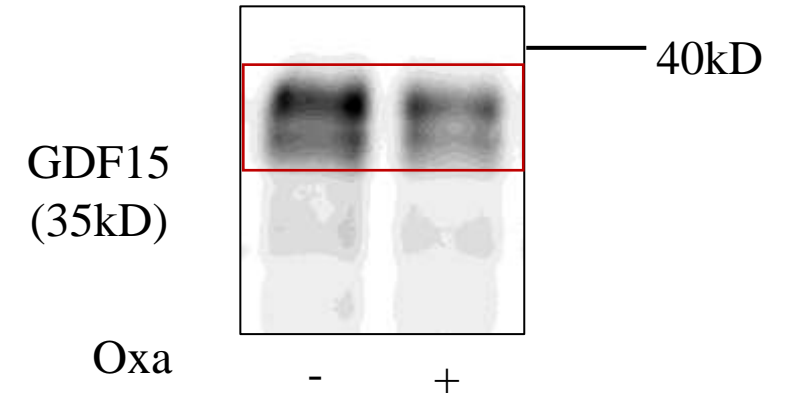

**Figure 6A-HT29**

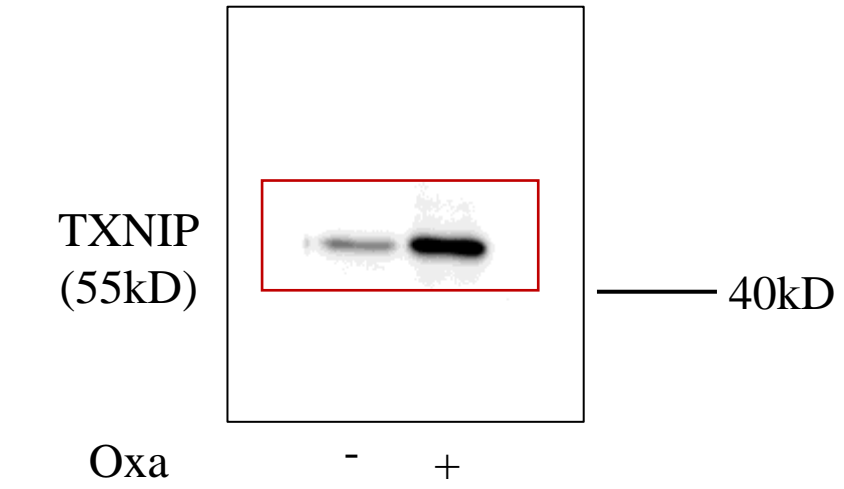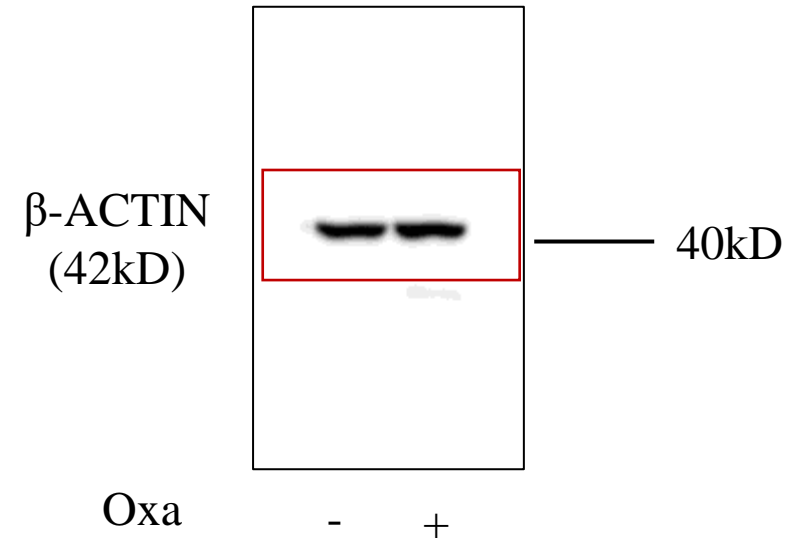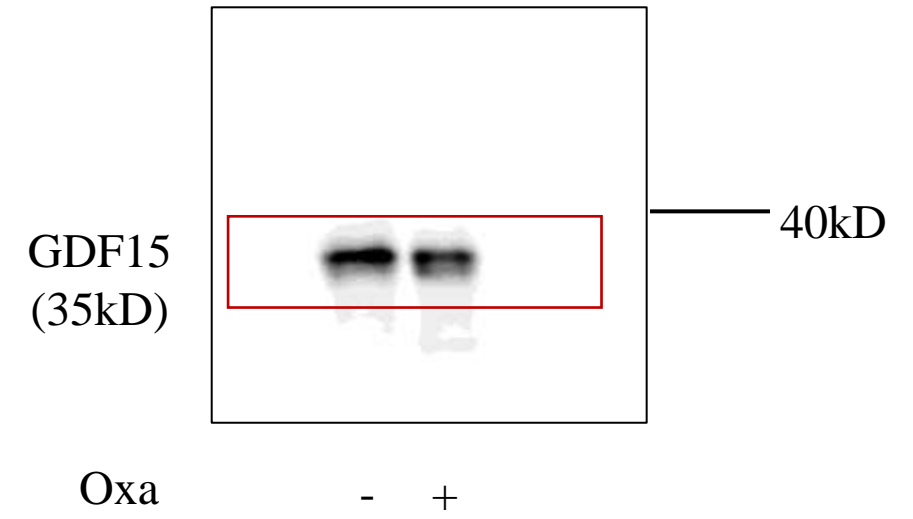

**Figure 6A-SW48**

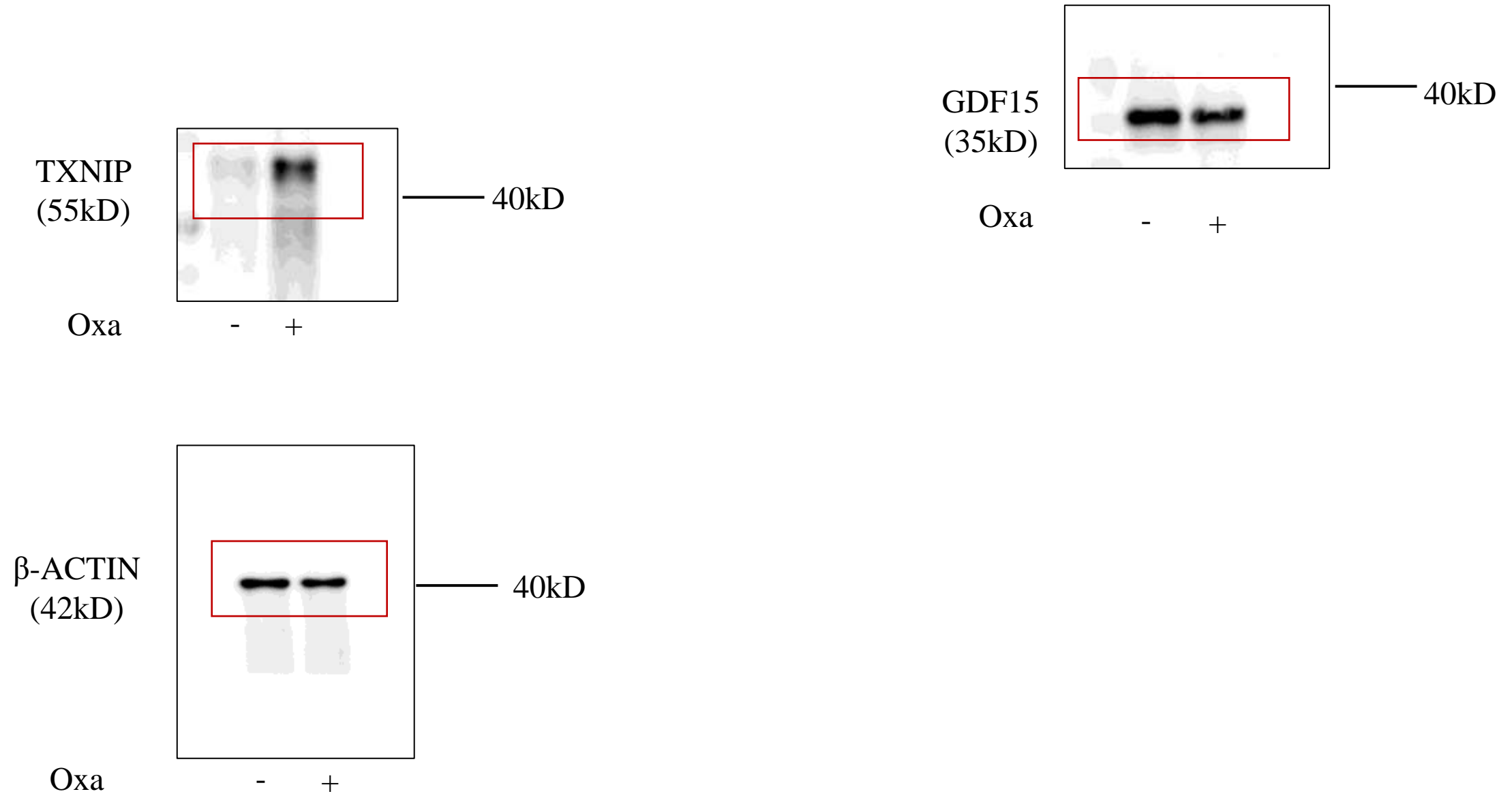

Supplement: Supplementary file 14 — Source data Fig. 6 [file 44321_2024_105_MOESM14_ESM.zip › Figure 6/Figure 6A/Figure 6A.pdf]

Figure 6G

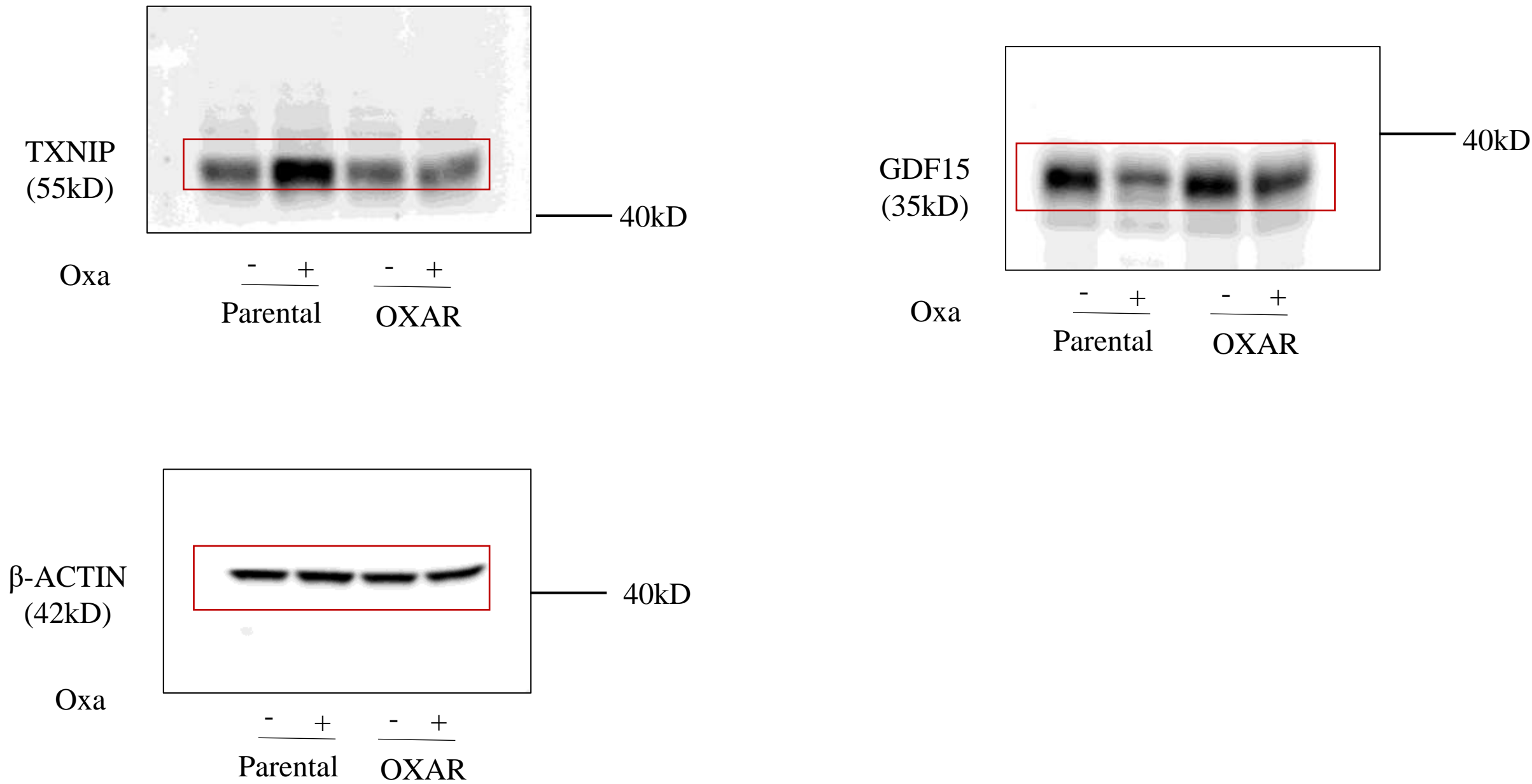

Supplement: Supplementary file 14 — Source data Fig. 6 [file 44321_2024_105_MOESM14_ESM.zip › Figure 6/Figure 6G/Figure 6G.pdf]

Figure 6B-DiFi

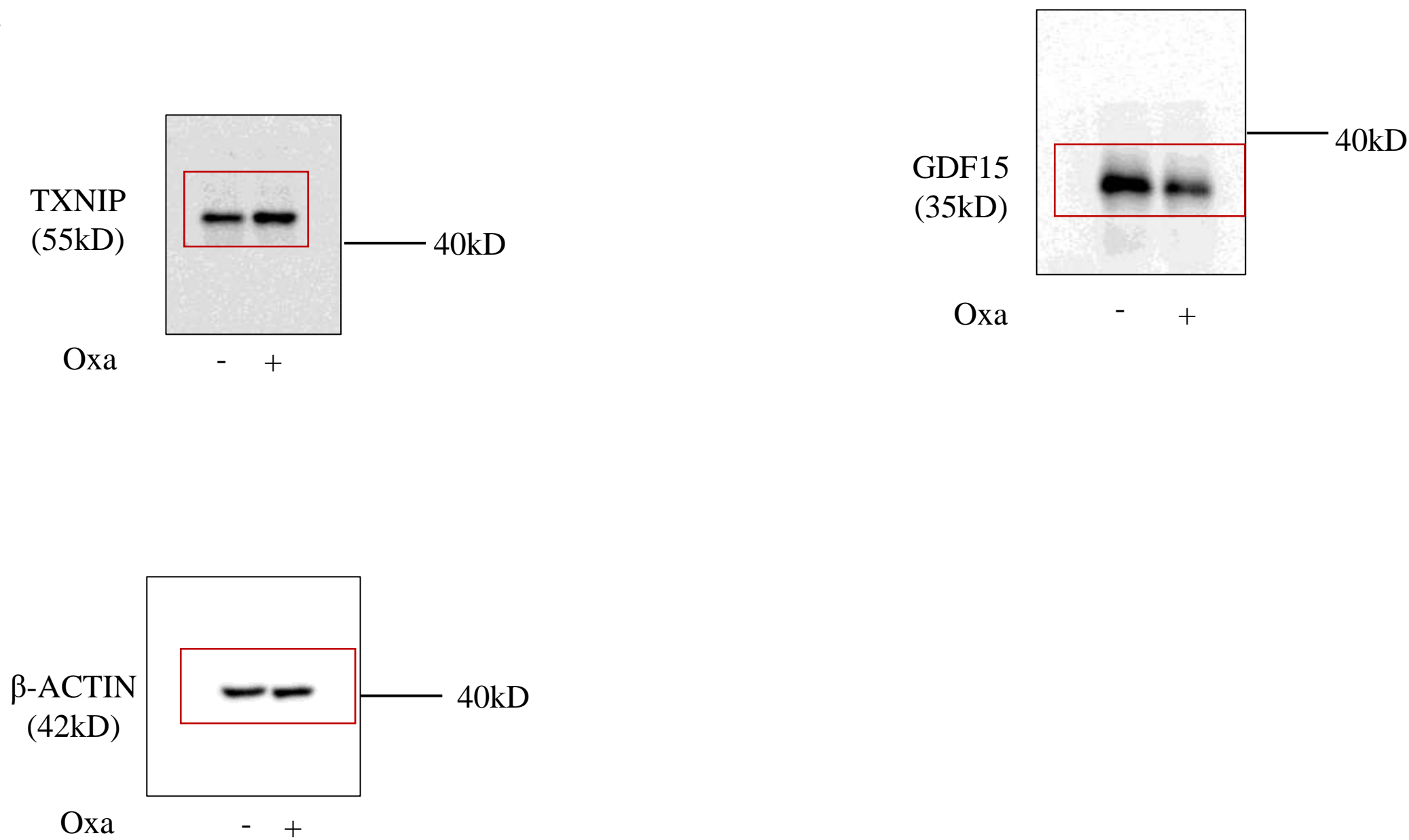

**Figure 6B-LIM1215**

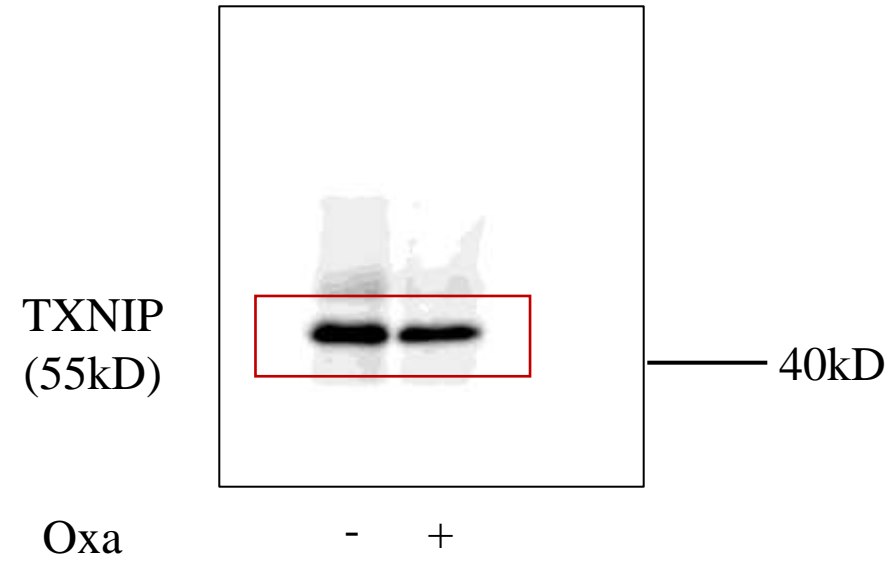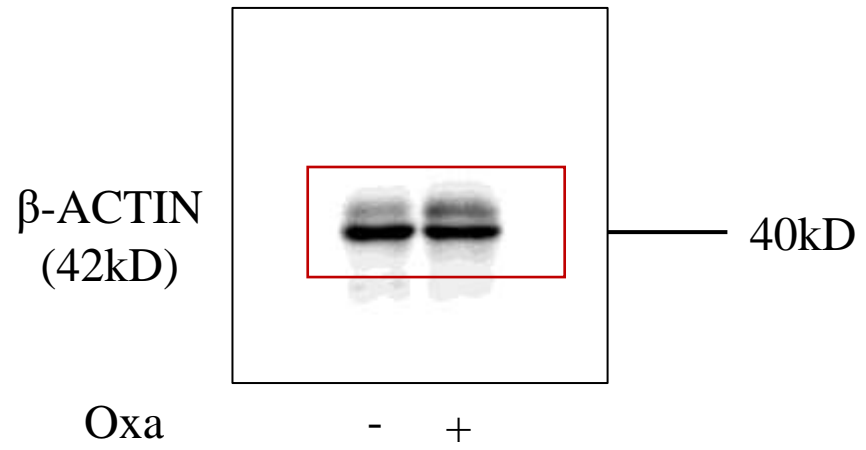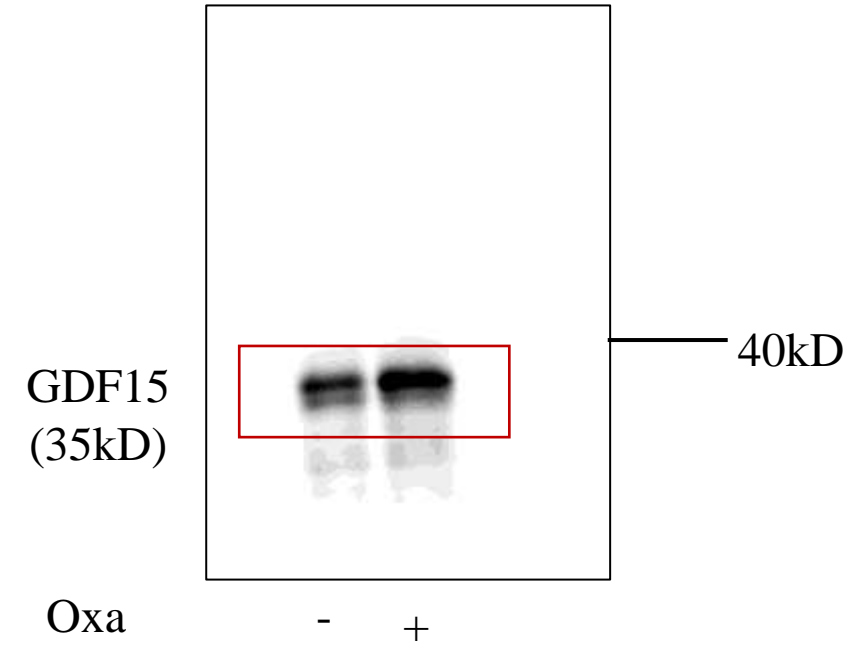

Supplement: Supplementary file 14 — Source data Fig. 6 [file 44321_2024_105_MOESM14_ESM.zip › Figure 6/Figure 6B/Figure 6B.pdf]
