# Supplementary material for: The MondoA-dependent TXNIP/GDF15 axis predicts oxaliplatin response in colorectal adenocarcinomas
Source: EMBO Mol Med. 2024 Aug 5;16(9):7. doi: 10.1038/s44321-024-00105-2 (PMC11393413; doi:10.1038/s44321-024-00105-2)
Supplement: Supplementary file 16 — Expanded View Figures [file 44321_2024_105_MOESM16_ESM.pdf]

# Expanded View Figures

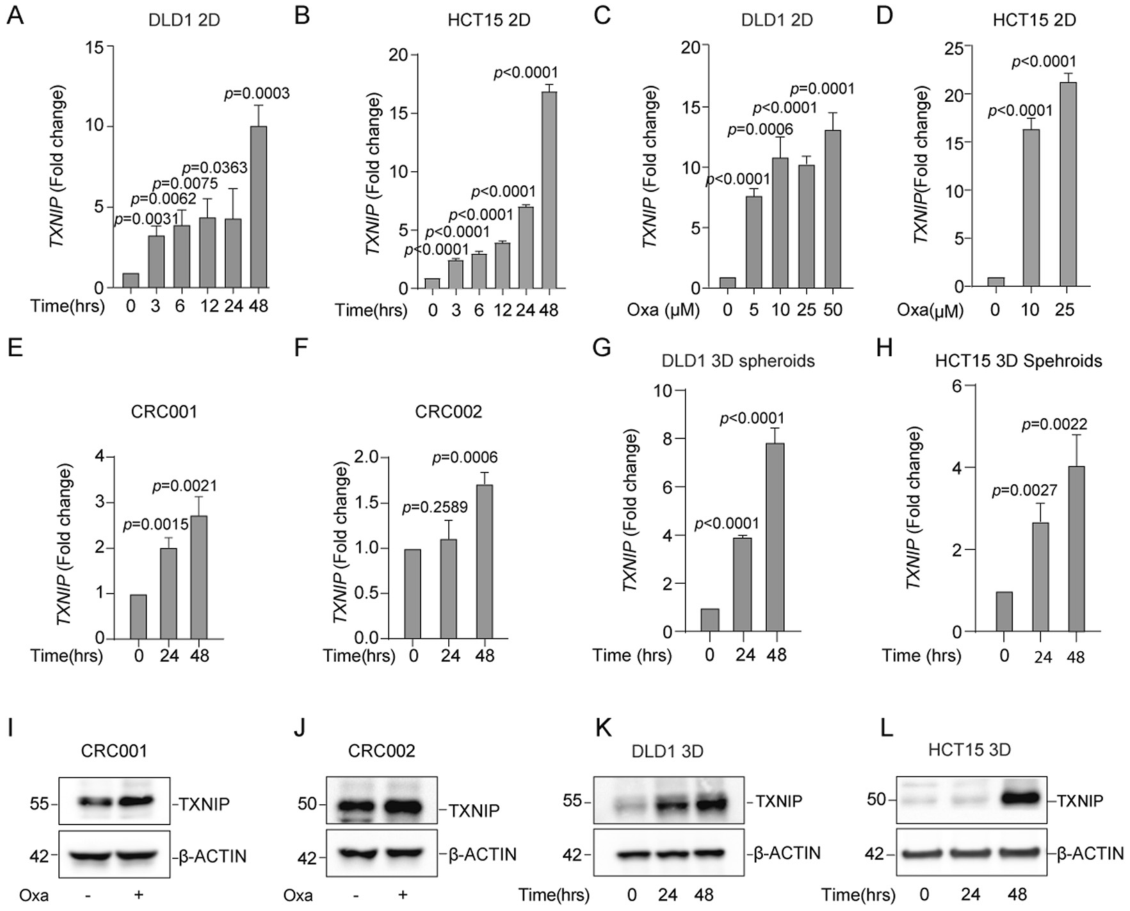


**Figure EV1. TXNIP expression is induced by oxaliplatin in different CRC models.**

(**A**, **B**) Assessment of *TXNIP* mRNA expression in DLD1 cells (**A**) or HCT15 cells (**B**) treated with oxaliplatin by q-RT-PCR analysis. Cells were treated with 10 μM oxaliplatin and harvested at indicated time points. (**C**, **D**) RT-qPCR analysis of *TXNIP* mRNA in DLD1 cells (**C**) or HCT15 cells (**D**) treated with oxaliplatin for 48 h at indicated concentrations. (**E**, **F**) RT-qPCR analysis of *TXNIP* mRNA in two different PDTOs treated with 10 μm oxaliplatin for indicated time periods. (**G**, **H**) RT-qPCR analysis of *TXNIP* mRNA in DLD1 (**G**) or HCT15 (**H**) spheroids treated with 10 μm oxaliplatin for indicated time periods. (**I**, **J**) Western blot analyzes of TXNIP post oxaliplatin treatment (10 μm) in two different PDTOs for 48 h. (**K**, **L**) Western blotting of TXNIP in DLD1 (**K**) or HCT15 (**L**) spheroids treated with 10 μm oxaliplatin for 48 h. Results shown are representative of three independent experiments. All values were expressed as mean ± SEM. *$P < 0.1$, **$P < 0.01$, ***$P < 0.001$, ****$P < 0.0001$, vs. Control. Source data are available online for this figure.

 © The Author(s)

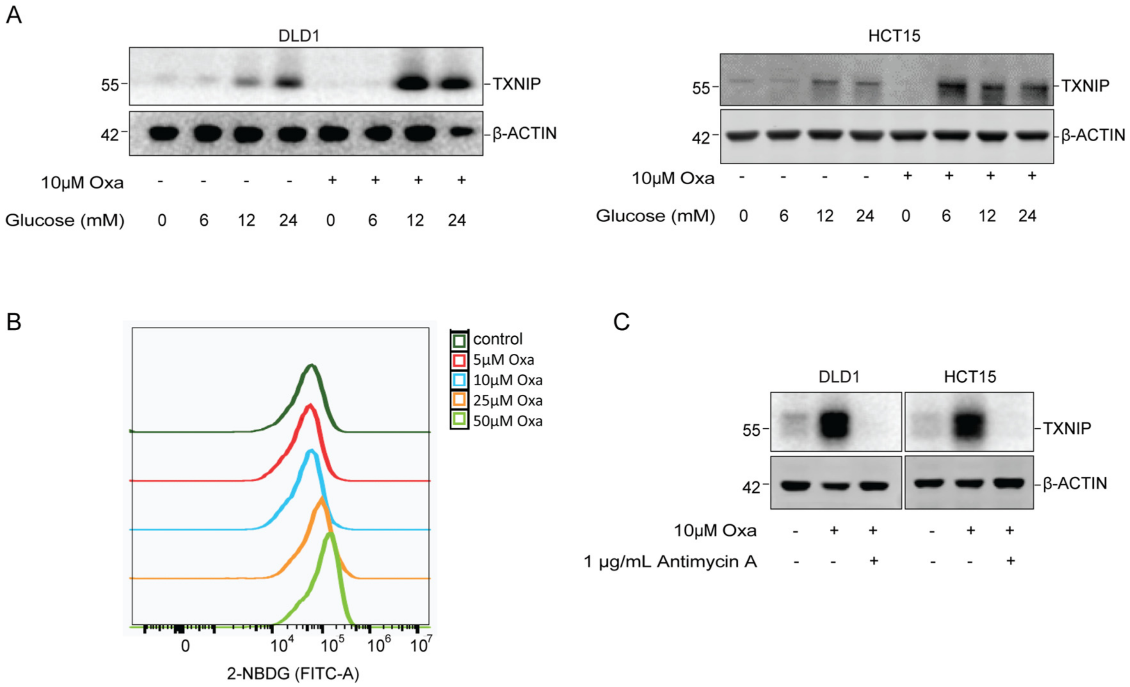


**Figure EV2. The induction of TXNIP by oxaliplatin is dependent on glucose availability, uptake and oxidative phosphorylation.**

(**A**) Western blotting analysis of TXNIP expression in DLD1 cells or HCT15 cells treated with different concentrations of glucose with or without 10 μM oxaliplatin for 48 h. β-actin was used as an internal reference. (**B**) DLD1 cells were treated with different concentration of oxaliplatin or vehicle (PBS) for 48 h. After treatment, 2-NBDG staining and flow cytometry were used to detect glucose uptake. (**C**) Immunoblot analysis of TXNIP in DLD1 cells or HCT15 cells treated with Antimycin A (1 μg/mL), an OXPHOS inhibitor or oxaliplatin (10 μm) or the combinational treatment for 48 h. Results shown are representative of two independent experiments.

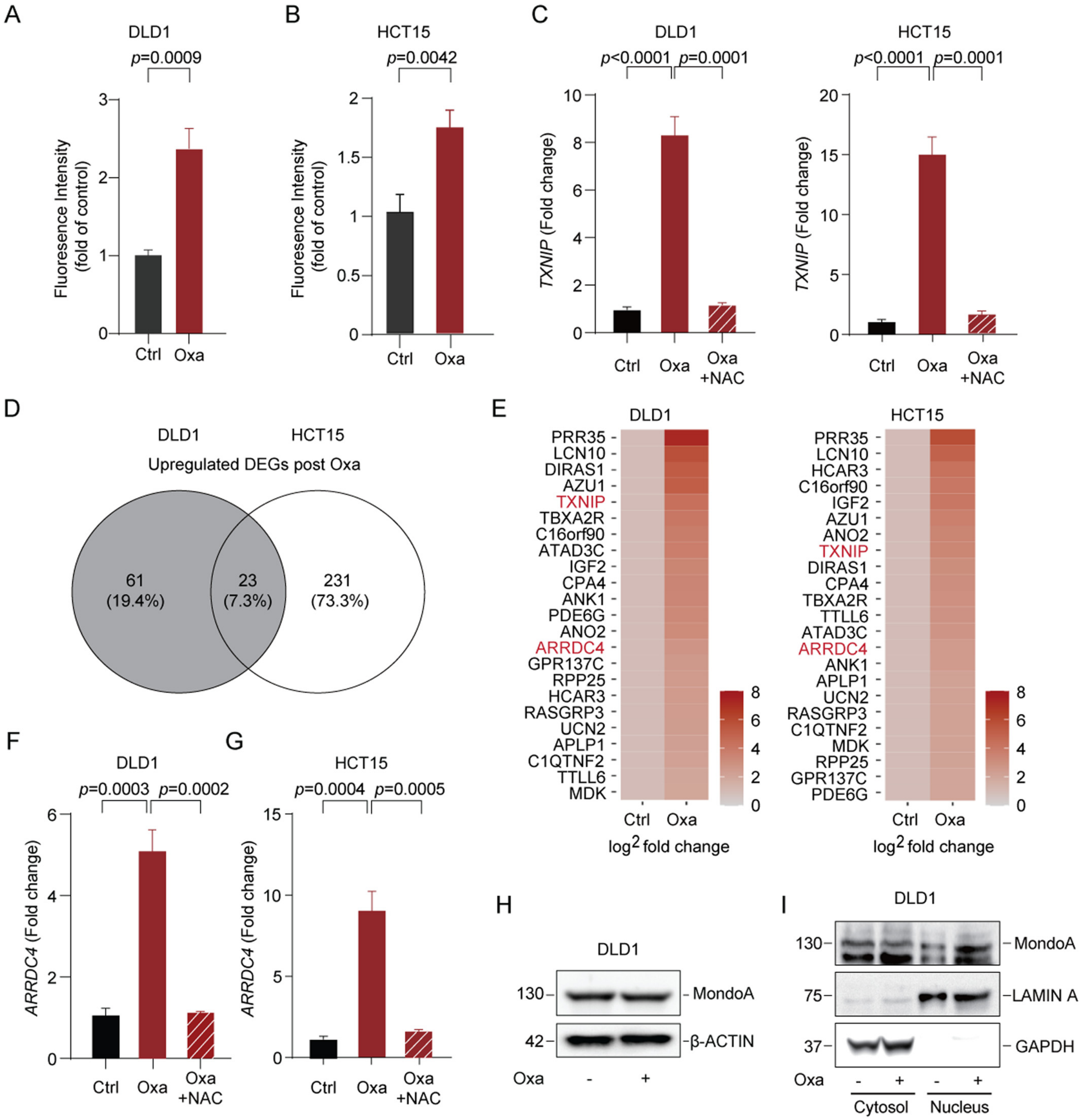


**Figure EV3. ROS drive the induction of TXNIP by inducing MondoA activity.**

(**A**, **B**) DLD1 cells (**A**) and HCT15 cells (**B**) were treated with 10 μm oxaliplatin with ROS measured at 48 h. (**C**) RT-qPCR analysis of *TXNIP* mRNA in DLD1 cells (left panel) or HCT15 cells (right panel) treated with N-acetyl-L-cysteine (NAC) (1.25 mM) or oxaliplatin (10 μm), or combinational treatment, for 48 h. (**D**) Overlapping DEGs ( > 4-fold change; Padj<0.05) from live DLD1 and HCT15 cells, after 48 h of 10 μm oxaliplatin treatment, as determined by RNA sequencing. (**E**) Heatmap showing 23 overlapping transcripts from D, in DLD1 cells (left panel) and HCT15 cells (right panel). (**F**, **G**) RT-qPCR analysis of *ARRDC4* mRNA in DLD1 cells (**F**) and HCT15 cells (**G**) treated with NAC (1.25 mM) or oxaliplatin (10 μm), or combinational treatment, for 48 h. (**H**) Immunoblot analysis of MondoA expression in DLD1 cells after 10 μm oxaliplatin treatment for 48 h. (**I**) Effects of oxaliplatin treatment (10 μm for 48 h) on subcellular localization of MondoA assessed by cell fractionation and immunoblotting, in DLD1 cells. LAMIN A - nuclear marker, GAPDH - cytoplasmic marker. Results shown (excluding **D** and **E**) are representative of three independent experiments. All values were expressed as mean ± SEM. $^{**}P < 0.01$, $^{***}P < 0.001$, $^{****}P < 0.0001$, vs. Control. Source data are available online for this figure.

 © The Author(s)

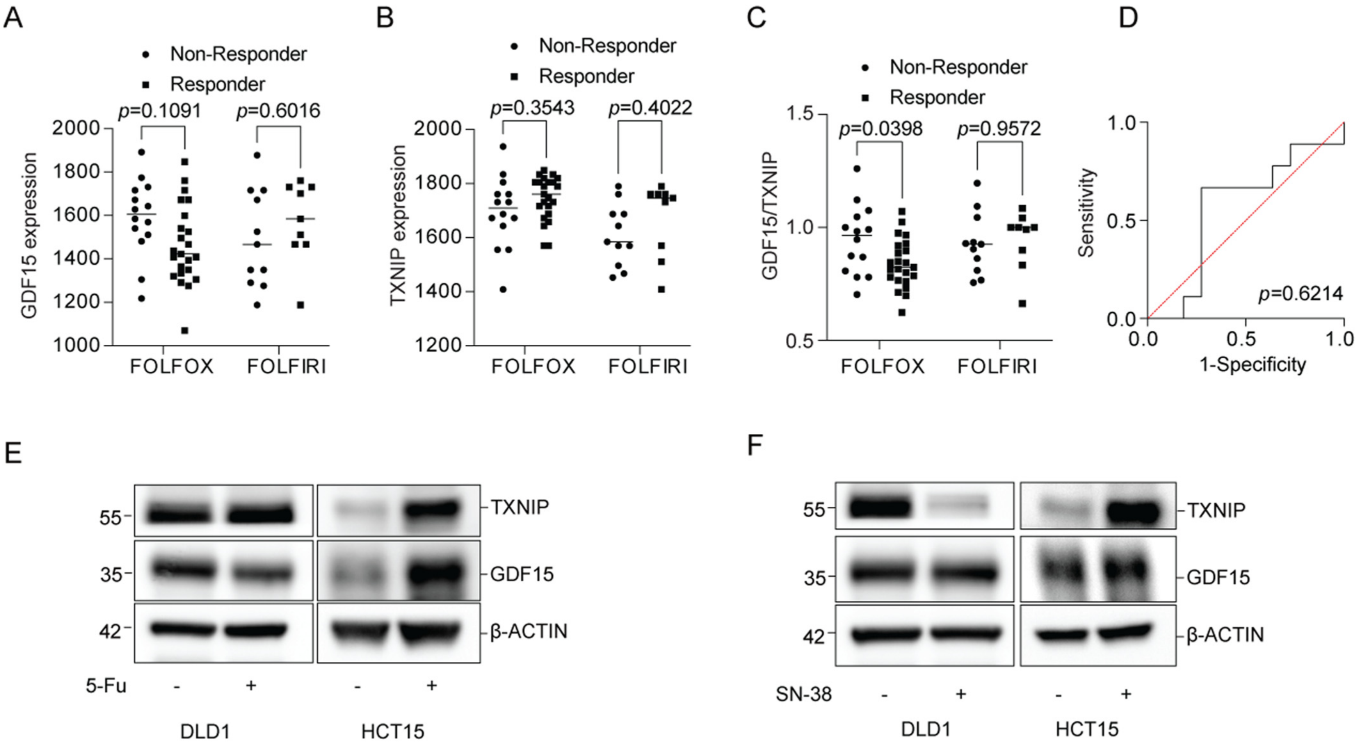


**Figure EV4. The described effects are specific for oxaliplatin.**

*GDF15* (**A**) or *TXNIP* (**B**) expression in responders or non-responders to FOLFOX or FOLFIRI treatment. (**C**) *GDF15/TXNIP* expression ratio in FOLFOX or FOLFIRI treated responders or non-responders. (**D**) Receiver operating characteristic (ROC) curve showing area under the curve and *P* value for the use of *GDF15/TXNIP* ratio in predicting therapeutic response to FOLFIRI (Responder [$n = 9$] nonresponder [$n = 11$]). (**E**) Immunoblot analysis of TXNIP and GDF15 after 48 h of 10 μm 5-Fu treatment in DLD1 and HCT15 cell lines. (**F**) Immunoblot analysis of TXNIP and GDF15 expression after 48 h of 10 μm SSN-38 treatment in DLD1 and HCT15 cell lines. (**E**, **F**) were repeated in three independent experiments. Two-way ANOVA, multiple comparisons test. *$P < 0.05$. Source data are available online for this figure.

© The Author(s)

A

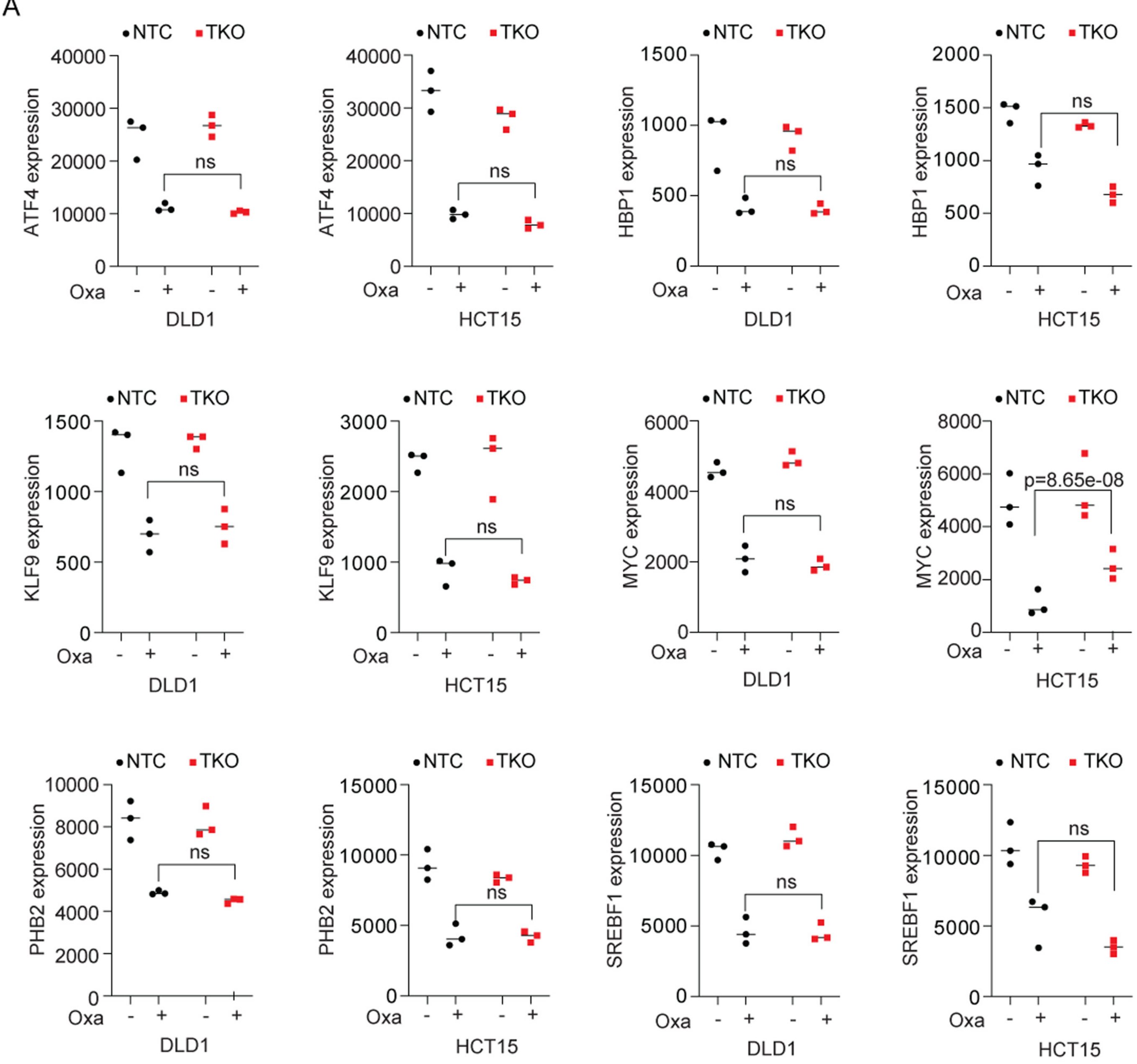


B

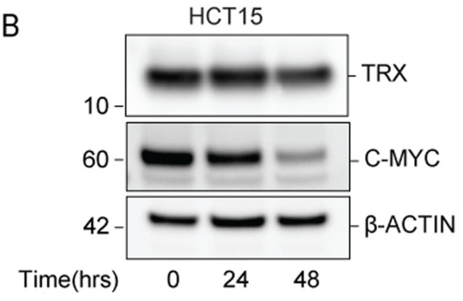


C

| | TXNIP | | GDF15 | |
|---|---|---|---|---|
| | r | p value | r | p value |
| MYC | -0.4631 | 1.42E -28 | 0.4653 | 7.32E -29 |
| ATF4 | -0.3271 | 3.13E -14 | 0.3261 | 3.76E -14 |
| PHB2 | -0.2401 | 3.77E -08 | 0.3552 | 1.14E -16 |
| SREBF1 | -0.06604 | 0.1357 | 0.296 | 8.15E -12 |
| HBP1 | 0.4756 | 3.03E -30 | -0.3269 | 3.23E -14 |
| KLF9 | 0.5044 | 2.19E -34 | -0.4355 | 4.21E -25 |

 © The Author(s)

**Figure EV5. C-MYC is downregulated by oxaliplatin in HCT15 cells in a TXNIP-dependent manner.**

(**A**) Transcript expression of GDF15 binding TFs (*ATF4, HBP1, KLF9, MYC, PHB2* and *SREBF1*) in control (NTC) and *TXNIP*-KO (TKO) cells (DLD1 or HCT15) with/ without 10 µm oxaliplatin treatment for 48 h. Each data point represents an biological replicate. (**B**) Western blotting analysis of TRX and C-MYC expression in HCT15 cells treated with 10 mM oxaliplatin at different time points. β-actin was used as an internal reference. (**C**) Correlations between the indicated TFs and *TXNIP* and *GDF15* in the TCGA COAD dataset. R and *P* values shown (Pearson's). (**B**) was repeated in 2 independent experiments. Source data are available online for this figure.
